# Atezolizumab in Combination With Carboplatin and Survival Outcomes in Patients With Metastatic Triple-Negative Breast Cancer: The TBCRC 043 Phase 2 Randomized Clinical Trial

**DOI:** 10.1001/jamaoncol.2023.5424

**Published:** 2023-12-14

**Authors:** Brian D. Lehmann, Vandana G. Abramson, E. Claire Dees, Payal D. Shah, Tarah J. Ballinger, Claudine Isaacs, Cesar A. Santa-Maria, Hanbing An, Paula I. Gonzalez-Ericsson, Melinda E. Sanders, Kimberly C. Newsom, Richard G. Abramson, Quanhu Sheng, Chih-Yuan Hsu, Yu Shyr, Antonio C. Wolff, Jennifer A. Pietenpol

**Affiliations:** 1 Department of Medicine, Vanderbilt University, Nashville, Tennessee; 2 Vanderbilt-Ingram Cancer Center, Vanderbilt University Medical Center, Tennessee; 3 Department of Medicine and Lineberger Comprehensive Cancer Center, University of North Carolina, Chapel Hill; 4 Department of Medicine, University of Pennsylvania, Philadelphia; 5 Department of Medicine, Indiana University, Indianapolis; 6 Department of Medical Oncology, Lombardi Cancer Center, Georgetown University, Washington, DC; 7 Department of Medicine, Johns Hopkins School of Medicine, Baltimore, Maryland; 8 Department of Otolaryngology, Vanderbilt University Medical Center, Nashville, Tennessee; 9 Breast Cancer Research Program, Vanderbilt University Medical Center, Nashville, Tennessee; 10 Department of Pathology, Microbiology and Immunology, Vanderbilt University, Nashville, Tennessee; 11 Department of Biomedical Engineering, Vanderbilt University School of Engineering, Nashville, Tennessee; 12 Department of Biostatistics, Vanderbilt University Medical Center, Nashville, Tennessee; 13 Center for Quantitative Sciences, Vanderbilt University Medical Center, Nashville, Tennessee; 14 Department of Biochemistry, Vanderbilt University, Nashville, Tennessee

## Abstract

**Importance:**

Agents targeting programmed death ligand 1 (PD-L1) have demonstrated efficacy in triple-negative breast cancer (TNBC) when combined with chemotherapy and are now the standard of care in patients with PD-L1–positive metastatic disease. In contrast to microtubule-targeting agents, the effect of combining platinum compounds with programmed cell death 1 (PD-1)/PD-L1 immunotherapy has not been extensively determined.

**Objective:**

To evaluate the efficacy of atezolizumab with carboplatin in patients with metastatic TNBC.

**Design, Setting, and Participants:**

This phase 2 randomized clinical trial was conducted in 6 centers from August 2017 to June 2021.

**Interventions:**

Patients with metastatic TNBC were randomized to receive carboplatin area under the curve (AUC) 6 alone or with atezolizumab, 1200 mg, every 3 weeks until disease progression or unacceptable toxic effects with a 3-year duration of follow-up.

**Main Outcome and Measures:**

The primary end point was investigator-assessed progression-free survival (PFS). Secondary end points included overall response rate (ORR), clinical benefit rate (CBR), and overall survival (OS). Other objectives included correlation of response with tumor PD-L1 levels, tumor-infiltrating lymphocytes (TILs), tumor DNA- and RNA-sequenced biomarkers, TNBC subtyping, and multiplex analyses of immune markers.

**Results:**

All 106 patients with metastatic TNBC who were enrolled were female with a mean (range) age of 55 (27-79) years, of which 12 (19%) identified as African American/Black, 1 (1%) as Asian, 73 (69%) as White, and 11 (10%) as unknown. Patients were randomized and received either carboplatin (n = 50) or carboplatin and atezolizumab (n = 56). The combination improved PFS (hazard ratio [HR], 0.66; 95% CI, 0.44-1.01; *P* = .05) from a median of 2.2 to 4.1 months, increased ORR from 8.0% (95% CI, 3.2%-18.8%) to 30.4% (95% CI, 19.9%-43.3%), increased CBR at 6 months from 18.0% (95% CI, 9.8%-30.1%) to 37.5% (95% CI, 26.0%-50.6%), and improved OS (HR, 0.60; 95% CI, 0.37-0.96; *P* = .03) from a median of 8.6 to 12.6 months. Subgroup analysis showed PD-L1–positive tumors did not benefit more from adding atezolizumab (HR, 0.62; 95% CI, 0.23-1.65; *P* = .35). Patients with high TILs (HR, 0.12; 95% CI, 0.30-0.50), high mutation burden (HR, 0.50; 95% CI, 0.23-1.06), and prior chemotherapy (HR, 0.59; 95% CI, 0.36-0.95) received greater benefit on the combination. Patients with obesity and patients with more than 125 mg/dL on-treatment blood glucose levels were associated with better PFS (HR, 0.35; 95% CI, 0.10-1.80) on the combination. TNBC subtypes benefited from adding atezolizumab, except the luminal androgen receptor subtype.

**Conclusions and Relevance:**

In this randomized clinical trial, the addition of atezolizumab to carboplatin significantly improved survival of patients with metastatic TNBC regardless of PD-L1 status. Further, lower risk of disease progression was associated with increased TILs, higher mutation burden, obesity, and uncontrolled blood glucose levels.

**Trial Registration:**

ClinicalTrials.gov Identifier: NCT03206203

## Key Points

**Question:**

Is the efficacy of carboplatin increased with atezolizumab in metastatic triple-negative breast cancer (TNBC), and are there clinical or molecular correlates associated with response?

**Findings:**

In this randomized clinical trial including 106 patients, combining atezolizumab with carboplatin significantly improved progression-free and overall survival of patients with metastatic TNBC; tumor-infiltrating lymphocytes, mutation burden, obesity, and increased blood glucose levels were associated with response to anti–programmed cell death ligand 1 therapy.

**Meaning:**

Adding atezolizumab to carboplatin provided a clinically meaningful benefit to patients with metastatic TNBC.

## Teaser

This randomized clinical trial examines the efficacy of adding atezolizumab to treatment with carboplatin in patients with metastatic triple-negative breast cancer (TNBC), and the clinical or molecular correlates associated with disease response.

## Introduction

Triple-negative breast cancer (TNBC) is a heterogeneous disease characterized by increased genomic instability, immune infiltration,^1^ and high programmed death ligand 1 (PD-L1) expression.^2^ These features provided the rationale for early clinical studies evaluating the efficacy of anti–PD-L1 monoclonal antibody atezolizumab as monotherapy or in combination with nab-paclitaxel in advanced TNBC.^3,4^ The durable responses, particularly in PD-L1–positive tumors, led to the phase 3 IMpassion130 trial and accelerated US Food and Drug Administration (FDA) approval for PD-L1–positive metastatic TNBC.^3^ A subsequent phase 3 trial evaluated atezolizumab in combination with paclitaxel; however, this combination did not improve progression-free survival (PFS) or overall survival (OS) vs paclitaxel alone in PD-L1–positive metastatic TNBC.^5^ The reasons for this are unclear and suggest that anti–PD-L1 therapy efficacy may differ when combined with different chemotherapy. Anti–PD-L1 therapy was first approved in combination with taxane chemotherapy.^6^ Unlike taxanes, platinum agents are DNA intercalating agents and generate neoantigens that may stimulate an immune response.^7^ Therefore, the study reported herein, TBCRC 043, was designed to prospectively evaluate the efficacy of adding the anti–PD-L1 antibody atezolizumab to carboplatin therapy.

TNBC is a heterogeneous disease with varying immune cell composition and may have differential sensitivity to immune checkpoint therapies.^8,9^ Although several biomarkers, such as tumor mutation burden (TMB)^10,11^ and tumor-infiltrating lymphocytes (TILs),^12^ are associated with better response, there is a need to identify patients who are unlikely to respond to immunotherapy and spare them from severe immune-related toxic effects.^13^ We thus present clinical trial results as well as molecular correlates of response to atezolizumab in combination with carboplatin.

## Methods

### Study Design and Participants

TBCRC 043 is a prospective, multicenter, randomized, double-blind phase 2 trial (NCT03206203**)** investigating the efficacy of atezolizumab in combination with carboplatin vs carboplatin alone in patients with metastatic TNBC. Eligible patients had either clinical stage IV or metastatic invasive breast cancer negative for estrogen receptor (<10%), progesterone receptor (<10%), and *ERBB2* (immunohistochemical analysis, ≥0/1 or fluorescence in situ hybridization, <2.0). Patients with an Eastern Cooperative Oncology Group performance status of 0 to 1, measurable disease, adequate hematologic, kidney, hepatic, and cardiac function, 0 to 1 prior treatments for metastatic disease, and no prior carboplatin in the metastatic setting or prior immune-oncology treatment were eligible. Patients were not stratified by PD-L1 status.

The protocol (Supplement 1) was approved by ethical and institutional review boards (IRB#160633) at the participating institutions, and all patients provided written informed consent and did not receive financial compensation. Patients were screened and enrolled at participating centers of the Translational Breast Cancer Research Consortium (TBCRC). Data were collected and reviewed by the Clinical Trials Office and Data and Safety Monitoring Committee.

### Treatments and End Points

Patients were randomized to receive intravenous carboplatin area under the curve (AUC) 6 alone or in combination with atezolizumab, 1200 mg, every 3 weeks until disease progression, unacceptable toxic effects, or withdrawal of consent. On disease progression (clinically/Response Evaluation Criteria in Solid Tumors), patients on the carboplatin-alone arm were allowed to cross over to receive atezolizumab alone after undergoing a metastatic biopsy if reasonably safe (eFigure 1 in Supplement 2).

The primary end point was PFS, the time from the first day of treatment to disease progression or death. Secondary end points were overall response rate (ORR; CR + PR) and clinical benefit rate (CBR; CR + PR + stable disease ≥6 months), duration of response (DOR), and OS. Adverse events were graded using the National Cancer Institute Common Terminology Criteria for Adverse Events, V5.0.

### Statistical Analysis

The primary PFS end point was powered for a 1-sided test of the hazard ratio (HR) for PFS at α = .10. The Kaplan-Meier method was used to estimate time-to-event curves and medians for PFS, OS, and DOR. Stratified log-rank tests were used to determine significance. Cox proportional hazards regression models were used to estimate HRs and 95% CIs. Data cutoff was October 2021*.* Additional details of the statistical analysis are given in the eMethods in Supplement 2. The analysis was performed using R statistical software (version 4.3.0, R Project for Statistical Computing) and followed the Consolidated Standards of Reporting Trials (CONSORT) reporting guidelines.

## Results

### Study Design and Participants

**Figure 1.**
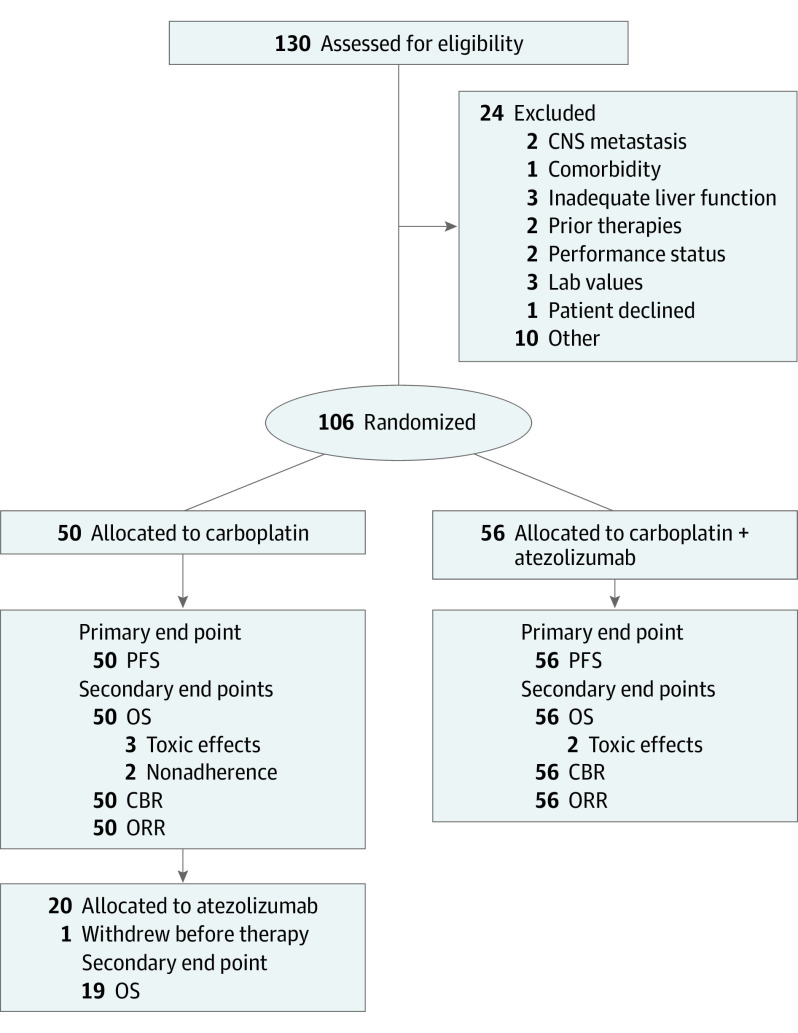
CONSORT Diagram CBR indicates clinical benefit rate; CNS, central nervous system; ORR, overall response rate; OS, overall survival; PFS, progression-free survival.

From August 8, 2017, through October 6, 2020, 130 patients with metastatic TNBC were enrolled on study from 6 participating sites (eFigure 1 in Supplement 2). Twenty-four patients were excluded, and 106 were randomized to receive atezolizumab plus carboplatin (n = 56) or carboplatin (n = 50) (Figure 1). Five patients receiving carboplatin and 2 on the combination arm were removed from the study for reasons other than disease progression.

All study participants’ sex was female, and demographic characteristics were well-balanced between treatment arms (eTable 1 in Supplement 2). The mean (range) age of participants was 55 (27-79) years, 12 of whom identified as African American/Black (19%), 1 as Asian (1%), 73 as White (69%), and 11 as unknown (10%). PD-L1 positive (>1%) rates were similar between the atezolizumab plus carboplatin (10 [18%]) and carboplatin (10 [20%]) arms. Most (87 [82%]) patients received prior chemotherapy, with 35 patients receiving adjuvant alone (33%), both neoadjuvant and adjuvant (22 [20%]), or metastatic treatment (34 [32%]). Of those receiving metastatic treatment, 12 patients (35%) received treatment as first line. Of the patients who had received prior chemotherapy, 15 (14%) had received a prior platinum agent.

### Safety and Tolerability

The median (range) duration of treatment for patients receiving atezolizumab plus carboplatin was 17.4 (1.4-90.3) weeks, whereas for single-agent carboplatin it was 15.4 (3.0-72.1) weeks. The combination was generally well tolerated, and toxic effects were consistent with previous reports.^5,6^ The most common adverse events (AEs, >1) on the combination arm were thrombocytopenia, anemia, lymphocytopenia, nausea, fatigue, and increased liver enzymes (eTable 2 in Supplement 2). Compared with carboplatin, atezolizumab plus carboplatin was associated with a higher incidence of grade 3/4 serious AEs (41% vs 8%). Grade 3 immune-related AEs occurred in 10 patients (5%) receiving the combination, and among possible immune-related AEs were individual cases of colitis, hypothyroidism, retinopathy, and infective myositis (eTable 2 in Supplement 2). Even with increased serious AEs, the withdrawal of study drugs was similar between arms, with 3 patients (6%) receiving carboplatin and 2 patients (4%) receiving the combination coming off study for toxic effects. Crossover patients receiving atezolizumab monotherapy had lower serious AEs (21%) than the combination arm.

### Efficacy

**Figure 2.**
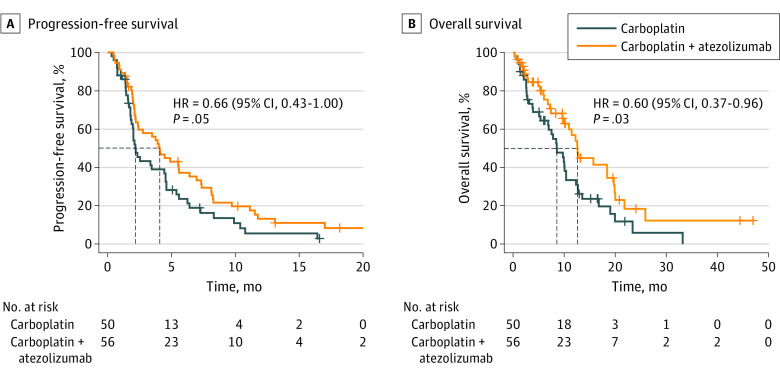
Kaplan-Meier Estimate of Progression-Free Survival (PFS) and Overall Survival (OS) A, Investigator-assessed PFS of patients stratified by treatment arm. The median PFS in the carboplatin arm was 2.2 months vs 4.1 months in the atezolizumab plus carboplatin arm. B, OS of patients receiving only carboplatin or carboplatin plus atezolizumab. The median OS in the carboplatin arm was 8.6 months vs 12.6 months in the atezolizumab and carboplatin arm. HR indicates hazard ratio. Data cutoff was October 2021.

The data cutoff for PFS was October 2021. At this date, 94 patients (88.7%) experienced disease progression or death and the median (range) duration of follow-up was 8.9 (0.9-33.2) and 10.3 (0.9-30.0) months for carboplatin and the combination, respectively. Adding atezolizumab to carboplatin significantly (HR, 0.66; 95% CI, 0.44-1.01; log-rank *P* = .05) improved PFS (Figure 2A). Median PFS was 4.1 (95% CI, 2.4-7.0) months with atezolizumab plus carboplatin compared with 2.2 (95% CI, 2.0-4.4) months with carboplatin (eTable 3 in Supplement 2).

Overall, 106 patients were evaluable for CBR at 6 months and ORR with 7 treatment failures due to toxic effects/nonadherence (Figure 1). The secondary end point of ORR was increased from 8.0% (95% CI, 3.2%-18.8%) for patients on the carboplatin arm to 30.4% (95% CI, 19.9%-43.3%) for the combination (eTable 3 in Supplement 2). The CBR was 18.0% (95% CI, 9.8%-30.1%) for carboplatin and 37.5% (95% CI, 26.0%-50.6%) for patients receiving the combination (eTable 1 in Supplement 2). Among responding patients, the median duration of response was similar between arms (carboplatin, 14.8 months; 95% CI, 8.2-Inf; combination, 11.6 months; 95% CI, 7.6-17.7) (eTable 3 and eFigure 2B in Supplement 2).

At the cutoff date, the addition of atezolizumab to carboplatin was associated with significantly improved OS in the intent-to-treat population (HR, 0.60; 95% CI, 0.37-0.96; log-rank *P* = .03) from 8.6 to 12.6 months (Figure 2B). Furthermore, when crossover patients were left out of the analysis, OS improved more significantly (HR, 0.46; 95% CI, 0.27-0.79; log-rank *P* = .004) from 7.0 (95% CI, 3.2-10.1) months to 12.6 (95% CI, 10.9-19.9) months (supporting data in eFigure 2C in Supplement 2). Twenty patients who had disease progression while receiving carboplatin enrolled on the crossover arm, with 19 receiving atezolizumab monotherapy (eFigure 1 in Supplement 2). OS was not significantly different (log-rank *P* = .62) between those patients who received the combination and those who received both monotherapies sequentially (Supporting data in eFigure 2D in Supplement 2).

### Clinical Correlates

**Figure 3.**
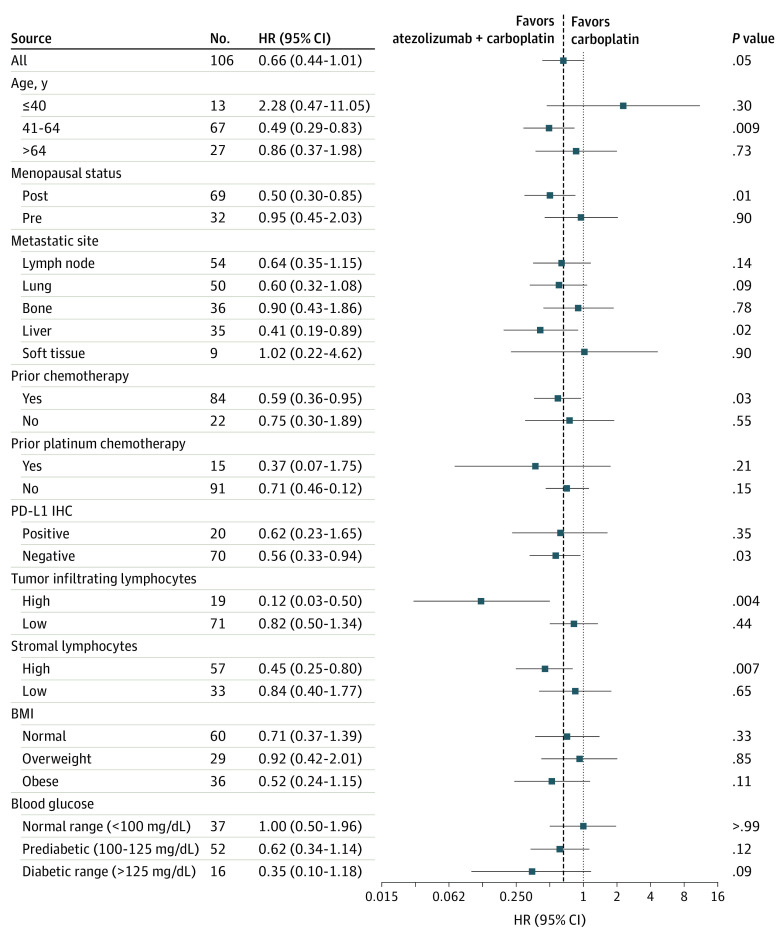
Subgroup Analysis of Progression-Free Survival (PFS) in Patients Treated With Carboplatin or Carboplatin Plus Atezolizumab Squares indicate PFS hazard ratios (HRs) from univariate Cox regression models, and 95% CIs are indicated by crossing horizontal lines. The dashed line is the reference HR (0.66) for all patients comparing the 2 treatments. BMI indicates body mass index (calculated as weight in kilograms divided by height in meters squared); PD-L1, programmed death ligand 1.

To better understand clinical variables associated with response, we estimated HRs between the arms using PFS. The overall HR favored carboplatin plus atezolizumab (HR, 0.66; *P* = .05) vs carboplatin alone (Figure 3). Women between the ages of 41-64 years (HR, 0.49; *P* = .009), postmenopausal women (HR, 0.50; *P* = .01), patients with liver metastases (HR, 0.41; *P* = .02), and patients who received any prior chemotherapy (HR, 0.59; *P* = .03) significantly benefited from adding atezolizumab.

An independent reference lab performed PD-L1 IHC (Ventana SP142), and 22.2% (20 of 90) of specimens were positive (>1%) for PD-L1 (eTable 4 in Supplement 3). Of these, most (90%) had PD-L1 positivity only in stromal/immune cells, with only 2 specimens with PD-L1–positive tumor cells. PD-L1 expression differed by metastatic site, with liver (*P* = .003), bone (*P* = .005), and brain (*P* = .05) significantly lower than other sites (supporting data in eFigure 3A in Supplement 2). There was no significant (*P* = .80) difference between the PD-L1 staining and treatment arm (supporting data in eFigure 3B in Supplement 2). Patients with PD-L1–positive tumors did not receive greater benefit on either arm (eFigure 3C-3E in Supplement 2). However, adding atezolizumab was associated with significantly improved PFS and OS in patients with PD-L1–negative tumors (eFigure 3F and 3G in Supplement 2).

TILs have been correlated with immune checkpoint response, and therefore we quantified both intratumor TILs (iTILs) and stromal TILs (sTILs) in pretreatment tumor biopsies.^14^ Both iTILs and sTILs varied by metastatic biopsy site, with lung, liver, and bone having lower percentages than other sites (eFigure 4A and 4B in Supplement 2). Tumor biopsies were stratified into high (>5%) and low (<5%) iTILs (eFigure 4C and 4D in Supplement 2). Both high baseline iTILs (HR, 0.12; *P* = .004) or sTILs (HR, 0.45; *P* = .007) were associated with a lower rate of progression when receiving the combination compared with carboplatin alone (Figure 3; eFigure 4E-4H in Supplement 2).

Because obesity and diabetes are linked to systemic inflammation,^15^ we calculated individual patients’ body mass index (BMI, calculated as weight in kilograms divided by height in meters squared) and mean on-treatment blood glucose values. More than 60% of patients had overweight (BMI, 25-30; 29 [27.6%]) or obesity (BMI >30; 30 [34.2%]), with the remaining in the normal range (BMI <25; 40 [38.1%]) (eFigure 5A in Supplement 2). While receiving therapy, more than 60% of patients had average blood glucose levels within prediabetic (100-125 mg/dL; 49.0%) and diabetic levels (>125 mg/dL; 15.0%), whereas approximately one-third had normal levels (<100 mg/dL) (eFigure 5A in Supplement 2). These levels likely reflect uncontrolled glucose levels because 50% of the patients in the diabetic range and no patients in the normal range were taking antidiabetic medicines. There were no significant differences in mean (SD) BMI (27.4 [7.5] vs 29.3 [7.3]; *P* = .33) or mean (SD) blood glucose levels (114.5 [37.4] vs 109.8 [20.7] mg/dL; *P* = .39) between treatment arms (eFigure 5B and 5C in Supplement 2). However, there was a trend toward greater benefit from the combination for patients with obesity (HR, 0.52; *P* = .10) and patients with uncontrolled blood glucose levels at prediabetic (HR, 0.62; *P* = .13) and diabetic (HR, 0.35; *P* = .09) levels (Figure 3; eFigure 5D and 5E in Supplement 2). Because blood glucose levels varied through treatment, a time-dependent Cox regression analysis was performed and showed glucose levels were associated with worse PFS and OS for patients receiving carboplatin (eFigure 5F and 5G in Supplement 2).

### Molecular Correlates

This trial was designed for extensive exploratory biomarker analysis to identify patient subgroups with response to atezolizumab that included: (1) tumor and immune PD-L1 expression, (2) nonsynonymous mutation burden rate, (3) TNBC molecular subtype, and (4) lymphocytes composition by multichannel immunofluorescence (eMethods in Supplement 2).

To identify differentially expressed transcripts and evaluate TNBC molecular subtypes associated with response, we performed RNA sequencing on pretreatment tumor biopsies. After adjusting for metastatic tissue site and sample type (fresh frozen vs formalin-fixed paraffin-embedded), we identified 82 differentially expressed transcripts between patients responding (partial response or stable disease) to carboplatin plus atezolizumab and those with progressive disease (eFigure 6A-6C, eTable 5 in Supplement 3). Gene ontology analysis of transcripts elevated in responders showed enrichment in insulin-like growth factor (IGF) transport and uptake, and lipid pathways (eTable 5 in Supplement 3). IGF2, a growth factor capable of binding the insulin receptor and implicated in diabetes,^16^ was among the highest transcripts expressed in responding patient tumors (eFigure 6D in Supplement 2).

**Figure 4.**
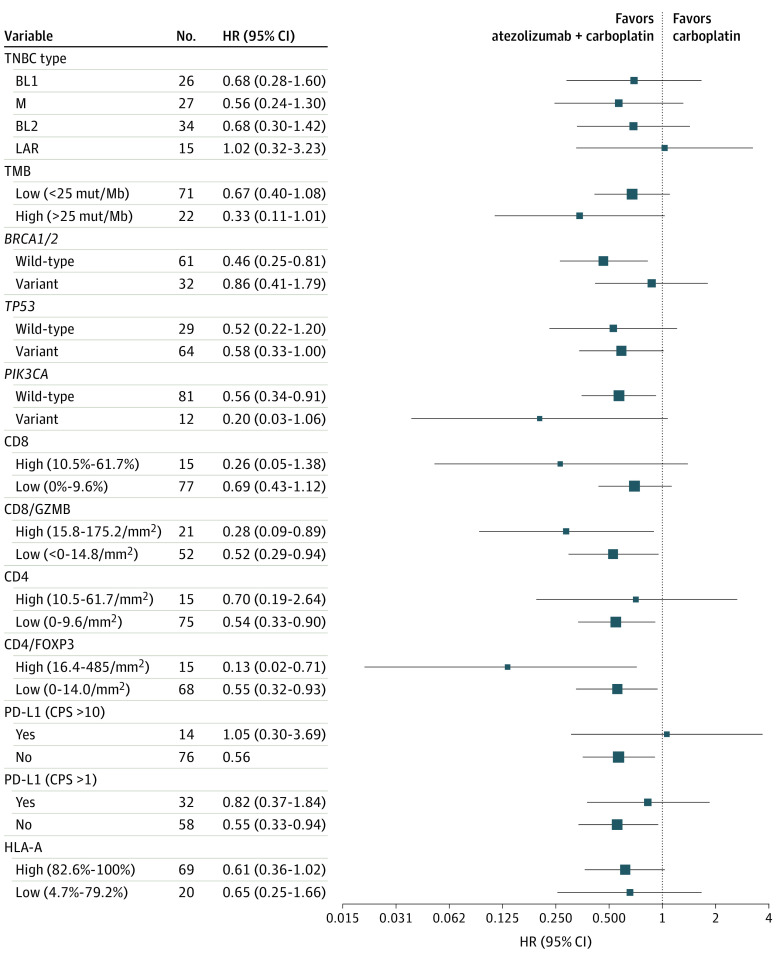
Subgroup Analysis of Progression of Patients Between Treatment Arms Forest plot shows the hazard ratios (HRs). Between variable cutoffs on each arm stratified by TNBC type, DNA mutation, and multiplex immunofluorescence markers. BL1 indicates basal-like 1; BL2, basal-like 2; M, mesenchymal; LAR, luminal androgen receptor; TMB, tumor mutation burden; CPS, combined positive score.

TNBC subtyping was performed on RNA sequencing from 102 pretreatment biopsies, which resulted in 25% basal-like 1 (BL1), 26% mesenchymal (M), 33% basal-like 2 (BL2), and 15% luminal androgen receptor (LAR) (eFigure 6E in Supplement 2). This distribution in metastatic disease differs from primary TNBC in the Cancer Genome Atlas (35% BL1, 30% M, 20% BL2, and 15% LAR) and likely reflects the reduction of the chemosensitive BL1 subtype from prior chemotherapy treatments (eFigure 6F in Supplement 2).^17,18^ All subtypes tended to benefit more with the addition of atezolizumab, except for the LAR subtype (Figure 4; eFigure 6G in Supplement 2).

Because variants generate neoantigens, we performed whole genome DNA sequencing on pretreatment tumor tissues to identify somatic variants. The median nonsynonymous mutation rate ranged from 9.1-371.0 mutations/megabase (MB) with a median (SD) of 15.7 (40.1) mut/Mb (eFigure 7A in Supplement 2). There was no association between TMB and PFS for patients receiving carboplatin (log-rank *P* = .63); however, patients with high TMB (>25 mut/Mb) who received atezolizumab and carboplatin had better PFS (log-rank *P* = .05) (supporting data in eFigure 7B and 7C in Supplement 2).

**Figure 5.**
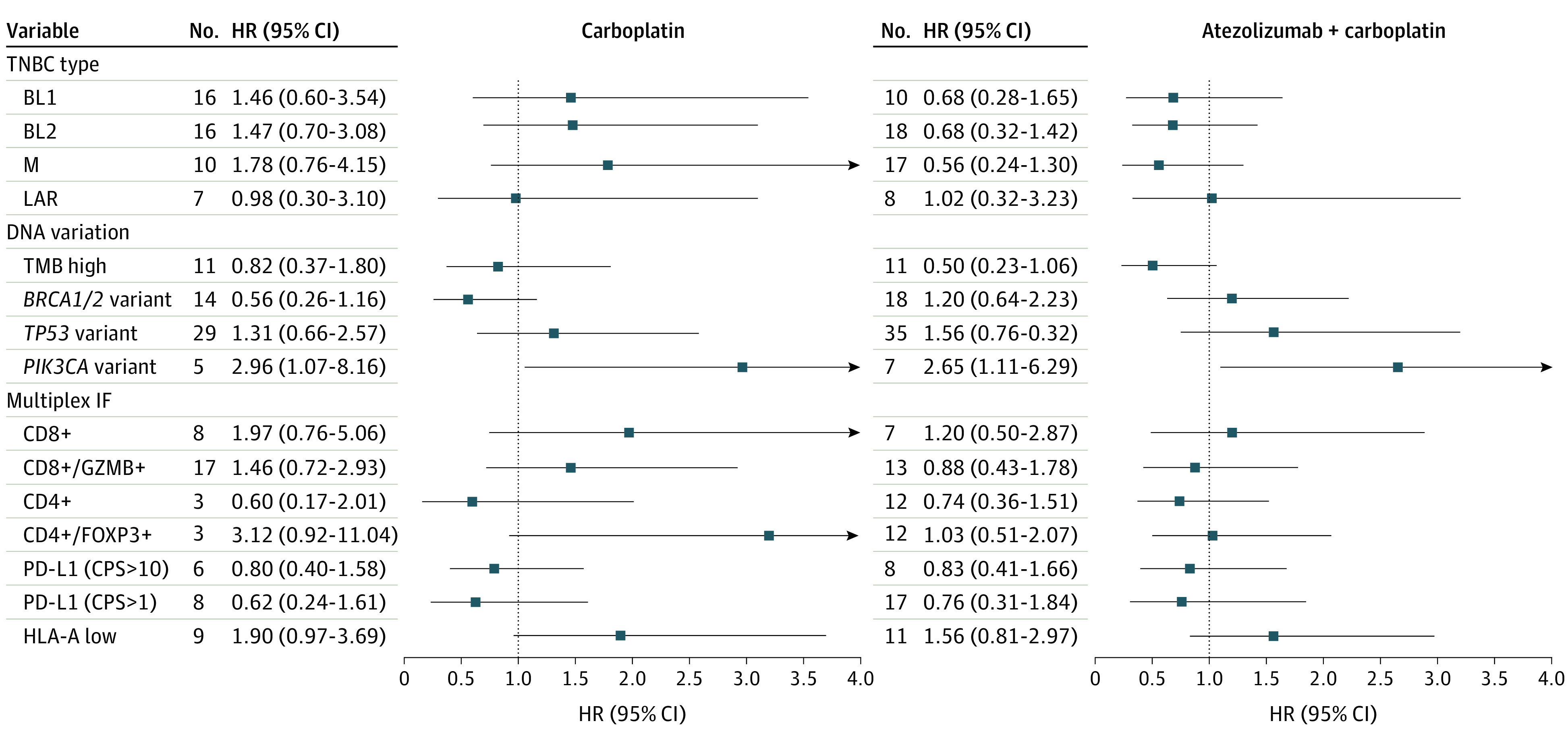
Subgroup Analysis of Molecular Correlates Associated With Progression-Free Survival Forest plot shows the hazard ratio (HR) for the progression of patients between variable cutoffs on each arm stratified by TNBC type, DNA mutation, and multiplex immunofluorescence markers. BL1 indicates basal-like 1; BL2, basal-like 2; M, mesenchymal; LAR, luminal androgen receptor; TMB, tumor mutation burden; CPS, combined positive score.

As with previous studies, TNBC tumors displayed relatively few recurrent mutations (eFigure 7D in Supplement 2).^19,20^ *TP53* was the most frequently (68.8%) mutated gene (eFigure 8A in Supplement 2). Patients with *TP53* mutant tumors tended to have less favorable outcomes to either carboplatin (HR, 1.3; 95% CI, 0.6-2.6) or the combination (HR, 1.6; 95% CI, 0.8-3.2) (Figure 4; eFigure 8B in Supplement 2). *BRCA1* was mutated in 15.1%, and *BRCA2* mutated in 21.3% of tumors (eFigure 8C in Supplement 2). Patients with *BRCA1/2*-mutated tumors tended to respond better to carboplatin (HR, 0.86; 95% CI, 0.41-1.79) (Figure 5; eFigure 8D in Supplement 2). Activating *PIK3CA* mutations were identified in 13% of tumors, with most (63.6%) occurring in the LAR subtype, consistent with prior studies (eFigure 8E in Supplement 2).^21,22^ Patients with activating *PIK3CA* variants had significantly worse survival to either carboplatin alone (HR, 2.96; *P* = .04) or the combination (HR, 2.65; *P* = .03) (Figure 4; eFigure 8F in Supplement 2).

Because TILs were associated with response to atezolizumab plus carboplatin, we developed multiplex-immunofluorescence assays to evaluate tumor-specific (pan-CK), PD-L1, and MHC-I expression, as well as effector (CD8) and helper (CD4) T-cell subsets (eFigure 9A-9D in Supplement 2). While CD8-positive T cells trended toward a worse response regardless of the arm, patients with granzyme B (GZMB) positive activated CD8-positive T cells benefited more from the combination (HR, 0.88; 95% CI, 0.43-1.78) compared with carboplatin alone (HR, 1.46; 95% CI, 0.72-2.93). In contrast, CD4-positive T helper cells were associated with longer PFS on both arms, whereas regulatory T cells (CD4^+^/FOXP3^+^) cells were associated with worse response (carboplatin, HR = 3.12; 95% CI, 0.92-11.0; combination, HR = 1.03; 95% CI, 0.51-2.07). Combined positive scoring (CPS) of tumor and immune PD-L1–positive cells was associated with longer PFS on both arms at CPS greater than 1, and to a lesser extent at CPS higher than 10 (Figure 5). Because antigen presentation is necessary for tumor-immune recognition, we evaluated MHC-I expression (HLA-A) in tumor epithelium. Tumor HLA-A expression ranged from 4.7%-100%, with a median (SD) of 97.9% (28.4%) (eFigure 9E in Supplement 2). Regardless of the treatment arm, low tumor-specific HLA-A expression (<80%) was associated with decreased PFS (carboplatin, HR = 1.90; 95% CI, 0.97-3.69; combination, HR = 1.56, 95% CI, 0.81-2.97) (Figure 5).

## Discussion

In this study, adding atezolizumab to carboplatin therapy increased PFS from 2.2 to 4.1 months. This increase is similar to adding pembrolizumab (5.6 to 7.5 months) to a gemcitabine-carboplatin therapy.^23^ The combination of atezolizumab and carboplatin also increased OS in IIT patients with metastatic disease from 8.6 to 12.6 months.

Patients receiving atezolizumab monotherapy after disease progression on carboplatin had similar OS with fewer toxic effects than those receiving the combination, suggesting sequential chemotherapy and immunotherapy may be another option for those patients in which clinical toxic effects management is critical. However, additional studies are needed for validation because this study was not powered for that comparison and treatment crossover may inherently select for patients with less aggressive disease.

It is unclear which biomarkers are useful for predicting immune checkpoint therapy response. Atezolizumab was initially approved for PD-L1–positive metastatic TNBC,^6^ and pembrolizumab showed benefit in patients with PD-L1–positive disease.^24^ However, benefit of atezolizumab added to paclitaxel was not observed in patients with PD-L1–positive disease in the IMpassion131 study.^5^ In this study, PD-L1 status did not affect PFS or OS with immunotherapy. However, the incidence of PD-L1–positive tumors (22%) was substantially lower than the 41%, 45%, and 38% reported in the IMpassion130,^6^ IMpassion131,^5^ and KEYNOTE-355 clinical trials,^24^ respectively. Lower PD-L1 positivity may be attributed to the proportion of biopsies evaluated from primary vs metastatic sites. In this study, most (75%) were metastatic biopsies compared with 48% in the IMpassion131 trial.^5^ Furthermore, the PD-L1 positivity rates vary by metastatic biopsy location, with liver, skin, and bone metastases displaying much lower rates than other sites.^25^ Regardless, there was a significant increase in PFS and OS in PD-L1–negative tumors consistent with the KEYNOTE-522 trial, in which pembrolizumab provided a benefit regardless of PD-L1 status.^26^

Like other studies, we observed increased survival benefit for patients receiving the combination whose tumors had higher pretreatment TILs. Multi-immunofluorescence analyses demonstrated that although CD8 T cells were associated with shorter PFS on both arms, activated (GZMB^+^) T cells were associated with improved PFS only on the combination arm. Furthermore, CD4 T cells were associated with increased PFS, while FOXP3-positive regulatory T cells were associated with decreased PFS for patients receiving the combination.

Mutation burden has been associated with immunotherapy response retrospectively^27^ and prospectively.^26^ We observed a similar association in patients with TMB-H tumors, having increased PFS on both arms. Activating *PIK3CA* mutations were the only variant associated with a lack of response to immunotherapy. However, *PIK3CA* variants were also associated with decreased clinical benefit with carboplatin alone, consistent with a prior study^28^ in which patients with *PIK3CA* variants had a lower response to anthracycline-based neoadjuvant chemotherapy.

In this study, we observed an association of obesity and uncontrolled blood glucose levels with better responses to PD-L1 immunotherapy. Obesity has been associated with increased efficacy of PD-1/PD-L1 blockade and attributed to T-cell dysfunction,^29^ and retrospective studies have shown that patients with obesity with breast cancer achieved the highest benefit.^30^ This benefit could be attributed to higher adipose tissue composition in the breast and augmented by metabolic syndrome conditions such as type 2 diabetes. Changes in blood glucose levels in fasting-mimicking diets profoundly affect peripheral blood cell composition by reducing immunosuppressive myeloid and regulatory T-cell compartments.^31^ Patients with obesity with elevated blood glucose levels may have more immunosuppressive immune cells upon which anti-PD1/PD-L1 therapies can act. Future studies are needed to validate these findings and delineate the effects of blood glucose and obesity.

### Limitations

Although preliminary associations were observed in the molecular subgroup analyses, caution should be exercised in interpretation because these were retrospective, exploratory, and limited by small patient numbers and require further investigation in larger cohorts to draw definitive conclusions.

## Conclusions

In this multicenter phase 2 randomized clinical trial, adding atezolizumab to carboplatin significantly increased PFS and OS in patients with metastatic TNBC. Increased TILs, higher TMB, obesity, and uncontrolled blood glucose levels were associated with a decreased risk of progression, whereas tumor HLA-A expression, *PIK3CA* variants, and the LAR subtype were associated with a greater risk of progression for patients receiving the combination. Crossover patients receiving sequential chemotherapy and immunotherapy had fewer toxic effects than patients receiving the combination, suggesting sequential treatment could be considered for patients whose treatment-related toxic effects are of concern.

## Notes

Supplement 1. Trial Protocol

Supplement 2. eAppendix eMethods eFigure 1. Trial Schema eFigure 2. Tumor response by treatment arm eFigure 3. PD-L1 IHC association with response eFigure 4. Intratumor and stromal tumor-infiltrating lymphocyte quantification and association with response eFigure 5. Body mass index and average blood glucose eFigure 6. RNA seq and TNBC subtyping eFigure 7. Mutational analysis of DNA-seq eFigure 8. Association of TP53, BRCA1/2 and PIK3CA mutations with survival eFigure 9. Multiplex-immunofluorescence staining of pretreatment tumor biopsies eTable 1. Demographics and tumor characteristics for patients enrolled in TBCRC 043 eTable 2. Treatment-related adverse events occurred in >5% of patients eTable 3. Clinical response and endpoints

Supplement 3. eTable 4. Participant Characteristics eTable 5. Differentially expressed gene in responding patients receving atezolizumab + carboplatin

Supplement 4. Data Sharing Statement
