# Supplementary material for: Atezolizumab in Combination With Carboplatin and Survival Outcomes in Patients With Metastatic Triple-Negative Breast Cancer: The TBCRC 043 Phase 2 Randomized Clinical Trial
Source: JAMA Oncol. 2023 Dec 14;10(2):193–201. doi: 10.1001/jamaoncol.2023.5424 (PMC10722391; doi:10.1001/jamaoncol.2023.5424)
Supplement: Supplement 1. — Trial Protocol [file jamaoncol-e235424-s001.pdf]

A Phase II trial of Atezolizumab (anti-PDL1) with Carboplatin in Patients with Metastatic Triple Negative Breast Cancer

Protocol Number GNE ML30041 / TBCRC 043
VICC BRE 15136

Protocol Chair

Vandana G. Abramson, MD (Protocol Chair)
Vanderbilt-Ingram Cancer Center
2220 Pierce Ave. 777 PRB
Nashville, TN 37232
Phone: (615) 322-4967
Fax: (615) 343-7602
E-mail: vandana.abramson@vumc.org

Jennifer Pietenpol, PhD
Vanderbilt-Ingram Cancer Center
2220 Pierce Ave. 777 PRB
Nashville, TN 37232
Phone: (615) 322-4967
Fax: (615) 343-7602
jennifer.pietenpol@vumc.org

Laboratory Correlatives:

Brian Lehmann, PhD
Vanderbilt-Ingram Cancer Center
2220 Pierce Ave. 777 PRB
Nashville, TN 37232
Phone: (615) 322-4967
Fax: (615) 343-7602
E-mail: brian.d.lehmann@vumc.org

Pathology:

Melinda Sanders, MD
Vanderbilt-Ingram Cancer Center
Department of Pathology 4912 TVC
1301 22nd Ave South
Nashville, TN 37232-5310
Phone: 615-343-9115
E-mail: melinda.sanders@vumc.org

Biostatistics:

Yu Shyr, PhD
Vanderbilt-Ingram Cancer Center
2220 Pierce Ave. 777 PRB
Nashville, TN 37232
Phone: (615) 322-4967
Fax: (615) 343-7602
E-mail: yu.shyr@vumc.org

Supporters Genentech
Johns Hopkins University on behalf of the Translational Breast Cancer Research Consortium (TBCRC)

Coordinating Center Vanderbilt-Ingram Cancer Center (VICC)

Study Liaison:
VICC-CTSR Coordinating Center
3322 West End Ave Suite 1000
Nashville, TN 37203
Phone: (615) 936-5770
Email:coordinating.center@vumc.org

VICC BRE 15136: A Phase II trial of Atezolizumab (anti-PDL1) with Carboplatin in Patients with Metastatic Triple Negative Breast Cancer

- Protocol Revision Record -

<u>Original Protocol</u>: **4/4/2016**

Protocol Revision 1: **6/22/2017**

Protocol Revision 2: **8/04/2017**

Protocol Revision 3: **11/19/2018**

Protocol Revision 4: **04/06/2020**

Protocol Revision 5: **07/30/2020**

Protocol Revision 6: **11/13/2020**

Table of Contents

1. SCHEMA 6
2. STUDY DESIGN/SUMMARY 7
3. OBJECTIVES 7
3.1 PRIMARY OBJECTIVE 7
3.2 SECONDARY OBJECTIVES 7
3.3 EXPLORATORY OBJECTIVES 7
4. BACKGROUND 8
4.1 INVESTIGATIONAL AGENTS 12
4.2 RATIONALE 18
5. PARTICIPANT SELECTION 18
5.1 INCLUSION CRITERIA 18
5.2 EXCLUSION CRITERIA 20
5.3 INCLUSION OF UNDERREPRESENTED POPULATIONS 23
6. REGISTRATION PROCEDURES 23
6.1 GUIDELINES FOR ALL PARTICIPATING INSTITUTIONS 23
6.2 BIOPSY OF METASTATIC LESION 25
7. STUDY CALENDARS 26
7.1 STUDY CALENDAR: CARBOPLATIN +/- ATEZOLIZUMAB 26
7.2 STUDY CALENDAR: ATEZOLIZUMAB (CROSSOVER) 29
8. TREATMENT PLAN 31
8.1 OVERVIEW 31
8.2 CONCOMITANT TREATMENT AND SUPPORTIVE CARE GUIDELINES 31
8.3 ASSESSMENTS DURING TREATMENT 32
8.4 CROSS-OVER POST-PROGRESSION 32
8.5 DURATION OF THERAPY 33
8.6 DURATION OF FOLLOW-UP 33
8.7 CRITERIA FOR REMOVAL FROM STUDY 33
9. EXPECTED TOXICITIES AND DOSING DELAYS/DOSE MODIFICATIONS 35
9.1 ANTICIPATED TOXICITIES OF ATEZOLIZUMAB (ATEZOLIZUMAB) AND DOSE MODIFICATIONS 35
9.2 GENERAL GUIDELINES FOR DOSAGE MODIFICATION AND TREATMENT INTERRUPTION OR DISCONTINUATION 36
9.3 ANTICIPATED TOXICITIES OF CARBOPLATIN AND DOSE MODIFICATIONS 60
9.4 SPECIAL CONSIDERATIONS 63
10. DRUG FORMULATION/STORAGE/SUPPLY 64
10.1 ATEZOLIZUMAB (MPDL3280A) 64
10.2 CARBOPLATIN 65
10.3 DRUG ACCOUNTABILITY 66
11. CORRELATIVE/SPECIAL STUDIES 67
11.1 ARCHIVED TISSUE SAMPLES 67
11.2 BLOOD/PLASMA COLLECTION 70

11.3 TISSUE SPECIMEN AND BLOOD/PLASMA LABELING AND DOCUMENTATION 70
11.4 PERIPHERAL BLOOD MONONUCLEAR CELL COLLECTION 70
11.5 GENETIC TESTING 70
11.6 SPECIMEN BANKING 71
11.7 BIOPSY QUESTIONNAIRE 71
11.8 IMAGING CORRELATIVE STUDY 71

12. MEASUREMENT OF EFFECT 71

12.1 DEFINITIONS 73
12.2 DISEASE PARAMETERS 73
12.3 RESPONSE CRITERIA 75
12.4 DURATION OF RESPONSE 77
12.5 IMMUNE-RELATED RESPONSE CRITERIA 77
12.6 RESPONSE REVIEW AND CENTRAL TUMOR MEASUREMENTS 79

13. ASSESSMENT OF SAFETY ADVERSE EVENT REPORTING 80

13.1 GENERAL 80
13.2 RISKS ASSOCIATED WITH ATEZOLIZUMAB 80
13.3 SAFETY PARAMETERS AND DEFINITIONS 81
13.4 ASSESSMENT OF ADVERSE EVENTS 82
13.5 REPORTING PROCEDURES 83
13.6 COORDINATING CENTER REPORTING PROCEDURES 91

14. DATA SAFETY AND MONITORING 97

14.1 DATA MANAGEMENT AND REPORTING 97
14.2 MEETINGS 97
14.3 MONITORING 98
14.4 DATA HANDLING AND RECORD KEEPING 98

15. REGULATORY CONSIDERATIONS 99

15.1 PROTOCOL REVIEW AND AMENDMENTS 99
15.2 INFORMED CONSENT 99
15.3 ETHICS AND GCP 100

16. MULTI-CENTER GUIDELINES 100

16.1 PROTOCOL REVIEW AND AMENDMENTS 100
16.2 STUDY DOCUMENTATION 100
16.3 RECORDS RETENTION 101
16.4 PUBLICATION 101

17. STATISTICAL CONSIDERATIONS 101

17.1 STUDY DESIGN/ ENDPOINTS 101
17.2 SAMPLE SIZE/ ACCRUAL RATE 102
17.3 INTERIM ANALYSIS 102
17.4 ANALYSIS OF SECONDARY AND EXPLORATORY ENDPOINTS 102
17.5 STATISTICAL ANALYSIS PLAN 103
17.6 REPORTING AND EXCLUSIONS 103

REFERENCES 103

1. SCHEMA

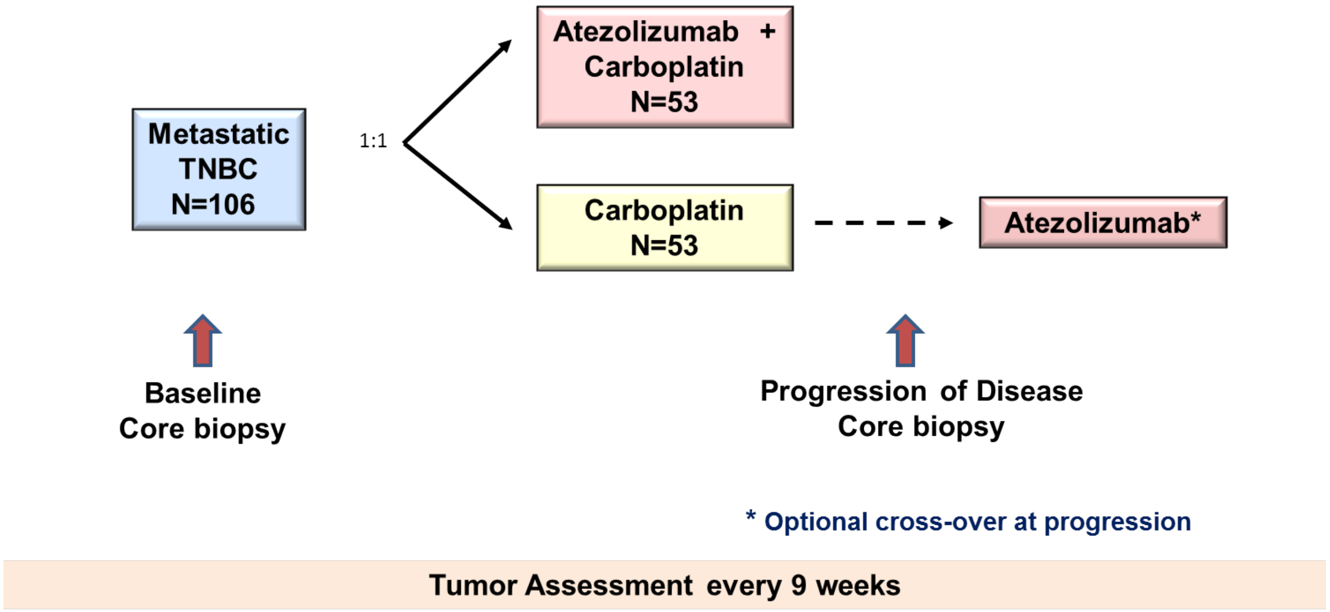
Metastatic TNBC N=106
1:1
Atezolizumab + Carboplatin N=53
Carboplatin N=53
Atezolizumab*
Baseline Core biopsy
Progression of Disease Core biopsy
* Optional cross-over at progression
Tumor Assessment every 9 weeks

2. STUDY DESIGN/SUMMARY

The primary objective of this phase II multiple institution trial is to evaluate the efficacy of carboplatin + atezolizumab versus carboplatin alone in patients with metastatic triple negative breast cancer. Patients with an ECOG performance status of 0-1 who have received either zero or one prior regimen for metastatic disease are eligible. Patients will be randomized to one of two arms. In arm 1, patients will receive combination therapy with carboplatin and atezolizumab and in arm 2 patients will receive carboplatin monotherapy. All patients will undergo core biopsy of a metastatic lesion (if reasonably safe) at baseline and at the time of progression. If a fresh biopsy is deemed not safe, a paraffin block from a prior biopsy or surgery must be available for enrollment. Standard staging radiology scans will be performed at baseline and every 9 weeks thereafter. Upon progression of disease (clinically or by RECIST), patients on the carboplatin alone arm may cross over to arm 1 to receive atezolizumab. All patients with progression of disease who want to cross over to receive atezolizumab are required to undergo a biopsy of a metastatic site at progression if reasonably safe. Patients who were receiving carboplatin + atezolizumab will be discontinued from study medications upon disease progression by RECIST. Patients will be screened and enrolled from the oncology practice at the Vanderbilt-Ingram Cancer Center and from participating member centers of the Translational Breast Cancer Research Consortium (TBCRC).

3. OBJECTIVES

3.1 Primary Objective

- To evaluate the efficacy, as measured by median progression free survival (PFS) of carboplatin + atezolizumab (using RECIST) versus carboplatin alone (using RECIST) in patients with triple negative metastatic breast cancer

3.2 Secondary Objectives

- To determine overall response rate (ORR)
- To evaluate the efficacy, as measured by clinical benefit rate (CBR), of carboplatin + atezolizumab versus carboplatin alone in patients with triple negative metastatic breast cancer. CBR is defined as complete response (CR) plus partial response (PR) plus stable disease (SD) for 6 months.
- To determine the duration of response (DOR) for patients achieving a partial or complete response
- To evaluate the overall survival (OS) of carboplatin + atezolizumab versus carboplatin alone in patients with triple negative metastatic breast cancer
- To evaluate PFS, ORR, and DOR based on irRECIST as assessed by central imaging review.

3.3 Exploratory Objectives

- To perform the following correlative studies from biopsies taken at baseline:
  a) Tumor infiltrating lymphocyte frequency and phenotype (TILs) at baseline

b) PD-L1 expression from the baseline pre-treatment tissue and at progression lesion, performed by IHC (SP142 clone) using a facility designated by the Supporter (Genentech)
c) HER2 (IHC, FISH) and ER/PR levels (IHC) from a metastatic site
d) Perform RNA-seq to determine non-synonymous mutation burden in expressed genes and gene expression to assign a triple negative subtype at baseline for correlations with clinic outcome
e) Immune phenotyping (IHC) for markers of T cell subsets and activation (CD4, CD8, FoxP3, CD25, Glut1) and exhaustion (PD1, CTLA4) and test feasibility of flow cytometric analyses to include additional markers

- To assess the effect of BRCA mutations on response to the study drugs
- To evaluate the effect of steroids on the efficacy of atezolizumab
  To assess the prognostic effects of TILs on PFS and CBR in patients receiving atezolizumab
- To determine the correlation between low muscle mass and tumor immunophenotype by PDL1 expression, TIL frequency, tumor mutation burden, T cell activation, and T cell exhaustion.
- To determine the correlation between low muscle attenuation and tumor immunophenotype by PDL1 expression, TIL frequency, tumor mutation burden, T cell activation, and T cell exhaustion.
- To determine the correlation between adipose mass and tumor immunophenotype by PDL1 expression, TIL frequency, tumor mutation burden, T cell activation, and T cell exhaustion.
- To determine the prognostic impact of LMM, LMA, and adiposity on progression free survival in patients receiving atezolizumab (anti-PDL1).

4. BACKGROUND

Over the past decade, the term "triple negative breast cancer" has been used to classify tumors that lack expression of the estrogen receptor (ER), progesterone receptor (PR), and HER2. Triple negative breast cancers (TNBC) are generally more aggressive than their ER positive counterparts, with higher rates of relapse in the early stage and decreased overall survival in the metastatic setting. TNBC is a pathologically, molecularly and clinically heterogeneous disease and the lack of recurrent targetable alterations makes this disease particularly difficult to treat. Using gene expression analyses from 386 tumors, we recently identified six distinct TNBC subtypes, each displaying unique biologies (**Figure 1**). The TNBC molecular subtypes include two basal-like (BL1 and BL2), an immunomodulatory (IM), a mesenchymal (M), a mesenchymal stem-like (MSL), and a luminal androgen receptor (LAR) subtype [1]. More importantly, the inherent differences between the subtypes predict for differential responses to standard chemotherapy. In a retrospective re-analysis of pretreatment biopsies, TNBC molecular subtypes have shown clinical utility and are predictive of response to neoadjuvant anthracycline and cyclophosphamide followed by taxane (PMID:23948975)[2]. This study showed BL1 had the highest pCR rate (50%) at time of surgery and BL2 and LAR the lowest (0 and 10%, respectively), deviating from the overall response of 32% for unselected TNBC [2]. The differing biologies of TNBC subtypes, as indicated by their gene expression signatures, are providing significant insight to differential response to therapies.

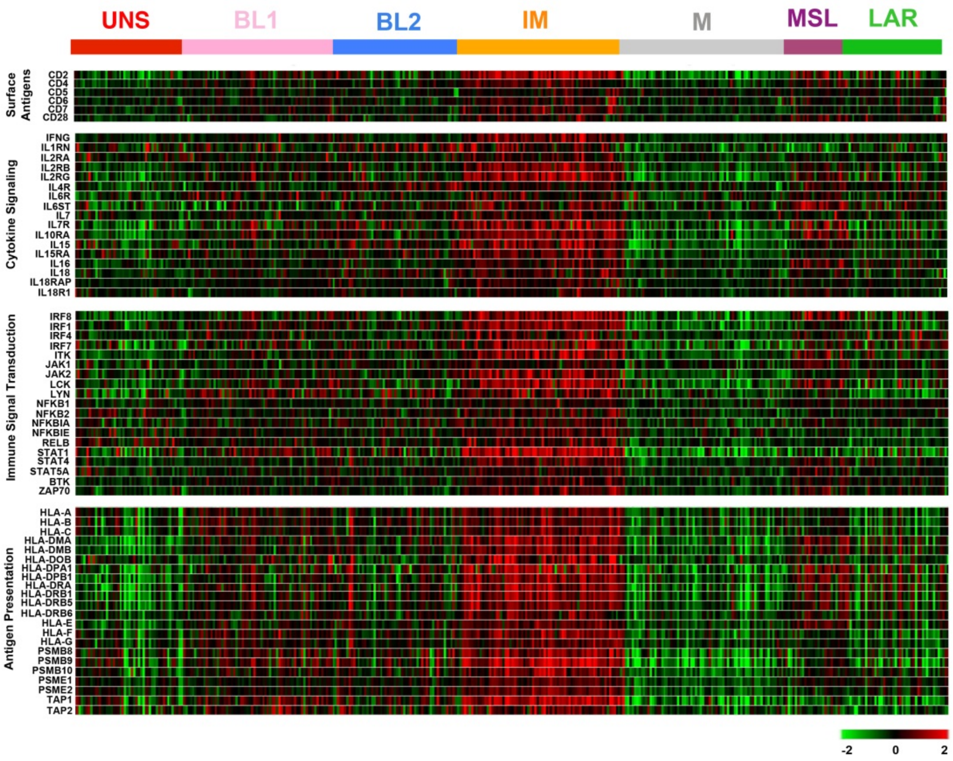

Figure 1. IM TNBC subtype is enriched in immune genes that are involved in cytokine signaling, immune signal transduction and antigen presentation. Heatmap shows relative expression of immune related genes across TNBC molecular subtypes from 386 tumor gene expression arrays.

Comparable subtyping results have been obtained from two independent analyses of gene expression arrays and each have identified similar subtypes, including a immune-activated or -associated biology [3, 4]. The IM subtype is composed of immune antigens and genes involved in cytokine and core immune signal transduction pathways (**Figure 2**). The data from our work and others suggest that the IM subtype is composed of an immune-activated and associated signaling components contributed from both the tumor and the infiltrating lymphocytes. Adams and colleagues recently reported that higher levels of tumor infiltrating lymphocytes (TILs), in evaluable TNBC tumors collected from patients enrolled in an adjuvant phase III trial, were prognostic for overall and disease-free survival [5]. Similarly, the presence of TILs in pretreatment biopsies was especially predictive of response to neoadjuvant chemotherapy in the carboplatin arm of the GeparSixto trial [6]. These preliminary data suggesting that TILs are predictive markers of response, specifically to neoadjuvant platinum agent compared to liposomal-doxorubicin, suggest that cell death induced by platinum agents may "prime" or generate neoantigens to stimulate nearby lymphocytes.

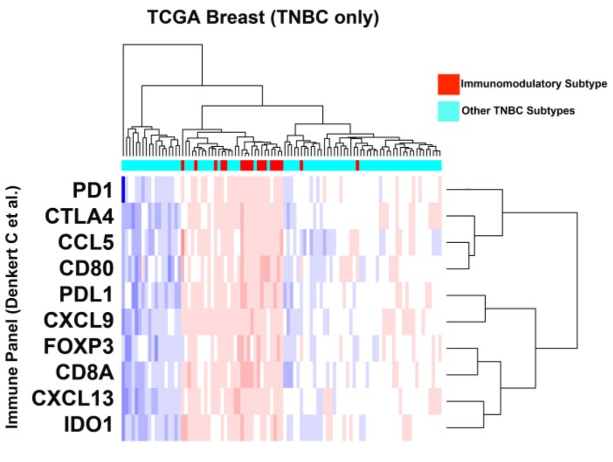

Figure 2. Immune regulatory genes that are indicative of tumor immune cell infiltrate are enriched in the IM TNBC subtype. Heatmap shows unsupervised clustering of immune panel of genes that highly correlate with pathological evaluation of tumor infiltrating lymphocytes using RNA-seq expression from TNBC tumor samples from TCGA.

Analysis of the gene expression data from the IM subtype and identification of transcripts associated with lymphocytes further supports that the IM tumor samples contain tumor-infiltrating lymphocytes (TILs). This is illustrated by observed enrichment of immune markers in the RNA-seq data from TNBC samples in The Cancer Genome Atlas (TCGA) [6]. Unsupervised hierarchical clustering showed a strong correlation of TIL-associated immune signatures in TNBC tumors of the IM subtype compared to all other TNBC tumors (Figure 3).

Given the recent efficacy of anti-PDL1 compounds in TNBC [7], we examined the levels of known immune checkpoint regulatory genes (PDL1, PD1 and CTLA4) to determine if these targets are differentially expressed in TNBC subtypes. Breast cancer RNA-seq data (RSEM) was downloaded (Broad Firehose) and TNBC samples subtyped as previously described [1, 8]. PDL1, PD1 and CTLA4 expression were significantly higher in the IM TNBC subtype (4437.4 vs. 784.1, p=0.0001; 144.3 vs. 31.4, p=0.001 and 167.8 v. 39.5, p=0.0001, respectively) compared to all other breast cancers (**Figure 3A-3C**). These data demonstrate that the IM TNBC subtype is significantly enriched in the expression of genes involved in immune checkpoint signaling. Assessment of the IM signature in relation to spatial localization of T cells may identify a subset of TNBC in which immune cells may be present and checkpoint inhibitors have efficacy.

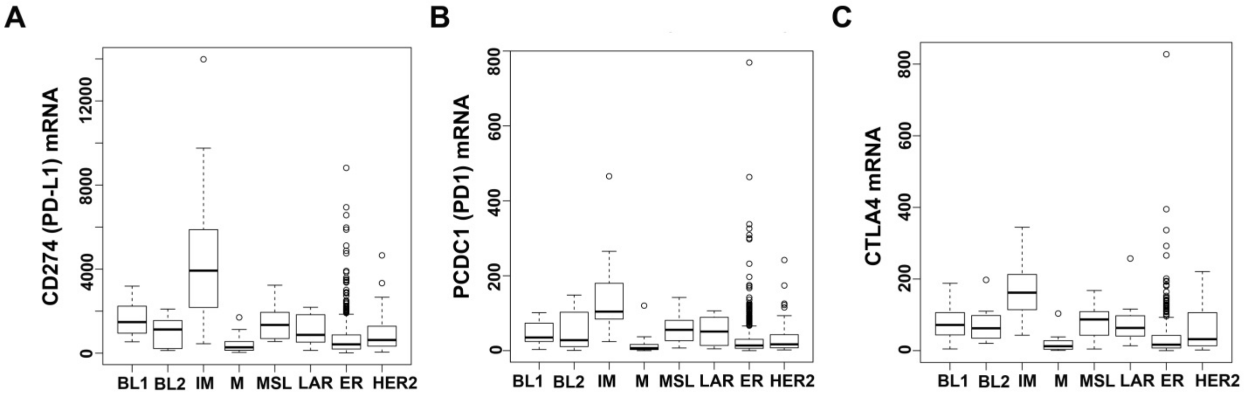

Figure 3. Expression of immune signaling components is enriched in immunomodulatory (IM) TNBC. Boxplots show mRNA expression (RSEM counts) for **(A)** PD-L1, **(B)** PD1 and **(C)** CTLA4 across TNBC molecular subtypes (BL1, BL2, IM, M, MSL and LAR) or ER-positive (ER) and HER2-amplified (HER2) breast. cancer from TGCA.

Since there has been a strong correlation between non-synonymous mutation burden and response to anti PD-1 therapy in non–small cell lung cancer (NSCLC), and anti-PD-L1 antibodies have efficacy in TNBC, we examined non-synonymous mutation burden in TNBC and non-TNBC tumors from the TCGA [9]. The number of non-synonymous mutations per sample was higher for TNBC (n=69) than non-TNBC (n=38), suggesting the possibility that TNBC tumors may be more immunogenic and express more neoantigens (**Figure 4B**). In addition, the mutational burden was differential among TNBC molecular subtypes, with significantly more amino acid coding mutations in the BL1 (138.2 vs. 70.0, p=0.0093) and M subtypes (133.9 vs. 70.0, p=0.036) compared to all other subtypes (Figure 4 B).

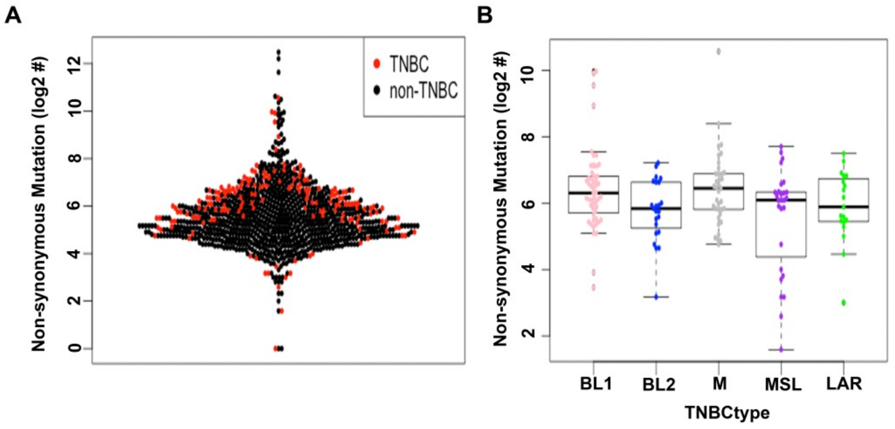

Figure 4. TNBC tumors in TCGA have a higher non-synonymous mutation burden. **(A)** Beeswarm plot shows the number of coding amino acid changing mutations in TNBC (red) and non-TNBC tumors (black). **(B)** Boxplot show mutational burden by TNBCytype

A better understanding of the clinical and pathologic factors associated with benefit to immunotherapy in metastatic breast cancer could improve personalized therapy and provide insight into mechanisms of resistance. One such factor may be the body composition of the host. Obesity is associated with systemic inflammation and suppression of adaptive immunity, yet augmented T cell responses after PD1/PDL1 blockade[11]; lower muscle mass is associated with pro-inflammatory cytokines that may promote tumor growth and impair response to immune checkpoint blockade[12,13]. Low muscle mass (LMM), or sarcopenia, is associated with poorer outcome and increased toxicity in advanced cancer[14]. Low muscle attenuation (LMA), reflecting muscle "quality" and intramuscular fat infiltration, is also associated with poor outcome and reflects physical functioning[15]. To date, there has been no evaluation of how muscle or adiposity impact immune phenotype and outcome in patients with metastatic breast cancer receiving immunotherapy. TBCRC043 offers a homogeneous population of patients with metastatic TNBC with baseline biopsies for immunophenotyping, centrally collected body imaging, and outcome follow up to investigate this question. Using transverse cuts at the third lumbar vertebrae on abdominal CT scans as a reflection of total body composition[16,17], we will quantify muscle mass, muscle attenuation, and adiposity. Low muscle mass is defined as skeletal muscle index (SMI, lean muscle area/height, cm^2/m^2) less than 41 and low muscle attenuation will be defined as average muscle density less than 25 HU, or less than 33 HU if the patient is overweight/obese by BMI[18]. Total adiposity will be defined as the sum of visceral adipose tissue, subcutaneous adipose tissue, and intramuscular adipose tissue and evaluated by tertiles. Regarding the impact of body composition on immune phenotype and prognosis in patients receiving PDL1 blockade for metastatic triple negative breast cancer in TBCRC-043, our primary hypothesis is that LMM

(sarcopenia) is associated with T cell exhaustion (PD1 and CTLA4 expression) and worse progression free survival in patients with metastatic breast cancer receiving anti-PDL1 therapy, attenuating the prognostic effect of tumor infiltrating lymphocytes.

4.1 Investigational Agents

4.1.1 Atezolizumab (Atezolizumab)

Atezolizumab is a human immunoglobulin (Ig) G1 monoclonal antibody consisting of two heavy chains (448 amino acids) and two light chains (214 amino acids) and is produced in Chinese hamster ovary cells. Atezolizumab was engineered to eliminate Fc-effector function via a single amino acid substitution (asparagine to alanine) at position 298 on the heavy chain, which results in a non-glycosylated antibody that has minimal binding to Fc receptors and prevents Fc-effector function at expected concentrations in humans. Atezolizumab targets human programmed death-ligand 1 (PD-L1) and inhibits its interaction with its receptor, programmed death-1 (PD-1). Atezolizumab also blocks the binding of PD-L1 to B7.1, an interaction that is reported to provide additional inhibitory signals to T cells.

Atezolizumab is being investigated as a potential therapy against solid tumors and hematologic malignancies in humans.

4.1.1.1 Summary of Nonclinical Experience

The nonclinical strategy of the atezolizumab program was to demonstrate in vitro and in vivo activity, to determine in vivo pharmacokinetic (PK) behavior, to demonstrate an acceptable safety profile, and to identify a Phase I starting dose. Comprehensive pharmacology, PK, and toxicology evaluations were thus undertaken with atezolizumab.

The safety, pharmacokinetics, and toxicokinetics of atezolizumab were investigated in mice and cynomolgus monkeys to support intravenous (IV) administration and to aid in projecting the appropriate starting dose in humans. Given the similar binding of atezolizumab for cynomolgus monkey and human PD-L1, the cynomolgus monkey was selected as the primary and relevant nonclinical model for understanding the safety, pharmacokinetics, and toxicokinetics of atezolizumab.

Overall, the nonclinical pharmacokinetics and toxicokinetics observed for atezolizumab supported entry into clinical studies, including providing adequate safety factors for the proposed Phase I starting doses. The results of the toxicology program were consistent with the anticipated pharmacologic activity of down-modulating the PD-L1/PD-1 pathway and supported entry into clinical trials in patients.

Refer to the atezolizumab Investigator's Brochure for details on the nonclinical studies.

4.1.1.2 Clinical Experience with atezolizumab

4.1.1.2.1 Onoging Clinical Studies

Current studies of atezolizumab include one ongoing Phase Ia monotherapy study, three ongoing combination studies, five Phase II studies, and one Phase III study. Details of all ongoing studies can be found in the atezolizumab Investigator's Brochure.

Phase Ia Study PCD4989g
Study PCD4989g is a multicenter, first-in-human, open-label, dose-escalation study evaluating the safety, tolerability, immunogenicity, pharmacokinetics, exploratory pharmacodynamics, and preliminary evidence of biologic activity of atezolizumab administered as a single agent by IV infusion every 3 weeks to patients with locally advanced or metastatic solid malignancies or hematologic malignancies. Ongoing expansion cohorts are studying the efficacy in patients with pancreatic cancer, bladder cancer, breast cancer, esophageal cancer, prostate cancer, small-cell lung cancer, malignant lymphoma, multiple myeloma, and other less common tumor types.

Phase Ib Study GP28328
Ongoing Phase Ib Study GP28328 is evaluating the safety and pharmacology of atezolizumab administered with bevacizumab alone (Arm A) or with bevacizumab plus leucovorin, 5-fluorouracil, and oxaliplatin (FOLFOX; Arm B) in patients with advanced solid tumors. Additional cohorts have been included to investigate atezolizumab in combination with carboplatin plus paclitaxel, in combination with carboplatin plus pemetrexed, and in combination with carboplatin plus nab paclitaxel, pemetrexed, and cisplatin in patients with advanced or metastatic non-small cell lung cancer (NSCLC).

Phase Ib Study GP28384
Ongoing Phase Ib Study GP28384 is evaluating the safety and pharmacology of atezolizumab administered in combination with vemurafenib in patients with previously untreated $BRAF^{V600}$-mutation–positive metastatic melanoma.

Phase Ib Study GP28363
Ongoing Phase Ib Study GP28363 is evaluating the safety and pharmacology of atezolizumab administered in combination with cobimetinib (MEK inhibitor) in locally advanced or metastatic solid tumors.

Phase II Study GO28625 (FIR)
Ongoing, single-arm, Phase II Study GO28625 is evaluating the safety and efficacy of atezolizumab monotherapy in PD-L1–positive patients with NSCLC. In particular, this study is evaluating whether archival or fresh tumor tissue is more predictive of response to atezolizumab. Safety and efficacy data are not yet available for this study.

Phase II Study GO28753 (POPLAR)
Study GO28753 is a randomized, open-label, phase II study in patients with locally advanced or metastatic NSCLC who have failed a prior platinum-containing regimen. Patients in the control arm of Study GO28753 will receive docetaxel alone. Eligible patients will be enrolled regardless of PD-L1 status and will be stratified by PD-L1 expression. The primary endpoint is overall survival (OS) for both the PD-L1–positive population and the overall study population.

Phase II Study GO28754 (BIRCH)

Ongoing, single-arm, Phase II Study GO28754 is evaluating the safety and efficacy of atezolizumab monotherapy in PD-L1−positive patients with NSCLC. Safety and efficacy data are not yet available for this study.

Phase II Study WO29074
Ongoing Phase II Study WO29074 is evaluating the safety and efficacy of atezolizumab monotherapy or the combination of atezolizumab and bevacizumab versus sunitinib in treatment-naïve patients with renal cell carcinoma (RCC). As of the clinical cutoff date (CCOD) of 17 October 2016, safety data was available from 304 patients in the safety-evaluable population, of whom 101 were treated with ATZ+BEV, 103 with atezolizumab alone, and 100 with sunitinib. The safety of atezolizumab in combination with bevacizumab was consistent with the known safety profile of individual treatment components; no new safety signals were identified. Safety of atezolizumab monotherapy was also consistent with the known profile of atezolizumab. The safety data demonstrated a more favorable safety profile for atezolizumab monotherapy compared to sunitinib. No new safety signals were identified.

Phase II Study GO29293
Ongoing Study GO29293 is a single-arm, open label, Phase II study to assess the clinical benefit of atezolizumab as a single agent in patients with locally advanced or metastatic UBC. The co-primary endpoints of this study are independent review facility (IRF)−assessed objective response rate (ORR) according to Response Evaluation Criteria in Solid Tumors, Version 1.1 (RECIST v1.1) and investigator-assessed ORR according to modified Response Evaluation Criteria in Solid Tumors (RECIST) criteria.

Phase III Study GO28915 (OAK)
Study GO28915 is a randomized, open-label, Phase III study in patients with locally advanced or metastatic NSCLC who have failed a prior platinum-containing regimen. Patients in the control arm of Study GO28915 will receive docetaxel alone. Eligible patients will be enrolled regardless of PD-L1 status and will be stratified by PD-L1 expression. The primary endpoint is OS for both the PD-L1−positive population and the overall study population.

For further details, see the atezolizumab Investigator's Brochure.

4.1.1.2.2 Clinical Safety

As of the clinical cutoff date (CCOD) of 17 May 2019 > 21,000 patients with solid tumors or hematologic malignancies have received atezolizumab as a single agent or in combination with cytotoxic chemotherapy and/or targeted therapy through participation in a clinical trial.

Safety findings of single-agent atezolizumab across multiple tumor types in the clinical development program are consistent with the known mechanism of action of atezolizumab and the underlying disease. Overall, treatment with atezolizumab is well tolerated with a manageable adverse event profile. Currently, no maximum tolerated dose, no dose-limiting toxicities (DLTs), and no clear dose-related trends in the incidence of adverse events (AEs) have been determined. Among 3178 patients treated with single-agent atezolizumab for whom pooled safety data are available, the most commonly reported AEs ($\geq$ 10%) include fatigue, decreased appetite, nausea,

cough, dyspnea, constipation, pyrexia, diarrhea, anemia, back pain, vomiting, asthenia, arthralgia, pruritus, rash, headache, urinary tract infection, and peripheral edema.

The AEs observed with atezolizumab in combination with chemotherapy and/or targeted therapies are consistent with the known risks of the individual study treatment.

Atezolizumab-related AEs were comparable between patients who received atezolizumab monotherapy and those who were treated with atezolizumab in combination with targeted therapy and/or chemotherapy.

Adverse Events
The following adverse events are considered known risks of atezolizumab treatment:

**Very Common** (occurs in > 10% of patients)	• Fatigue<br>• Arthralgia<br>• Asthenia<br>• Decreased appetite<br>• Diarrhea<br>• Dyspnea<br>• Urinary tract infection<br>• Cough	• Pruritus<br>• Nausea<br>• Fever<br>• Rash<br>• Vomiting<br>• Musculoskeletal pain
**Common** (occurs in 1% - 10% of patients)	• Chills<br>• Dysphagia<br>• Increase in liver enzymes<br>• Hypersensitivity<br>• Hypokalemia<br>• Hyponatremia<br>• Hypotension<br>• Hypothyroidism<br>• Nasal congestion	• Colitis<br>• Hypoxia<br>• Flu-like symptoms<br>• Infusion-related reaction<br>• Pneumonitis<br>• Thrombocytopenia<br>• Hepatitis<br>• Abdominal pain
**Less Common but important** (occurs in < 1% of patients)	• Adrenal insufficiency<br>• Diabetes<br>• Hyperthyroidism<br>• Meningoencephalitis<br>• Hypophysitis<br>• Myocarditis<br>• Nephritis	• Guillain-Barré syndrome<br>• Myasthenic syndrome / myasthenia gravis<br>• Pancreatitis<br>• Increase in amylase and lipase<br>• Diabetic ketoacidosis<br>• Myositis

		• Severe Cutaneous Adverse Reactions (SCARs)(erythema<br>• multiforme, acute generalised exanthematous pustulosis, Stevens-Johnson syndrome<br>• (SJS), Toxic Epidermal Necrolysis (TEN) and drug rash with eosinophilia and systemic<br>• symptoms (DRESS).

Immune-Mediated Adverse Events

Immune-mediated AEs are consistent with the role of the PD-L1/PD-1 pathway in regulating peripheral tolerance. Given the mechanism of action of atezolizumab, events associated with inflammation and/or immune-mediated AEs have been closely monitored during the atezolizumab clinical program. As of investigator's brochure document date 15 July 2019 (clinical cutoff date 17 May 2019), based on pooled data from 3178 monotherapy patients with multiple tumor types, with supporting data from the estimated cumulative exposure in > 21,000 patients across all clinical trials, the overall immune-mediated adverse drug reaction (ADR) monotherapy rate for atezolizumab is 12.7%, the majority of which were Grade 1- 2 immune-mediated ADRs. In addition to infusion-related reactions, expected immune-mediated adverse drug reactions associated with atezolizumab include pneumonitis, hepatitis, colitis, pancreatitis, diabetes mellitus, hypothyroidism, hyperthyroidism, adrenal insufficiency, hypophysitis, Guillain-Barré syndrome, myasthenic syndrome/myasthenia gravis, meningoencephalitis, myocarditis, nephritis, and myositis, severe cutaneous adverse reactions (erythema multiforme, acute generalised exanthematous pustulosis, Stevens-Johnson syndrome (SJS), Toxic Epidermal Necrolysis (TEN) and drug rash with eosinophilia and systemic symptoms (DRESS).

For further details, see the atezolizumab Investigator's Brochure.

4.1.1.2.3 Clinical Activity

As of the clinical cutoff date (CCOD) 17 May 2019, efficacy data are most extensive for patients with non-small cell lung cancer (NSCLC; 1636 atezolizumab treated patients enrolled in BIRCH, POPLAR, OAK, IMpower150, IMpower131, GO28625, and the NSCLC cohort of PCD4989g) and patients with metastatic urothelial carcinoma (mUC; 983 atezolizumab-treated efficacy-evaluable patients in studies PCD4989g, IMvigor210, and IMvigor211) who were administered atezolizumab.

Results from studies evaluating atezolizumab as monotherapy in patients with locally advanced or metastatic NSCLC (PCD4989g, FIR, OAK, POPLAR, and BIRCH) single-agent treatment with atezolizumab indicated clinically meaningful overall survival (OS) improvement in the 2L/3L NSCLC intent to treat (ITT) population, in comparison with

standard of care, in both non-squamous and squamous histologies, and across all PD-L1 expression subgroups.

Atezolizumab in combination with chemotherapy and other therapeutic agents was demonstrated to have benefit in the settings of small cell lung cancer (SCLC; IMpower133) and NSCLC (IMpower132).

Clinical benefit was observed in patients with renal cell carcinoma (RCC) with the combination of atezolizumab and bevacizumab in IMmotion151. Additionally, in IMmotion150, a clinically meaningful improvement in IRC-assessed progression-free survival (PFS) was observed with atezolizumab and bevacizumab compared with sunitinib in the IC1/2/3 population, and no difference in treatment effect was observed with atezolizumab monotherapy compared with sunitinib in either the IC1/2/3 or ITT populations.

The study IMpassion130 (WO29522) demonstrated improved OS and PFS in patients with triple-negative breast cancer (TNBC) who were treated with atezolizumab and nabpaclitaxel. Updated PFS analysis results were consistent with the primary analysis, confirming long-term benefit. However, study GO30182 (IMblaze370) did not meet its objectives of improved in OS or PFS versus regorafenib in patients with colorectal cancer (CRC) who were treated with atezolizumab and cobimetinib.

Other available efficacy data suggested that treatment with atezolizumab as a single agent or in combination with other therapeutic agents resulted in anti-tumor activity across a range of other tumor types and hematologic malignancies (including pediatric-type tumors), across lines of therapy, and across PD-L1 expression subgroups.

For further details, see the atezolizumab Investigator's Brochure.

4.1.1.2.4 Clinical Pharmacokinetics and Immunogenicity

On the basis of available preliminary PK data (0.03−20 mg/kg), atezolizumab appeared to show linear pharmacokinetics at doses ≥ 1 mg/kg. For the 1-mg/kg and 20-mg/kg dose groups, the mean clearance (CL) and the mean volume at steady state (V_{ss}) had a range of 3.20−4.43 mL/kg and 48.1−64.1 mL/kg, respectively, which is consistent with the expected profile of an IgG1 antibody in humans.

The development of ATAs has been observed in patients in all dose cohorts and was associated with changes in pharmacokinetics for some patients in the lower dose cohorts (0.3, 1, and 3 mg/kg). The development of detectable ATAs has not had a significant impact on pharmacokinetics for doses from 10−20 mg/kg. Patients dosed at the 10-, 15-, and 20-mg/kg dose levels have maintained the expected target trough levels of drug despite the detection of ATAs. To date, no clear relationship between detection of ATAs and AEs or IRRs has been observed.

4.2 Rationale

Therapies for breast cancer should be guided by biologic features of the tumor, such as expression of steroid hormone receptors and hormone dependence and/or HER2/neu overexpression. Notable examples are the successful use of hormonal therapy (e.g. tamoxifen or aromatase inhibitors) for patients with hormone-sensitive (estrogen receptor [ER] and/or progesterone receptor [PR] expressing) tumors[32], and the use of trastuzumab (Herceptin™), a monoclonal antibody against HER2/neu, for patients with overexpression of the aforementioned protein[33]. These are excellent examples of where appropriate patient selection is instrumental on the success of these therapies. An issue with triple negative breast cancer is that the key drivers of this disease are not well understood, and thus molecular targets for therapeutic intervention have not been identified.

We have identified distinct subtypes of triple negative breast cancer to better understand potential drivers of disease progression and to help identify potential therapeutic targets. The presence of immune cells and expression of immune checkpoint genes in the IM subtype may identify patient populations that benefit from immune checkpoint inhibitors. In addition, carboplatin may induce an increase in mutational burden, which may increase response to immunotherapy. Considering all of our preclinical data, in this study patients with metastatic TNBC will be randomized to carboplatin with or without atezolizumab. We hypothesize that the combination of carboplatin and atezolizumab will be superior to carboplatin alone in efficacy, as measured by median progression free survival. We anticipate that high levels of pretreatment TILs or the IM subtype will correlate with better outcomes. In addition we hypothesize that the mutational burden in non-synonymous coding mutations will be predictive of response to anti-PD-L1 therapy.

5. PARTICIPANT SELECTION

5.1 Inclusion Criteria

- Patients must provide informed written consent.
- Patients must be ≥18 years of age.
- ECOG performance status 0-1.
- Clinical stage IV (metastatic) ER, PR, HER2 negative invasive mammary carcinoma, previously documented by histological analysis and that meets the following criteria:
    - HER2 negativity is defined as any of the following by local laboratory assessment: In-situ hybridization (ISH) non-amplified (ratio of HER2 to CEP17 < 2.0 or single probe average HER2 gene copy number < 4 signals/cell), or IHC 0 or IHC 1+ (if more than one test result is available and not all results meet the inclusion criterion definition, all results should be discussed with the Protocol Chair to establish eligibility of the patient)

- ER and PR negativity are defined as ≤ 10% of cells expressing hormonal receptors via IHC analysis.

- Willing to undergo biopsy of a metastatic lesion (in patients with reasonably accessible metastatic lesions such aschest wall, skin, subcutaneous tissue, lymph nodes, bones, peripheral lung, and liver metastases).

- Measurable disease, defined as at least one lesion that can be accurately measured in at least one dimension by RECIST criteria v1.1.

- Zero or one prior chemotherapy regimens for metastatic disease.

- No prior treatment with carboplatin in the metastatic setting. Carboplatin in neoadjuvant/adjuvant setting may be allowed if prior treatment with carboplatin was completed at least one year prior to initiation of this study and after discussion with the study chair.

- Patients must have adequate hematologic, hepatic, and renal function. All tests must be obtained less than 28 days from initiation of study drug (C1D1). This includes:
  - ANC ≥1000/mm^3
  - Platelet count ≥100,000/mm^3
  - Calculated creatinine clearance ≥ 30 mL/min using the Cockcroft and Gault Formula (see Section 10.2)
  - Bilirubin, SGOT, SGPT, alkaline phosphatase ≤ 2.5x upper limits of normal if no liver metastases present. Serum total bilirubin must be ≤ 3x upper limits of normal for patients with Gilbert disease. Total bilirubin, SGOT, SGPT ≤ 5x upper limits of normal if liver metastases present.

- For patients who are not postmenopausal (women) or surgically sterile (absence of ovaries and/or uterus or vasectomy), agreement to remain abstinent or to use two adequate methods of contraception (e.g., condoms, diaphragm, vasectomy/vasectomized partner, tubal ligation), during the treatment period and for at least 30 days after the last dose of study treatment. Hormone based oral contraceptives are not allowed on study. Postmenopausal is defined as:

  - Age ≥ 55 years
  - Age ≤ 55 years and amenorrheic for 12 months in the absence of chemotherapy, tamoxifen, toremifene, or ovarian suppression; or follicle stimulating hormone and estradiol in the postmenopausal range

- Subjects must complete all baseline screening assessments. The biopsy may be obtained before or after the patient is deemed eligible by administrative review, but must be before administration of study drugs on cycle 1, day 1.

5.2 Exclusion Criteria

Patients who meet any of the following criteria will be excluded from registration:

Cancer-Specific Exclusion Criteria

- Spinal cord compression not definitively treated with surgery and/or radiation, or previously diagnosed and treated spinal cord compression without evidence that disease has been clinically stable for > 2 weeks prior to randomization

- Known CNS disease, except for treated asymptomatic CNS metastases, provided all of the following criteria are met: measurable disease outside the CNS, only supratentorial and cerebellar metastases are allowed (i.e., no metastases to midbrain, pons, medulla, or spinal cord), no evidence of progression or hemorrhage after completion of CNS-directed therapy, no ongoing requirement for dexamethasone as therapy for CNS disease (anticonvulsants at a stable dose are allowed), no stereotactic radiation within 7 days or whole-brain radiation within 14 days prior to randomization.

- Leptomeningeal disease

- Uncontrolled tumor-related pain: patients requiring narcotic pain medication must be on a stable regimen at registration. Symptomatic lesions (e.g., bone metastases or metastases causing nerve impingement) amenable to palliative radiotherapy should be treated prior to randomization. Patients should be recovered from the effects of radiation. There is no required minimum recovery period. Asymptomatic metastatic lesions whose further growth would likely cause functional deficits or intractable pain (e.g., epidural metastasis that is not presently associated with spinal cord compression) should be considered for loco-regional therapy if appropriate prior to randomization.

- Uncontrolled hypercalcemia (> 1.5 mmol/L ionized calcium or calcium > 12 mg/dL or corrected serum calcium > ULN) or symptomatic hypercalcemia requiring continued use of bisphosphonate therapy. Patients who are receiving denosumab must discontinue denosumab use and replace it with a bisphosphonate instead while on study. Patients receiving a bisphosphonate for skeletal metastases are not excluded and can continue treatment.

- Malignancies other than TNBC within 5 years prior to randomization, with the exception of those with a negligible risk of metastasis or death and treated with expected curative outcome (such as adequately treated carcinoma in situ of the cervix or basal or squamous cell skin cancer).

- Concurrent anti-cancer therapy (chemotherapy, radiation therapy, surgery, immunotherapy, hormonal therapy, biological therapy) other than the ones specified

in the protocol. Any standard or investigational drugs should be discontinued 2 weeks prior to the first dose of study medication.

General Medical Exclusion Criteria

- Women only: pregnancy or lactation
- Evidence of significant uncontrolled concomitant disease that in the opinion of the investigator could affect compliance with the protocol or interpretation of results, including significant liver disease (such as cirrhosis, uncontrolled major seizure disorder, or superior vena cava syndrome)
- Significant cardiovascular disease, such as New York Heart Association (NYHA) cardiac disease (Class II or greater), myocardial infarction within 3 months prior to randomization, unstable arrhythmias, or unstable angina. Patients with a known left ventricular ejection fraction (LVEF) < 40% will be excluded. Patients with known coronary artery disease, congestive heart failure not meeting the above criteria, or LVEF < 50% must be on a stable medical regimen that is optimized in the opinion of the treating physician, in consultation with a cardiologist if appropriate.
- Severe infection requiring systemic treatment within 4 weeks prior to randomization, including but not limited to hospitalization for complications of infection, bacteremia, or severe pneumonia
- Major surgical procedure within 4 weeks prior to randomization or anticipation of the need for a major surgical procedure during the course of the study other than for diagnosis. Placement of central venous access catheter(s) (e.g., port or similar) is not considered a major surgical procedure and is therefore permitted.
- History of severe reactions (e.g. allergic, anaphylactic, or other hypersensitivity) to chimeric or humanized antibodies or fusion proteins
- Known hypersensitivity or allergy to biopharmaceuticals produced in Chinese hamster ovary cells or any component of the atezolizumab formulation
- History of autoimmune disease, including but not limited to myasthenia gravis, myositis, autoimmune hepatitis, systemic lupus erythematosus, rheumatoid arthritis, inflammatory bowel disease, vascular thrombosis associated with antiphospholipid syndrome, Wegener's granulomatosis, Sjögren's syndrome, Guillain-Barré syndrome, multiple sclerosis, vasculitis, or glomerulonephritis. Patients with a history of autoimmune-related hypothyroidism on a stable dose of thyroid replacement hormone are eligible for this study. Patients with controlled Type 1 diabetes mellitus on a stable insulin regimen are eligible for this study.

- Prior allogeneic stem cell or solid organ transplantation

- History of idiopathic pulmonary fibrosis (including pneumonitis), drug-induced pneumonitis, organizing pneumonia (i.e., bronchiolitis obliterans, cryptogenic organizing pneumonia), or evidence of active pneumonitis on screening chest CT scan. History of radiation pneumonitis in the radiation field (fibrosis) is permitted.

- Positive test for HIV (testing required prior to registration)

- Active hepatitis B (defined as having a positive hepatitis B surface antigen [HBsAg] test at screening) or hepatitis C

- Patients with past hepatitis B virus (HBV) infection or resolved HBV infection (defined as having a negative HBsAg test and a positive antibody to hepatitis B core antigen [anti-HBc] antibody test) are eligible.

- Patients positive for hepatitis C virus (HCV) antibody are eligible only if polymerase chain reaction (PCR) is negative for HCV RNA.

- Active tuberculosis

- Receipt of a live, attenuated vaccine within 4 weeks prior to initiation of study drug (C1D1) or anticipation that such a live, attenuated vaccine will be required during the study

- Prior treatment with CD137 agonists, anti−PD-1, or anti−PD-L1 therapeutic antibody or pathway-targeting agents

- Treatment with systemic immunostimulatory agents (including but not limited to interferons or IL-2) within 4 weeks or five half-lives of the drug (whichever is shorter) prior to randomization

- Treatment with systemic corticosteroids or other systemic immunosuppressive medications (including but not limited to prednisone, dexamethasone, cyclophosphamide, azathioprine, methotrexate, thalidomide, and anti−tumor necrosis factor [TNF] agents) within 2 weeks prior to cycle 1, day 1, or anticipated requirement for systemic immunosuppressive medications during the trial. Patients who have received acute, low-dose, systemic immunosuppressant medications (e.g., a one-time dose of dexamethasone for nausea) may be enrolled in the study. Patients on a stable low dose (≤ 10 mg) daily prednisone or equivalent are allowed on study. Patients with a history of allergic reaction to IV contrast requiring steroid pre-treatment should have baseline and subsequent tumor assessments performed using MRI. The use of inhaled corticosteroids for chronic obstructive pulmonary disease, mineralocorticoids (e.g., fludrocortisone) for patients with orthostatic hypotension, and low-dose supplemental corticosteroids for adrenocortical insufficiency are

allowed.

- Psychiatric illness/social situations that would compromise patient safety or limit compliance with study requirements

5.3 Inclusion of Underrepresented Populations

Individuals of all races and ethnic groups are eligible for this trial. There is no bias towards age or race in the clinical trial outlined. This trial is open to the accrual of men and women.

6. REGISTRATION PROCEDURES

All patients MUST be registered with the Vanderbilt-Ingram Cancer Center (VICC) prior to the start of protocol treatment. Each participating site must also be registered with their own institution according to their institutional guidelines prior to start of protocol treatment.

6.1 Guidelines for all participating institutions

Prior to registration, a copy of the IRB approval at the site will be requested and kept on file at the Vanderbilt-Ingram Cancer Center (VICC) Coordinating Center. Eligible participants will be entered on study centrally at the VICC Coordinating Center. All sites should email the Coordinating Center at Coordinating.Center@vumc.org to verify slot availability prior to enrollment.

All patients MUST be registered with the VICC prior to the start of protocol treatment. Registration can only be conducted during the business hours of 8AM – 5PM Central Standard Time Monday through Friday.

1) All sites must email the VICC CTSR Coordinating Center at Coordinating.Center@vumc.org to notify of upcoming registration and ensure slot availability. The following information should be included in your email:
    - Study number
    - Patient initials
    - Disease type
    - Anticipated consent date
    - Anticipated start date
2) To request a subject ID number, the site must submit the following documents with their email notification to the Coordinating Center:
    - Copy of the patient's signed and dated Informed Consent including documentation of the consent process.
    - HIPAA authorization form (if separate from the main consent form)

- VICC Patient Enrollment Form

The Coordinating Center will then provide a subject ID number via email.

3) Once the patient has been deemed eligible by the site, an email with the following documents must be sent to the Coordinating Center for eligibility review and patient enrollment (**coordinating.center@vumc.org**):

   - Copy of the patient's signed and dated Informed Consent, including documentation of the consent process.
   - HIPAA authorization form (if separate from the main consent form)
   - VICC Patient Enrollment Form
   - Eligibility supporting documents such as pathology reports, laboratory tests, etc. *or* EMR access. Note: all source documents should be de-identified and screening/subject ID number added prior to sending.
   - Tissue Block Registration Form (see the **Lab Manual**)
   - Signed and completed Eligibility Checklist. **To be eligible for registration to the study, the participant must meet each inclusion and exclusion criterion listed in the eligibility checklist.**

Note: All study documents should be received 24-48 hours prior to the patient's anticipated start date. Same day treatment registrations will only be accepted with prior notice and discussion with the Coordinating Center. Please email the Coordinating Center if enrollment is needed sooner.

Upon satisfactory review of eligibility documents submitted, the Coordinating Center will approve enrollment and issue a subject ID number if one was not issued at screening. Once registration/enrollment confirmation from Coordinating Center is received, the site may proceed with protocol procedures.

Please contact the assigned Study Contact with any questions regarding this process. You can also reach out to your assigned CRA once the study is activated.

The VICC Coordinating Center will assign sequence numbers to all patients in screening. Only patients deemed eligible will be randomized to a treatment arm. Sequence numbers will only be re-used if a patient screen fails and then re-screens. The Coordinating Center will randomize eligible patients to carboplatin + atezolizumab or carboplatin alone. Following registration and randomization, eligible participants should begin protocol treatment within 1 week. Issues that would cause treatment delays should be discussed with the Protocol Chair. If a participant does not receive protocol therapy following randomization within allowed time period, the participant will become ineligible and will not be enrolled on study. Such patients will have to undergo screening again to participate in the study in the future. Any requests for eligibility exceptions and/or deviations must be approved in writing by the Protocol Chair, as well as any committees required by local

institutional policies. As is generally accepted, standard of care procedures performed prior to consent, but within the protocol defined screening window for each assessment can be used for study purposes. All research only procedures must be performed after the consent date.

6.2 Biopsy of metastatic lesion

After signing informed consent for the research biopsy, potential participants should have a biopsy of a metastatic lesion as per Section 11 below. Tissue from the metastatic biopsy needs to be available, but not required to have been evaluated centrally, prior to beginning treatment on study.

Outside sites should ship specimens along with the Tissue Registration Form (located in the lab manual) directly to:

For all tissue and blood/plasma:
Dr. Jennifer A Pietenpol
C/o Kim Johnson
Vanderbilt University Medical Center
Department of Biochemistry
652 PRB
2220 Pierce Ave
Nashville, TN 37232
615-936-3990

Ship ALL paraffin material, RNAlater tubes or slides as detailed in the lab manual.
****Specimens should be mailed to arrive at VUMC from Monday 8AM through Friday 1PM****

7. STUDY CALENDARS

7.1 STUDY CALENDAR: CARBOPLATIN +/- ATEZOLIZUMAB

Procedure		Screening Day -28 to Day 1[b]	Cycle 1 (21-days) Day 1[a,b]	Additional cycles[a] Day 1	End-of-treatment[d]	30 day (+/- 3 days) follow-up[d]	1, 2 & 3 year follow-up[n]
Informed consent		X					
Inclusion/exclusion criteria		X					
Demographics/ medical history (SOC)		X					
Physical exam/signs/symptoms[e] (SOC)		X	X	X	X		
Vital signs (including oxygen sat)[c] (SOC)		X	X	X	X		
Single 12-lead ECG		X					
ECOG Performance Status (SOC)		X	X	X	X		
Blood collection	Clinical laboratory tests[f] (SOC)	X	X	X	X		
	Serum pregnancy test (SOC)[f]	X					
	TSH, free T3, T4[g] (After screening, atezolizumab arm only)	X		X (every 4th cycle)	X		
	PT (or INR) and PTT	X					
	HIV, HBV, HCV serology[h]	X					
	Circulating tumor DNA		X	X (Cycle 2 only)	X		
	Mononuclear Cell Collection		X	X (Cycle 2 only)	X		
Tumor assessment CTs, bone scan[i] (SOC)		X		X (every 3 cycles)			
Archived tumor blocks/slides[j]		X					
Tumor biopsy (metastatic site)[k]		X			X		
Carboplatin administration (IV) (SOC)			X	X[l]			
Atezolizumab administration (IV): if on atezolizumab arm			X	X			
Concomitant medications[m]		X	X	X			
Adverse events		X	X	X		X	X[n]
Biopsy questionnaire[o]				X (cycle 2, day 1)			
BRCA 1 and 2 testing[p] (SOC)		X					

AE = adverse event; **BSA** = body surface area; **CX** = Cycle X, where X is the cycle number; **DLT** = dose-limiting toxicity; **ECG** = electrocardiogram; **ECOG** = Eastern Cooperative Oncology Group; **HbA1C** = glycosylated hemoglobin; **IMP** = investigational medicinal product; **INR** = international normalized ratio; **PT** = prothrombin time; **PTT** = partial prothrombin time; **SAE** = serious adverse event **SOC** = Standard of Care; all tests not indicated as SOC are research-related.

a Evaluation assessments should be performed prior to administration of IMP unless otherwise indicated. Results should be reviewed by the Investigator prior to the administration of the next dose. **All visits must occur within ± 3 days from the scheduled date unless otherwise noted**. Delays up to 7 days will be considered by Study Chair.

b Screening must occur within 28days of dosing. The following procedures must be repeated at the day 1 visit if they were previously done >7 days prior to study treatment administration: Medical and cancer history, physical examination, weight measurement, vital signs, hematology, serum chemistry panel. Tumor assessments must be repeated at the baseline visit only if determined to be changed by clinical exam or other clinical observations. SOC procedures performed prior to consent but within the protocol defined screening window for each assessment can be used for study purposes. All research only procedures must be performed after the consent date.

c See section 10.1.2 for vital sign information during atezolizumab infusion.

d The 30-day follow-up assessments should be done 30 (±3 days) days after the last dose of study drugs. 30 day follow-up can be done by phone or by a clinic visit. Any ongoing toxicities should be documented. Any ongoing serious adverse events will be followed every 3 months (in person or by phone) until resolution. Any AEs or SAEs considered possibly or probably related to IMP that have not stabilized, returned to baseline, or are considered irreversible will continue to be monitored as per the protocol .

e The physical examination will include measurements of height and weight at screening. Weight will be measured on day 1 of each cycle and at the End-of-treatment visit. Dosing changes are only required if the weight changes 10% or more. Physical examination will include examination of major body systems including neurologic, cardiac, respiratory, gastrointestinal, and skin. Clinically relevant signs and symptoms will be reported as AEs.

f Clinical laboratory tests will include hematology and serum chemistry panel including white blood cell count, differential, hemoglobin, platelet count, sodium, potassium, chloride, bicarbonate, BUN, creatinine, glucose, calcium, and magnesium, AST (SGOT), ALT (SGPT), and total serum bilirubin, albumin, and total protein. Additional tests will be performed when clinically appropriate. In case of Grade 3 or higher AST/ALT, additional testing will be done weekly until recovery to baseline value (see protocol section 9.1.2 and 9.2.2.1). Labs do not have to be repeated on cycle 1, day 1 if they were done up to 7 days prior to day 1. For all other timepoints, labs can be performed up to 3 days prior to each timepoint indicated in the study calendar. **Results from labs obtained on C1D1 (up to -7 days) must meet eligibility criteria. At the start of each cycle, the ANC must be ≥1000/mm^3 and the platelet count must be ≥ 80,000/mm^3 (see section 9.2.1) for carboplatin to be administered. Atezolizumab may be administered if these criteria are not met at the discretion of the treating physician. Serum pregnancy test does not need to be performed for patients ≥ 55 years old, those who are known to be post-menopausal, or who have had a hysterectomy.**

g Thyroid function studies: see section 9.1.4 for abnormal results

h All patients will be tested for HIV and HIV-positive patients will be excluded from the clinical trial. Please note that this result must be back by the time patient starts study so it is advised this be drawn prior to day 1 if the result may take some time to obtain. HBV serology includes Hepatitis B surface antigen, anti-HBc, and anti-HBs. As above, it is advised the HBV serology be obtained in advance if results may be delayed. Hepatitis B virus (HBV) DNA must be collected on or before Cycle 1, Day 1 in patients who have negative serology for hepatitis B surface antigen and positive serology for anti-HBc.

i Tumor assessment should be performed approximately every 3 cycles (up to -5 days) while on study treatment (i.e. on or up to 5 days prior to Day 1 of Cycles 4, 7, 10 etc) and at End-of-treatment visit if not obtained within the past 6 weeks. Scans should consist of CT scan of the chest, abdomen, and pelvis and a bone scan at baseline. Of note, if the prior bone scan did not show metastases, it does not need to be repeated if the bone scan was obtained within 8 weeks of C1D1. Subsequent scans should consist of CT chest, abdomen and pelvis, and bone scan if bone metastases were present on the baseline studies. MRI is also an acceptable form of imaging. At the end of treatment, all scans (baseline and through disease progression) should be sent on a CD to the Vanderbilt Cancer Imaging Support Laboratory for central review and assessment. See Section 12.6 for details and address.

j Archived tumor biopsy specimen: Archived biopsy block (unstained slides) will be requested at the time of registration prior to initiation of treatment. See Section 11.1 for details. If archived tissue is not available, the VICC coordinating center should be contacted.

k After signing consent, a fresh tumor biopsy from a metastatic site amenable to a reasonably safe biopsy will be obtained prior to day 1 and upon progression of disease (see Section 11). The second biopsy will not be required for patients who are removed from study for reasons other than disease progression. A patient who undergoes a research biopsy procedure for the purpose of this protocol, in whom inadequate tissue is obtained, is not obligated to undergo a repeat biopsy.

l After cycle 6, at the discretion of the treating physician, carboplatin can be stopped and atezolizumab can be continued as a single agent.

m The dose of steroids before each carboplatin injection should be recorded. Prohibited medications (section 8) must be discontinued at least 7 days prior to initiation of study drug (C1D1).

n 1, 2, and 3 year follow-up can be done by phone or chart review to document survival status, and if applicate, the date of death and cause of death. Surveillance for Adverse Events of Special Interest (AESIs) (see section 13.5.12) should be included.

o Biopsy questionnaire should be filled out by the patient on C2D1. Form can be found in the packet of supplemental forms.

BRCA 1/2 testing and results are not required prior to enrollment. However, all patients in whom BRCA status is unknown must be referred for testing during the screening period. Testing must be sent for analysis prior to C3D1, although results need not be available. This is a standard procedure as per NCCN guidelines. The VICC coordinating center should be contacted if a patient's insurance will not pay for testing and an exception my be granted.

7.2 STUDY CALENDAR: ATEZOLIZUMAB (CROSSOVER)

Crossover therapy (atezolizumab) must begin no later than 21 days after the clinic visit at which progression is determined.

**Procedure**		**Cycle 1** [a] **(21-days)**	**Additional cycles**[a]	**End-of-treatment**[c]	**30 day follow-up**[b]	**1, 2, 3 year follow-up**[i]
		**Day 1**	**Day 1**			
Physical exam/signs and symptoms[c]		X	X	X		
Vital signs (including oxygen sat)		X	X	X		
ECOG Performance Status		X	X	X		
Blood collection	Clinical laboratory tests[d]	X	X	X		
	TSH, free T3, T4[e]	X		X (every 4th cycle)		
	Circulating tumor DNA	X	X (Cycle 2 only)	X		
	Mononuclear Cell Collection	X	X (Cycle 2 only)	X		
Tumor assessment CTs, bone scan[f]			X (every 3 cycles)			
Tumor biopsy (metastatic site) at crossover[g]		X				
Atezolizumab administration (IV)		X	X			
Concomitant medications[h]		X	X			
Adverse events		X	X		X	X[i]

a Evaluation assessments should be performed prior to administration of IMP unless otherwise indicated. Results should be reviewed by the Investigator prior to the administration of the next dose. All visits should occur within 3 days of scheduled visit unless otherwise noted. Delays up to 7 days will be considered by study chair. Crossover therapy (atezolizumab) must begin no later than 21 days after the clinic visit at which progression is determined. Exceptions must be discussed with the Protocol Chair.

b The 30-day follow-up assessments should be done 30 (±3 days) days after the last dose of study drugs. 30 day follow-up can be done by phone or by a clinic visit. Any ongoing toxicities should be documented. Any ongoing serious adverse events will be followed every 3 months until resolution. Any AEs or SAEs considered possibly or probably related to IMP that have not stabilized, returned to baseline, or are considered irreversible will continue to be monitored as per the protocol.

c The physical examination will include measurements of height and weight at screening. Weight will be measured on day 1 of each cycle and at the End-of-treatment visit. Physical examination will include examination of major body systems including neurologic, cardiac, respiratory, gastrointestinal, and skin. Clinically relevant signs and symptoms will be reported as AEs.

d Clinical laboratory tests will include hematology and serum chemistry panel including white blood cell count, differential, hemoglobin, platelet count, sodium, potassium, chloride, bicarbonate, BUN, creatinine, glucose, calcium, and magnesium, AST (SGOT), ALT (SGPT), and total serum bilirubin, albumin, and total protein. Additional tests will be performed when clinically appropriate. In case of Grade 3 or higher abnormal AST/ALT, additional testing will be done weekly until recovery to baseline value. Labs can be performed up to 3 days prior to each timepoint indicated in the study calendar.

e See section 9.1.4 for abnormal results

f Tumor assessment should be performed approximately every 3 cycles (up to -5 days) while on study treatment and at End-of-treatment visit if not obtained within the past 6 weeks. Scans should consist of CT scan of the chest, abdomen, and pelvis and a bone scan at baseline. Subsequent scans should consist of CT chest, abdomen and pelvis, and bone scan if bone metastases were present on the baseline studies. At the end of treatment, all scans (baseline and through disease progression) should be sent on a CD to the Vanderbilt Cancer Imaging Support Laboratory for central review and assessment. See Section 12 for details and address.

g Tumor biopsies from a metastatic site will be obtained from patients prior to day 1 (this will be the biopsy obtained at disease progression on the carboplatin only arm).

h The dose of steroids before each carboplatin injection should be recorded. See protocol for concomitant medications that are prohibited (section 8).

i 1, 2, and 3 year follow-up can be done by phone or chart review to document survival status, and if applicate, the date of death and cause of death. Surveillance for Adverse Events of Special Interest (AESIs) – see section 13.5.12 should be included.

8. TREATMENT PLAN

8.1 Overview

This is an open-label phase II multi-institution trial that evaluates the safety profile and anti-tumor activity of carboplatin +/- atezolizumab in patients with metastatic triple negative (ER negative, PR negative, HER2neu negative by IHC or FISH) breast cancer. Patients will be treated with carboplatin AUC 6 IV on day 1 and atezolizumab 1200 mg IV on day 1 of a 21 day cycle or carboplatin AUC 6 IV on day 1 of a 21 day cycle For patients on the combination arm, atezolizumab is to be administered prior to carboplatin. For patients on the combination arm, carboplatin can be stopped after cycle 6 and atezolizumab can be continued as a single-agent at the discretion of the treating physician.

8.2 Concomitant Treatment and Supportive Care Guidelines

8.2.1 Concomitant Therapy

Concomitant therapy includes any prescription medications or over-the-counter preparations used by a patient between the 7 days preceding Cycle 1, day 1 and the treatment discontinuation visit.

Neulasta (pegfilgrastim) may be given prophylactically per treating investigator discretion.

Patients who experience infusion-associated symptoms may be treated symptomatically with acetaminophen, ibuprofen, diphenhydramine, and/or cimetidine or another H2 receptor antagonist, as per standard practice. Serious infusion-associated events manifested by dyspnea, hypotension, wheezing, bronchospasm, tachycardia, reduced oxygen saturation, or respiratory distress should be managed with supportive therapies as clinically indicated (e.g., supplemental oxygen and β_2-adrenergic agonists).

Systemic corticosteroids and TNFα inhibitors may attenuate potential beneficial immunologic effects of treatment with atezolizumab but may be administered at the discretion of the treating physician. If feasible, alternatives to corticosteroids should be considered. The use of inhaled corticosteroids and mineralocorticoids (e.g., fludrocortisone) for patients with orthostatic hypotension or adrenocortical insufficiency is allowed. Megastrol administered as appetite stimulant is acceptable while the patient is enrolled in the study.

Patients who use prophylactic or therapeutic anticoagulation therapy (such as low-molecular-weight heparin or warfarin at a stable dose level), or other allowed maintenance therapy can continue their use. Males and females of reproductive potential should use highly effective means of contraception.

8.2.2 Excluded Therapy

Any concomitant therapy intended for the treatment of cancer, whether health authority-approved or experimental, is prohibited. This includes but is not limited to the following:

- Chemotherapy, hormonal therapy, immunotherapy, radiotherapy, investigational agents, or herbal therapy
  - After Cycle 1, certain forms of radiotherapy may be considered for pain palliation if patients are deriving benefit (e.g., treatment of known bony metastases); atezolizumab administration may be suspended during radiotherapy.

Patients must discontinue:

- Traditional herbal medicines because the ingredients of many herbal medicines are not fully studied and their use may result in unanticipated drug-drug interactions that may cause, or confound assessment of, toxicity
- The use of a RANKL inhibitor (denosumab); this agent could potentially alter the activity and the safety of atezolizumab. Bisphophonates may be used in lieu of RANKL inhibitors.

Patients are not allowed to receive immunostimulatory agents, including but not limited to IFN-α, IFN-γ, or IL-2, during the entire study. These agents, in combination with atezolizumab, could potentially increase the risk for autoimmune conditions.

Patients should also not be receiving immunosuppressive medications, including but not limited to cyclophosphamide, azathioprine, methotrexate, and thalidomide. These agents could potentially alter the activity and the safety of atezolizumab. Systemic corticosteroids and anti-TNFα agents may attenuate potential beneficial immunologic effects of treatment with atezolizumab but may be administered during the course of treatment at the discretion of the treating physician after discussion with the Protocol Chair. If feasible, alternatives to these agents should be considered.

In addition, all patients (including those who discontinue the study early) should not receive other immunostimulatory agents for 10 weeks after the last dose of atezolizumab.

8.3 Assessments during Treatment

All visits must occur within ± 3 days from the scheduled date, unless otherwise noted. Please see the Study Calendar at the beginning of this protocol for the schedule of treatment period assessments.

8.4 Cross-over post-progression

Patients who are randomized to carboplatin alone can cross over to receive atezolizumab upon disease progression based on clinical progression or radiographic progression. Patients who are randomized to carboplatin alone and who discontinue carboplatin for toxicity cannot cross over to receive single agent atezolizumab. There may be exceptions to this based on discussion with the Protocol Chair. However, patients on the carboplatin + atezolizumab arm can continue to receive single agent atezolizumab if they discontinue carboplatin due to toxicity. Patients who cross over to receive atezolizumab will follow the assessments and procedures as outlined in the study calendar. The duration of each cycle will continue to be 21 days and the cycle numbers will "reset" to 1 and will be followed by the “X” suffix (e.g.,

Cycles 1X, 2X, etc.). Crossover therapy (atezolizumab) must begin no later than 21 days after the clinic visit at which progression is determined.

Patients must have labs and procedures performed as outlined in the cross-over study calendar and must meet the values as described in the Inclusion Criteria, prior to receiving atezolizumab based on assessments and procedures performed within 21 days prior to Day 1 of Cycle 1X. Labs and procedures not included in the cross-over study calendar do not have to be repeated. Patients must also agree to a tumor biopsy, as outlined in the eligibility criteria, prior to initiating atezolizumab. Patients will receive crossover treatment until progression, intolerable toxicity, or elective withdrawal from the study. Patients who cross over will be evaluated with radiologic assessments every 3 cycles until disease progression.

8.5 Duration of Therapy

Duration of therapy will depend on individual response, evidence of disease progression and tolerance. In the absence of treatment delays due to adverse events, treatment may continue until one of criteria in Section 8.7 applies.

8.6 Duration of Follow-Up

Participants will be followed for 30 days for toxicity after the final dose of study drug. The 30 day follow-up can be done by phone or through a clinic visit. Patients who at the treatment completion visit have an ongoing serious adverse event or an adverse event leading to treatment discontinuation will be followed every 3 months until the event resolves, the investigator assesses the event as stable, or the patient is lost to follow-up. Participants will also be followed for disease progression by chart review yearly for 3 years from the date of last study drug administration. If the patient is no longer coming to the institution for care, then the patient may be called as needed for follow-up.

8.7 Criteria for Removal from Study

Patients will be removed from study when any of the criteria listed below apply. All patients who initiate protocol treatment will be included in the overall evaluation of response (intent-to-treat analysis). All reasons for discontinuation of therapy should be documented clearly in the medical record.

If a subject discontinues or withdraws from the study, every reasonable attempt will be made to get a tissue/tumor biopsy, along with blood, at the end of treatment, if the subject is able and willing.

Patients may be withdrawn from the study if they experience any of the following:

- Disease progression, per investigator assessment by RECIST (or irRECIST for atezolizumab arm)
- Intolerable toxicity to atezolizumab or carboplatin. Patients assigned to carboplatin + atezolizumab who experience toxicity to atezolizumab or carboplatin can continue on study with single agent carboplatin or atezolizumab, respectively, at the discretion of the treating physician. Patients will be followed for disease progression and should continue radiologic assessments every 3 cycles as per the study.

Other reasons for patient discontinuation may include, but are not limited to, the following:

- Non-compliance with the study protocol, including, but not limited to not attending the majority of scheduled visits. The Protocol Chair will determine when non-compliance

should lead to removal from study. Note: The patients will still be included in the overall evaluation of response (intent-to-treat analysis).
- Patient personal decision. The reason for discontinuation from the study must be documented. The patients will be included in the overall evaluation of response (intent-to-treat analysis) if any protocol therapy was administered prior to withdrawal.
- If the patient becomes pregnant
- Patient is lost to follow-up
- Study is terminated for any reason

The investigator has the right to discontinue a patient from the study for any medical condition that the investigator determines may jeopardize the patient's safety if he or she continues in the study; for reasons of noncompliance (e.g., missed doses, visits); or if the investigator determines it is in the best interest of the patient. The Protocol Chair should be notified in such cases.

9. EXPECTED TOXICITIES AND DOSING DELAYS/DOSE MODIFICATIONS

9.1 Anticipated Toxicities of atezolizumab (Atezolizumab) and Dose Modifications

Toxicities associated or possibly associated with atezolizumab treatment should be managed according to standard medical practice. Additional tests, such as autoimmune serology or biopsies, should be used to evaluate for a possible immunogenic etiology, when clinically indicated. Although most immune-mediated adverse events observed with atezolizumab have been mild and self-limiting, such events should be recognized early and treated promptly to avoid potential major complications. Discontinuation of atezolizumab may not have an immediate therapeutic effect, and in severe cases, immune-mediated toxicities may require acute management with topical corticosteroids, systemic corticosteroids, or other immunosuppressive agents.

The investigator should consider the benefit□risk balance for a given patient prior to further administration of atezolizumab. In patients who have met the criteria for permanent iscontinuation, resumption of atezolizumab may be considered if the patient is deriving benefit and has fully recovered from the immune-mediated event. Patients can be re-challenged with atezolizumab only after approval has been documented by both the investigator (or an appropriate delegate) and the Medical Monitor. Guidelines for managing patients who experience selected adverse events are provided in the following sections. Management guidelines are presented by adverse event severity based on the National Cancer Institute (NCI) Common Terminology Criteria for Adverse Events (CTCAE) and in some instances may vary by CTCAE version (4.0 vs.
5.0).

Management Guidelines for Renal Events

Event	Management
Renal event, Grade 1	• Continue atezolizumab.<br>• Monitor kidney function, including creatinine, closely until values resolve to within normal limits or to baseline values.
Renal event, Grade 2	• Withhold atezolizumab for up to 12 weeks after event onset. [a]<br>• Refer patient to renal specialist.<br>• Initiate treatment with corticosteroids equivalent to 1-2 mg/kg/day oral prednisone.<br>• If event resolves to Grade 1 or better, resume atezolizumab. [b]<br>• If event does not resolve to Grade 1 or better while withholding atezolizumab, permanently discontinue atezolizumab and contact Study Chair. [c]

Renal event, Grade 3 or 4	• Permanently discontinue atezolizumab and contact Study Chair.<br>• Refer patient to renal specialist and consider renal biopsy.<br>• Initiate treatment with corticosteroids equivalent to 1-2 mg/kg/day oral prednisone.<br>• If event does not improve within 48 hours after initiating corticosteroids, consider adding an immunosuppressive agent.<br>• If event resolves to Grade 1 or better, taper corticosteroids over ≥ 1 month.

[a] Atezolizumab may be withheld for a longer period of time (i.e., > 12 weeks after event onset) to allow for corticosteroids (if initiated) to be reduced to the equivalent of ≤10 mg/day oral prednisone. The acceptable length of the extended period of time must be agreed upon by the investigator and the Study Chair.

[b] If corticosteroids have been initiated, they must be tapered over ≥ 1 month to the equivalent of ≤10 mg/day oral prednisone before atezolizumab can be resumed.

[c] Resumption of atezolizumab may be considered in patients who are deriving benefit and have fully recovered from the immune-mediated event. Patients can be re-challenged with atezolizumab only after approval has been documented by both the investigator (or an appropriate delegate) and the Study Chair.

9.2 General Guidelines for Dosage Modification and Treatment Interruption or Discontinuation

Atezolizumab treatment will be given as long as the patient continues to experience clinical benefit in the opinion of the investigator until the earlier of unacceptable toxicity, symptomatic deterioration attributed to disease progression, or any of the other reasons for treatment discontinuation listed above.

There will be no dose reduction for atezolizumab in this study. **Patients may temporarily suspend study treatment for up to 84 days beyond the scheduled date of delayed infusion if study drug-related toxicity requiring dose suspension is experienced.** If atezolizumab is held because of AEs for > 84 days beyond the scheduled date of infusion, the patient will be discontinued from atezolizumab and will be followed for safety and efficacy as specified in the protocol.

If a patient must be tapered off steroids used to treat AEs, atezolizumab may be held for additional time beyond 84 days from the scheduled dose until steroids are discontinued or reduced to a prednisone dose (or dose equivalent) of ≤ 10 mg/day. The acceptable length of interruption will be at the discretion of the investigator.

Dose interruptions for reasons other than toxicity, such as surgical procedures, may be allowed. The acceptable length of interruption will be at the discretion of the Protocol Chair.

Patients should be assessed clinically (including review of laboratory values) for toxicity prior to, during, and after each infusion. If unmanageable toxicity due to atezolizumab occurs at any time during the study, treatment with atezolizumab should be discontinued.

Management of hepatitis/transaminitis, colitis, rash, and hypothyroidism are presented in this section as they have been observed in this study and are potentially immune related.

9.2.1 Gastrointestinal Toxicity

Immune-mediated colitis has been associated with the administration of atezolizumab.

Patients should be advised to inform the investigator if any diarrhea occurs, even if it is mild.

If the event is of significant duration or magnitude, or is associated with signs of systemic inflammation or acute phase reactants (e.g., increased CRP or platelet count or bandemia), it is recommended that sigmoidoscopy (or colonoscopy, if appropriate) with colonic biopsy with three to five specimens for standard paraffin block be performed. If possible, one or two biopsy specimens should be snap frozen and stored.

Treatment may be restarted following the resolution of colitis. In addition, if the patient is being managed with corticosteroids, treatment should not be restarted until the steroids have been tapered down to a prednisone dose ≤ 10 mg/day. Patients who resume treatment should be monitored closely for signs of renewed diarrhea.

Management Guidelines for Gastrointestinal Events (Diarrhea or Colitis)

Event	Management
Diarrhea or colitis, Grade 1	• Continue atezolizumab.<br>• Initiate symptomatic treatment.<br>• Endoscopy is recommended if symptoms persist for > 7 days.<br>• Monitor closely.
Diarrhea or colitis, Grade 2	• Withhold atezolizumab for up to 12 weeks after event onset. [a]<br>• Initiate symptomatic treatment.<br>• Patient referral to GI specialist is recommended.<br>• For recurrent events or events that persist >5 days, initiate treatment with corticosteroids equivalent to 1-2 mg/kg/day oral prednisone.<br>• If event resolves to Grade 1 or better, resume atezolizumab. [b]<br>• If event does not resolve to Grade 1 or better while withholding atezolizumab, permanently discontinue atezolizumab and contact Study Chair. [c]
Diarrhea or colitis, Grade 3	• Withhold atezolizumab for up to 12 weeks after event onset. [a]<br>• Refer patient to GI specialist for evaluation and confirmatory biopsy.<br>• Initiate treatment with corticosteroids equivalent to 1-2 mg/kg/day IV methylprednisolone and convert to 1-2 mg/kg/day oral prednisone or equivalent upon improvement.<br>• If event resolves to Grade 1 or better, resume atezolizumab. [b]<br>• If event does not resolve to Grade 1 or better while

	withholding atezolizumab, permanently discontinue atezolizumab and contact Study Chair. [c]

GI = gastrointestinal.

[a] Atezolizumab may be withheld for a longer period of time (i.e., >12 weeks after event onset) to allow for corticosteroids (if initiated) to be reduced to the equivalent of ≤ 10 mg/day oral prednisone. The acceptable length of the extended period of time must be agreed upon by the investigator and the Study Chair.

[b] If corticosteroids have been initiated, they must be tapered over ≥ 1 month to the equivalent of ≤ 10 mg/day oral prednisone before atezolizumab can be resumed.

[c] Resumption of atezolizumab may be considered in patients who are deriving benefit and have fully recovered from the immune-related event. Patients can be re-challenged with atezolizumab only after approval has been documented by both the investigator (or an appropriate delegate) and the Study Chair.

9.2.2 Hepatotoxicity

Immune-mediated hepatitis has been associated with the administration of atezolizumab.

While in this study, patients presenting with right upper-quadrant abdominal pain felt by the investigator to be possibly related to study treatment and/or unexplained nausea or vomiting should have LFTs performed immediately, and LFTs (AST/ALT) should be reviewed before administration of the next dose of study drug.

In the presence of LFT abnormalities, neoplastic, concurrent medications, viral hepatitis, and toxic etiologies should be considered and addressed, as appropriate. Imaging of the liver, gall bladder, and biliary tree should be performed to rule out neoplastic or other causes of increased LFTs. Anti-nuclear antibody, perinuclear anti-neutrophil cytoplasmic antibody, anti-liver kidney microsomal, and anti-smooth muscle antibody tests should be performed if an autoimmune etiology is suspected.

Patients with LFT abnormalities should be managed according to the guidelines in table below.

Dose Modification Guidelines for Hepatic Events

Event	Management
Hepatic event, Grade 1	**CTCAE v4.0 guidelines**<br>• Continue atezolizumab.<br>• Monitor LFTs until values resolve to within normal limits.<br><br>**CTCAE v5.0 guidelines**<br>• Continue atezolizumab.

	• Monitor LFTs until values resolve to within normal limits or to baseline values.
Hepatic event, Grade 2	**All events:**<br>• Monitor LFTs more frequently until return to baseline values.<br>**Events of > 5 days' duration:**<br>• Withhold atezolizumab for up to 12 weeks after event onset. aa<br>• Initiate treatment with corticosteroids equivalent to 1-2 mg/kg/day oral prednisone.<br>• If event resolves to Grade 1 or better, resume atezolizumab. [b]<br>• If event does not resolve to Grade 1 or better while withholding atezolizumab, permanently discontinue atezolizumab and contact Study Chair. [c]
Event	Management
Hepatic event, Grade 3 or 4	• Permanently discontinue atezolizumab and contact Study Chair. [c]<br>• Consider patient referral to gastrointestinal specialist for evaluation and liver biopsy to establish etiology of hepatic injury.<br>• Initiate treatment with corticosteroids equivalent to 1-2 mg/kg/day oral prednisone.<br>• If event does not improve within 48 hours after initiating corticosteroids, consider adding an immunosuppressive agent.<br>• If event resolves to Grade 1 or better, taper corticosteroids over ≥ 1 month.

LFT = liver function tests. CTCAE = Common Terminology Criteria for Adverse Events

[a] Atezolizumab may be withheld for a longer period of time (i.e., > 12 weeks after event onset) to allow for corticosteroids (if initiated) to be reduced to the equivalent of ≤ 10 mg/day oral prednisone. The acceptable length of the extended period of time must be agreed upon by the investigator and the Study Chair.

[b] If corticosteroids have been initiated, they must be tapered over ≥ 1 month to the equivalent of ≤ 10 mg/day oral prednisone before atezolizumab can be resumed.

[c] Resumption of atezolizumab may be considered in patients who are deriving benefit and have fully recovered from the immune-related event. Patients can be re-challenged with atezolizumab only after approval has been documented by both the investigator (or an appropriate delegate) and the Study Chair.

9.2.3 Dermatologic Toxicity

Treatment-emergent rash has been associated with atezolizumab. The majority of cases of rash were mild in severity and self-limited, with or without pruritus.

A dermatologist should evaluate persistent and/or severe rash or pruritus. A biopsy should be performed unless contraindicated. Low-grade rash and pruritus irAEs have been treated with symptomatic therapy (e.g., antihistamines). Topical or parenteral corticosteroids may be required for more severe symptoms.

Dermatologic toxicity and rash should be managed according to the guidelines in table below.

Dose Modification Guidelines for Dermatologic Events

Event	Management
Dermatologic event, Grade 1	• Continue atezolizumab.<br>• Consider treatment with topical corticosteroids and/or other symptomatic therapy (e.g. antihistamines).
Dermatologic event, Grade 2	• Continue atezolizumab.<br>• Consider patient referral to dermatologist.<br>• Initiate treatment with topical corticosteroids.<br>• Consider treatment with higher-potency topical corticosteroids if event does not improve.<br>• Initiate treatment with higher-potency topical corticosteroids if event does not improve.
Dermatologic event, Grade 3	• Withhold atezolizumab for up to 12 weeks after event onset. [a]<br>• Refer patient to dermatologist.<br>• Initiate treatment with corticosteroids equivalent to 10mg/day oral prednisone, increasing dose to 1-2 mg/kg/day if event does not improve within 48-72 hours.<br>• If event resolves to Grade 1 or better, resume atezolizumab.[b]<br>• If event does not resolved to Grade 1 or better while withholding atezolizumab, permanently discontinue ateazolizumab and contact Study Chair.[c]
Dermatologic event, Grade 4	• Permanently discontinue atezolizumab and contact Study Chair.[c]

[a] Atezolizumab may be withheld for a longer period of time (i.e., > 12 weeks after event onset) to allow for corticosteroids (if initiated) to be reduced to the equivalent of ≤ 10 mg/day oral prednisone. The acceptable length of the extended period of time must be agreed upon by the investigator and the Stud Chair.

[b] If corticosteroids have been initiated, they must be tapered over ≥ 1 month to the equivalent of ≤ 10 mg/day oral prednisone before atezolizumab can be resumed.

[c] Resumption of atezolizumab may be considered in patients who are deriving benefit and have fully recovered from the immune-mediated event. Patients can be re- challenged with atezolizumab only after approval has been documented by both the investigator (or an appropriate delegate) and the Study Chair.

9.2.4 Endocrine Toxicity

Hypothyroidism has been associated with the administration of atezolizumab.

Patients with unexplained symptoms such as fatigue, myalgias, impotence, mental status changes, or constipation should be investigated for the presence of thyroid, pituitary, or adrenal endocrinopathies, as well as for hyponatremia or hyperkalemia. An endocrinologist should be consulted if an endocrinopathy is suspected. Thyroid-stimulating hormone (TSH) and free T4 levels should be obtained to determine whether thyroid abnormalities are present. TSH, prolactin, and a morning cortisol level will help to differentiate primary adrenal insufficiency from primary pituitary insufficiency.

Hypothyroidism should be managed according to the guidelines in the table below.

Management Guidelines for Endocrine Events

Event	Management
Asymptomatic hypothyroidism	• Continue atezolizumab.<br>• Initiate treatment with thyroid replacement hormone.<br>• Monitor TSH weekly.
Symptomatic hypothyroidism	• Withhold atezolizumab.<br>• Initiate treatment with thyroid replacement hormone.<br>• Monitor TSH weekly.<br>• Consider patient referral to endocrinologist.<br>• Resume atezolizumab when symptoms are controlled and thyroid function is improving.
Asymptomatic hyperthyroidism	**TSH ≥ 0.1 mU/L and < 0.5 mU/L:**<br>• Continue atezolizumab.<br>• Monitor TSH every 4 weeks.<br>**TSH < 0.1 mU/L:**<br>• Follow guidelines for symptomatic hyperthyroidism.
Symptomatic hyperthyroidism	• Withhold atezolizumab.<br>• Initiate treatment with anti-thyroid drug such as methimazole or carbimazole as needed.<br>• Consider patient referral to endocrinologist.<br>• Resume atezolizumab when symptoms are controlled and thyroid function is improving.<br>• Permanently discontinue atezolizumab and contact Study Chair for life-threatening immune-related hyperthyroidism.[c]

MRI=magnetic resonance imaging; TSH = thyroid-stimulating hormone.
Note: Management guidelines are presented by adverse event severity based on NCI CTCAE and are applicable to both CTCAE Version 4.0 and CTCAE Version 5.0

[a] Atezolizumab may be withheld for a longer period of time (i.e., >12 weeks after event onset) to allow for corticosteroids (if initiated) to be reduced to the equivalent of ≤ 10 mg/day oral prednisone. The acceptable length of the extended period of time must be agreed upon by the investigator and the Study Chair.

[b] If corticosteroids have been initiated, they must be tapered over ≥1 month to the equivalent of ≤10 mg/day oral prednisone before atezolizumab can be resumed.

[c] Resumption of atezolizumab may be considered in patients who are deriving benefit and have fully recovered from the immune-related event. Patients can be re-challenged with atezolizumab only after approval has been documented by both the investigator (or an appropriate delegate) and the Study Chair.

Management Guidelines for Endocrine Events (cont.)

Event	Management
Symptomatic adrenal insufficiency, Grade 2-4	• Withhold atezolizumab for up to 12 weeks after event onset. a<br>• Refer patient to endocrinologist.<br>• Perform appropriate imaging.<br>• Initiate treatment with corticosteroids equivalent to 1-2 mg/kg/day IV methylprednisolone and convert to 1-2 mg/kg/day oral prednisone or equivalent upon improvement.<br>• If event resolves to Grade 1 or better and patient is stable on replacement therapy, resume atezolizumab. [b]<br>• If event does not resolve to Grade 1 or better or patient is not stable on replacement therapy while withholding atezolizumab, permanently discontinue atezolizumab and contact Study Chair. [c]
Hyperglycemia, Grade 1 or 2	• Continue atezolizumab.<br>• Investigate for diabetes. If patient has Type 1 diabetes, treat as a Grade 3 event. If patient does not have Type 1 diabetes, treat as per institutional guidelines.<br>• Monitor for glucose control.
Hyperglycemia, Grade 3 or 4	• Withhold atezolizumab.<br>• Initiate treatment with insulin.<br>• Monitor for glucose control.<br>• Resume atezolizumab when symptoms resolve and glucose levels are stable.
Hypophysitis (pan-hypopituitarism), Grade 2 or 3	• Withhold atezolizumab for up to 12 weeks after event onset. a<br>• Refer patient to endocrinologist.<br>• Perform brain MRI (pituitary protocol).<br>• Initiate treatment with corticosteroids equivalent to 1-2 mg/kg/day IV methylprednisolone and convert to 1-2 mg/kg/day oral prednisone or equivalent upon improvement.<br>• Initiate hormone replacement if clinically indicated.<br>• If event resolves to Grade 1 or better, resume atezolizumab. [b]<br>• If event does not resolve to Grade 1 or better while withholding atezolizumab, permanently discontinue atezolizumab and contact Study Chair. [c]

	• For recurrent hypophysitis, treat as a Grade 4 event.

MRI=magnetic resonance imaging; TSH = thyroid-stimulating hormone.

[a] Atezolizumab may be withheld for a longer period of time (i.e., >12 weeks after event onset) to allow for corticosteroids (if initiated) to be reduced to the equivalent of ≤ 10 mg/day oral prednisone. The acceptable length of the extended period of time must be agreed upon by the investigator and the Study Chair.

[b] If corticosteroids have been initiated, they must be tapered over ≥1 month to the equivalent of ≤ 10 mg/day oral prednisone before atezolizumab can be resumed.

[c] Resumption of atezolizumab may be considered in patients who are deriving benefit and have fully recovered from the immune-related event. Patients can be re-challenged with atezolizumab only after approval has been documented by both the investigator (or an appropriate delegate) and the Study Chair.

Management Guidelines for Endocrine Events (cont.)

Event	Management
Hypophysitis (pan-hypopituitarism), Grade 4	• Permanently discontinue atezolizumab and contact Study Chair. [c]<br>• Refer patient to endocrinologist.<br>• Perform brain MRI (pituitary protocol).<br>• Initiate treatment with corticosteroids equivalent to 1-2 mg/kg/day IV methylprednisolone and convert to 1-2 mg/kg/day oral prednisone or equivalent upon improvement.<br>• Initiate hormone replacement if clinically indicated.

MRI = magnetic resonance imaging; TSH = thyroid-stimulating hormone.

[a] Atezolizumab may be withheld for a longer period of time (i.e., >12 weeks after event onset) to allow for corticosteroids (if initiated) to be reduced to the equivalent of ≤10 mg/day oral prednisone. The acceptable length of the extended period of time must be agreed upon by the investigator and the Study Chair.

[b] If corticosteroids have been initiated, they must be tapered over ≥ 1 month to the equivalent of ≤ 10 mg/day oral prednisone before atezolizumab can be resumed.

[c] Resumption of atezolizumab may be considered in patients who are deriving benefit and have fully recovered from the immune-related event. Patients can be re-challenged with atezolizumab only after approval has been documented by both the investigator (or an appropriate delegate) and the Study Chair.

9.2.5 Pulmonary Toxicity

Dyspnea, cough, fatigue, hypoxia, pneumonitis, and pulmonary infiltrates have been associated with the administration of atezolizumab and have primarily been observed in patients with underlying NSCLC.

Mild-to-moderate events of pneumonitis have been reported with atezolizumab. All pulmonary events should be thoroughly evaluated for other commonly reported etiologies such as pneumonia/infection, lymphangitic carcinomatosis, pulmonary embolism, heart failure, chronic obstructive pulmonary disease (COPD), or pulmonary hypertension and the following should be performed:

- Measurement of oxygen saturation (i.e., arterial blood gas)
- High-resolution CT scan of the chest
- Bronchoscopy with bronchoalveolar lavage and biopsy
- Pulmonary function tests (with diffusion capacity of the lung for carbon monoxide [DL_{CO}])

Patients will be assessed for pulmonary signs and symptoms throughout the study. Patients will also have CT scans of the chest at every tumor assessment.

Pulmonary toxicity should be managed according to the guidelines in the table below.

Management Guidelines for Pulmonary Events, Including Pneumonitis

Event	Management
Pulmonary event, Grade 1	• Continue atezolizumab and monitor closely.<br>• Re-evaluate on serial imaging.<br>• Consider patient referral to pulmonary specialist.
Pulmonary event, Grade 2	• Withhold atezolizumab for up to 12 weeks after event onset. [a]<br>• Refer patient to pulmonary and infectious disease specialists and consider bronchoscopy or BAL.<br>• Initiate treatment with corticosteroids equivalent to 1-2 mg/kg/day oral prednisone.<br>• If event resolves to Grade 1 or better, resume atezolizumab. [b]<br>• If event does not resolve to Grade 1 or better while withholding atezolizumab, permanently discontinue atezolizumab and contact Study Chair. [c]<br>• For recurrent events, treat as a Grade 3 or 4 event.
Pulmonary event, Grade 3 or 4	• Permanently discontinue atezolizumab and contact Study Chair. [c]<br>• Bronchoscopy or BAL is recommended.<br>• Initiate treatment with corticosteroids equivalent to 1-2 mg/kg/day oral prednisone.<br>• If event does not improve within 48 hours after initiating corticosteroids, consider adding an immunosuppressive agent.<br>• If event resolves to Grade 1 or better, taper corticosteroids over ≥1 month.

BAL = bronchoscopic alveolar lavage.

[a] Atezolizumab may be withheld for a longer period of time (i.e., >12 weeks after event onset) to allow for corticosteroids (if initiated) to be reduced to the equivalent of ≤10 mg/day oral

prednisone. The acceptable length of the extended period of time must be agreed upon by the investigator and the Study Chair.

[b] If corticosteroids have been initiated, they must be tapered over ≥1 month to the equivalent of ≤ 10 mg/day oral prednisone before atezolizumab can be resumed.

[c] Resumption of atezolizumab may be considered in patients who are deriving benefit and have fully recovered from the immune-related event. Patients can be re-challenged with atezolizumab only after approval has been documented by both the investigator (or an appropriate delegate) and the Study Chair

9.2.6 Potential Pancreatic Toxicity

Management Guidelines for Pancreatic Events, Including Pancreatitis

Event	Management
Amylase and/or lipase elevation, Grade 2	**Amylase and/or lipase > 1.5-2.0 x ULN:**<br>• Continue atezolizumab.<br>• Monitor amylase and lipase weekly.<br>• For prolonged elevation (e.g., >3 weeks), consider treatment with corticosteroids equivalent to 10 mg/day oral prednisone.<br>**Asymptomatic with amylase and/or lipase > 2.0-5.0 xULN:**<br>• Treat as a Grade 3 event.
Amylase and/or lipase elevation, Grade 3 or 4	• Withhold atezolizumab for up to 12 weeks after event onset. [a]<br>• Refer patient to GI specialist.<br>• Monitor amylase and lipase every other day.<br>• If no improvement, consider treatment with corticosteroids equivalent to 1-2 mg/kg/day oral prednisone.<br>• If event resolves to Grade 1 or better, resume atezolizumab. [b]<br>• If event does not resolve to Grade 1 or better while withholding atezolizumab, permanently discontinue atezolizumab and contact Study Chair. [c]<br>• For recurrent events, permanently discontinue atezolizumab and contact Study Chair. [c]

GI = gastrointestinal.

[a] Atezolizumab may be withheld for a longer period of time (i.e., > 12 weeks after event onset) to allow for corticosteroids (if initiated) to be reduced to the equivalent of ≤ 10 mg/day oral prednisone. The acceptable length of the extended period of time must be agreed upon by the investigator and the Study Chair.

[b] If corticosteroids have been initiated, they must be tapered over ≥ 1 month to the equivalent of ≤ 10 mg/day oral prednisone before atezolizumab can be resumed.

[c] Resumption of atezolizumab may be considered in patients who are deriving benefit and have

fully recovered from the immune-related event. Patients can be re-challenged with atezolizumab only after approval has been documented by both the investigator (or an appropriate delegate) and the Study Chair.

Management Guidelines for Pancreatic Events, Including Pancreatitis (cont.)

Event	Management
Immune-related pancreatitis, Grade 2 or 3	• Withhold atezolizumab for up to 12 weeks after event onset. [a]<br>• Refer patient to GI specialist.<br>• Initiate treatment with corticosteroids equivalent to 1-2 mg/kg/day IV methylprednisolone and convert to 1-2 mg/kg/day oral prednisone or equivalent upon improvement.<br>• If event resolves to Grade 1 or better, resume atezolizumab. [b]<br>• If event does not resolve to Grade 1 or better while withholding atezolizumab, permanently discontinue atezolizumab and contact Study Chair. [c]<br>• For recurrent events, permanently discontinue atezolizumab and contact Study Chair. [c]
Immune-related pancreatitis, Grade 4	• Permanently discontinue atezolizumab and contact Study Chair. [c]<br>• Refer patient to GI specialist.<br>• Initiate treatment with corticosteroids equivalent to 1-2 mg/kg/day IV methylprednisolone and convert to 1-2 mg/kg/day oral prednisone or equivalent upon improvement.<br>• If event does not improve within 48 hours after initiating corticosteroids, consider adding an immunosuppressive agent.<br>• If event resolves to Grade 1 or better, taper corticosteroids over ≥ 1 month.

GI =gastrointestinal.

[a] Atezolizumab may be withheld for a longer period of time (i.e., > 12 weeks after event onset) to allow for corticosteroids (if initiated) to be reduced to the equivalent of ≤ 10 mg/day oral prednisone. The acceptable length of the extended period of time must be agreed upon by the investigator and the Study Chair.

[b] If corticosteroids have been initiated, they must be tapered over ≥ 1 month to the equivalent of ≤ 10 mg/day oral prednisone before atezolizumab can be resumed.

[c] Resumption of atezolizumab may be considered in patients who are deriving benefit and have fully recovered from the immune-related event. Patients can be re-challenged with atezolizumab only after approval has been documented by both the investigator (or an appropriate delegate) and the Study Chair.

9.2.7 Potential Eye Toxicity

An ophthalmologist should evaluate visual complaints. Uveitis or episcleritis may be treated with topical corticosteroid eye drops. Atezolizumab should be permanently discontinued for immune-mediated ocular disease that is unresponsive to local immunosuppressive therapy.

Potential eye toxicity should be managed according to the guidelines in the table below.

Management Guidelines for Ocular Events

Event	Management
Ocular event, Grade 1	• Continue atezolizumab.<br>• Patient referral to ophthalmologist is strongly recommended.<br>• Initiate treatment with topical corticosteroid eye drops and topical immunosuppressive therapy.<br>• If symptoms persist, treat as a Grade 2 event.
Ocular event, Grade 2	• Withhold atezolizumab for up to 12 weeks after event onset. [a]<br>• Patient referral to ophthalmologist is strongly recommended.<br>• Initiate treatment with topical corticosteroid eye drops and topical immunosuppressive therapy.<br>• If event resolves to Grade 1 or better, resume atezolizumab. [b]<br>• If event does not resolve to Grade 1 or better while withholding atezolizumab, permanently discontinue atezolizumab and contact Study Chair. [c]
Ocular event, Grade 3 or 4	• Permanently discontinue atezolizumab and contact Study Chair. [c]<br>• Refer patient to ophthalmologist.<br>• Initiate treatment with corticosteroids equivalent to 1-2 mg/kg/day oral prednisone.<br>• If event resolves to Grade 1 or better, taper corticosteroids over ≥ 1 month.

[a] Atezolizumab may be withheld for a longer period of time (i.e., > 12 weeks after event onset) to allow for corticosteroids (if initiated) to be reduced to the equivalent of ≤ 10 mg/day oral prednisone. The acceptable length of the extended period of time must be agreed upon by the investigator and the Study Chair.

[b] If corticosteroids have been initiated, they must be tapered over ≥ 1 month to the equivalent of ≤ 10 mg/day oral prednisone before atezolizumab can be resumed.

[c] Resumption of atezolizumab may be considered in patients who are deriving benefit and have fully recovered from the immune-related event. Patients can be re-challenged with atezolizumab only after approval has been documented by both the investigator (or an appropriate delegate) and the Study Chair.

9.2.8 Immune-mediated Myocarditis

Immune-mediated myocarditis has been associated with the administration of atezolizumab. Immune-mediated myocarditis should be suspected in any patient presenting with signs or symptoms suggestive of myocarditis, including, but not limited to, laboratory (e.g., BNP [B-Natriuretic Peptide]) or cardiac imaging abnormalities, dyspnea, chest pain, palpitations, fatigue, decreased exercise tolerance, or syncope.

Immune-mediated myocarditis needs to be distinguished from myocarditis resulting from infection (commonly viral, e.g., in a patient who reports a recent history of gastrointestinal illness), ischemic events, underlying arrhythmias, exacerbation of preexisting cardiac conditions, or progression of malignancy.

All patients with possible myocarditis should be urgently evaluated by performing cardiac enzyme assessment, an ECG, a chest X-ray, an echocardiogram, and a cardiac MRI as appropriate per institutional guidelines. A cardiologist should be consulted. An endomyocardial biopsy may be considered to enable a definitive diagnosis and appropriate treatment, if clinically indicated.

Patients with signs and symptoms of myocarditis, in the absence of an identified alternate etiology, should be treated according to the guidelines provided below.

Management Guidelines for Immune-Mediated Myocarditis

Event	Management
Immune-mediated myocarditis, Grade 1 **(CTCAE v4.0 guidelines only)**	**CTCAE v4.0 guidelines**<br>• Refer patient to cardiologist.<br>• Initiate treatment as per institutional guidelines.
Immune-mediated myocarditis, Grade 2	• Withhold atezolizumab for up to 12 weeks after event onset[a] and contact Study Chair.<br>• Refer patient to cardiologist.<br>• Initiate treatment as per institutional guidelines and consider antiarrhythmic drugs, temporary pacemaker, ECMO, or VAD as appropriate.<br>• Consider treatment with corticosteroids equivalent to 1-2 mg/kg/day IV methylprednisolone and convert to 1-2 mg/kg/day oral prednisone or equivalent upon improvement.<br>• If event resolves to Grade 1 or better, resume atezolizumab.[b]<br>• If event does not resolve to Grade 1 or better while withholding atezolizumab, permanently discontinue atezolizumab and contact Study Chair.[c]

Event	Management
Immune-mediated myocarditis, Grade 1 **(CTCAE v4.0 guidelines only)**	**CTCAE v4.0 guidelines**<br>• Refer patient to cardiologist.<br>• Initiate treatment as per institutional guidelines.
Immune-mediated myocarditis, Grade 3-4	• Permanently discontinue atezolizumab and contact Study Chair.[c]<br>• Refer patient to cardiologist.<br>• Initiate treatment as per institutional guidelines and consider antiarrhythmic drugs, temporary pacemaker, ECMO, or VAD as appropriate.<br>• Initiate treatment with corticosteroids equivalent to 1-2 mg/kg/day IV methylprednisolone and convert to 1-2 mg/kg/day oral prednisone or equivalent upon improvement.<br>• If event does not improve within 48 hours after initiating corticosteroids, consider adding an immunosuppressive agent.<br>• If event resolves to Grade 1 or better, taper corticosteroids over ≥ 1 month.

ECMO = extracorporeal membrane oxygenation; VAD = ventricular assist device.

[a] Atezolizumab may be withheld for longer period of time (i.e., >12 weeks after event onset) to allow for corticosteroids (if initiated) to be reduced to the equivalent of ≤ 10 mg/day oral prednisone. The acceptable length of the extended period of time must be agreed upon by the investigator and the Study Chair.
[b] If corticosteroids have been initiated, they must be tapered over ≥ 1 month to the equivalent of ≤ 10 mg/day oral prednisone before ateolizumab can be resumed.
[c] Resumption of atezolizumab may be considered in patients who are deriving benefit and have fully recovered from the immune-mediated event. Patients can be re-challenged with atezolizumab only after approval has been documented by both the investigator (or an appropriate delegate) and the Study Chair.

9.2.9 Infusion-related reactions and Cytokine-Release Syndrome

No premedication is indicated for the administration of Cycle 1 of atezolizumab. However, patients who experience an infusion-related reaction (IRR) or cytokine-release syndrome (CRS) with atezolizumab may receive premedication with antihistamines, antipyretics, and/or analgesics (e.g., acetaminophen) for subsequent infusions. Metamizole (dipyrone) is prohibited in treating atezolizumab-associated IRRs because of its potential for causing agranulocytosis.

IRRs are known to occur with the administration of monoclonal antibodies and have been reported with atezolizumab. These reactions, which are thought to be due to release of cytokines and/or other

chemical mediators, occur within 24 hours of atezolizumab administration and are generally mild to moderate in severity.

CRS is defined as a supraphysiologic response following administration of any immune therapy that results in activation or engagement of endogenous or infused T cells and/or other immune effector cells. Symptoms can be progressive, always include fever at the onset, and may include hypotension, capillary leak (hypoxia), and end-organ dysfunction (Lee et al. 2019). CRS has been well documented with chimeric antigen receptor T-cell therapies and bispecific T-cell engager antibody therapies but has also been reported with immunotherapies that target PD-1 or PD-L1 (Rotz et al. 2017; Adashek and Feldman 2019), including atezolizumab.

There may be significant overlap in signs and symptoms of IRRs and CRS, and in recognition of the challenges in clinically distinguishing between the two, consolidated guidelines for medical management of IRRs and CRS are provided in the table below.

Management Guidelines for Infusion-Related Reactions and Cytokine-Release Syndrome

Event	Management
Grade 1 **a**<br>Fever **b** with or without constitutional symptoms	• Immediately interrupt infusion.<br>• Upon symptom resolution, wait for 30 minutes and then restart infusion at half the rate being given at the time of event onset.<br>• If the infusion is tolerated at the reduced rate for 30 minutes, the infusion rate may be increased to the original rate.<br>• If symptoms recur, discontinue infusion of this dose. Administer symptomatic treatment, **c** including maintenance of IV fluids for hydration.<br>• In case of rapid decline or prolonged CRS (> 2 days) or in patients with significant symptoms and/or comorbidities, onsider managing as per Grade 2.<br>• For subsequent infusions, consider administration of oral premedication with antihistamines, anti-pyretics, and/or analgesics, and monitor closely for IRRs and/or CRS.
Grade 2 **a**<br>Fever **b** with hypotension not requiring vasopressors<br>**and/or**<br>Hypoxia requiring low-flow oxygen d by nasal cannula or blow-by	• Immediately interrupt infusion.<br>• Upon symptom resolution, wait for 30 minutes and then restart infusion at half the rate being given at the time of event onset.<br>• If symptoms recur, discontinue infusion of this dose.<br>• Administer symptomatic treatment. **c**<br>• For hypotension, administer IV fluid bolus as needed.<br>• Monitor cardiopulmonary and other organ function closely (in the ICU, if appropriate). Administer IV fluids as clinically indicated, and manage constitutional symptoms and organ toxicities as per institutional practice.

	• Rule out other inflammatory conditions that can mimic CRS<br>• (e.g., sepsis). If no improvement within 24 hours, initiate workup and assess for signs and symptoms of HLH or MAS.<br>• Consider IV corticosteroids (e.g., methylprednisolone 2 mg/kg/day or dexamethasone 10 mg every 6 hours).<br>• Consider anti-cytokine therapy. **e**<br>• Consider hospitalization until complete resolution of symptoms. If no improvement within 24 hours, manage as per Grade 3, that is, hospitalize patient (monitoring in the ICU is recommended), permanently discontinue atezolizumab, and contact Study Chair.<br>• If symptoms resolve to Grade 1 or better for 3 consecutive days, the next dose of atezolizumab may be administered. For subsequent infusions, consider administration of oral premedication with antihistamines, anti-pyretics, and/or analgesics and monitor closely for IRRs and/or CRS.<br>• If symptoms do not resolve to Grade 1 or better for 3 consecutive days, contact Study Chair.

Table. Management Guidelines for Infusion-Related Reactions and Cytokine-Release Syndrome (cont.)

Event	Management
Grade 3 **a**<br>Fever **b** with hypotension requiring a vasopressor (with or without vasopressin)<br>**and/or**<br>Hypoxia requiring	• Permanently discontinue atezolizumab and contact Study Chair**. f**<br>• Administer symptomatic treatment. **c**<br>• For hypotension, administer IV fluid bolus and vasopressor as needed.<br>• Monitor cardiopulmonary and other organ function closely; monitoring in the ICU is recommended. Administer IV fluids as clinically indicated, and manage constitutional symptoms and organ toxicities

high-flow oxygen **d** by nasal cannula, face mask, non-rebreather mask, or venturi mask	as per institutional practice.<br>• Rule out other inflammatory conditions that can mimic CRS (e.g., sepsis). If no improvement within 24 hours, initiate workup and assess for signs and symptoms of HLH or MAS.<br>• Administer IV corticosteroids (e.g., methylprednisolone 2 mg/kg/day or dexamethasone 10 mg every 6 hours).<br>• Consider anti-cytokine therapy. **e**<br>• Hospitalize patient until complete resolution of symptoms. If no improvement within 24 hours, manage as per Grade 4, that is, admit patient to ICU and initiate hemodynamic monitoring, mechanical ventilation, and/or IV fluids and vasopressors as needed; for patients who are refractory to anti-cytokine therapy, experimental treatments may be considered at the discretion of<br>the investigator and in consultation with the Study Chair.
Grade 4 **a**<br>Fever **b** with hypotension requiring multiple vasopressors (excluding vasopressin)<br>**and/or**<br>Hypoxia requiring oxygen by positive pressure (e.g., CPAP, BiPAP, intubation and mechanical ventilation)	• Permanently discontinue atezolizumab and contact Study Chair. **f**<br>• Administer symptomatic treatment. **c**<br>• Admit patient to ICU and initiate hemodynamic monitoring, mechanical ventilation, and/or IV fluids and vasopressors as<br>• needed. Monitor other organ function closely. Manage<br>• constitutional symptoms and organ toxicities as per institutional practice.<br>• Rule out other inflammatory conditions that can mimic CRS<br>• (e.g., sepsis). If no improvement within 24 hours, initiate workup and assess for signs and symptoms of HLH or MAS.<br>• Administer IV corticosteroids (e.g., methylprednisolone 2 mg/kg/day or dexamethasone 10 mg every 6 hours).<br>• Consider anti-cytokine therapy. e For patients who are refractory to anti-cytokine therapy, experimental treatments **g** may be considered at the discretion of the investigator and in consultation with the Study Chair<br>• Hospitalize patient until complete resolution of symptoms

ASTCT =American Society for Transplantation and Cellular Therapy; BiPAP =bi-level positive airway pressure; CAR =chimeric antigen receptor; CPAP =continuous positive airway pressure; CRS =cytokine-release syndrome; CTCAE =Common Terminology Criteria for Adverse Events; eCRF =electronic Case Report Form; HLH =hemophagocytic lymphohistiocytosis; ICU =intensive care unit; IRR =infusion-related reaction; MAS =macrophage activation syndrome; NCCN =National Cancer Comprehensive Network; NCI =National Cancer Institute.

Note: The management guidelines have been adapted from NCCN guidelines for management of CAR T-cell-related toxicities (Version 2.2019).

a Grading system for management guidelines is based on ASTCT consensus grading for CRS. NCI CTCAE (version as specified in the protocol) should be used when reporting severity of IRRs, CRS, or organ toxicities associated with CRS on the Adverse Event eCRF. Organ toxicities associated with CRS should not influence overall CRS grading.

b Fever is defined as temperature ≥ 38°C not attributable to any other cause. In patients who develop CRS and then receive anti-pyretic, anti-cytokine, or corticosteroid therapy, fever is no longer required when subsequently determining event severity (grade). In this case, the grade is driven by the presence of hypotension and/or hypoxia.

c Symptomatic treatment may include oral or IV antihistamines, anti-pyretics, analgesics, bronchodilators, and/or oxygen. For bronchospasm, urticaria, or dyspnea, additional treatment may be administered as per institutional practice.

d Low flow is defined as oxygen delivered at ≤ 6 L/min, and high flow is defined as oxygen delivered at > 6 L/min.

e There are case reports where anti-cytokine therapy has been used for treatment of CRS with immune checkpoint inhibitors (Rotz et al. 2017; Adashek and Feldman 2019), but data are limited, and the role of such treatment in the setting of antibody-associated CRS has not been established.

f Resumption of atezolizumab may be considered in patients who are deriving benefit and have fully recovered from the event. Patients can be re-challenged with atezolizumab only after approval has been documented by both the investigator (or an appropriate delegate) and the Study Chair. For subsequent infusions, administer oral premedication with antihistamines, anti-pyretics, and/or analgesics, and monitor closely for IRRs and/or CRS. Premedication with corticosteroids and extending the infusion time may also be considered after consulting the Study Chair and considering the benefit-risk ratio.

g Refer to Riegler et al. (2019) for information on experimental treatments for CRS.

9.2.10 Neurologic Disorders

Myasthenia gravis and Guillain-Barré syndrome have been observed with single-agent atezolizumab. Patients may present with signs and symptoms of sensory and/or motor neuropathy. Diagnostic workup is essential for an accurate characterization to differentiate between alternative etiologies. Management guidelines for neurologic disorders are provided below.

Management Guidelines for Neurologic Disorders

Event	Management
Immune-mediated neuropathy, Grade 1	• Continue atezolizumab.<br>• Investigate etiology.
Immune-mediated neuropathy, Grade 2	• Withhold atezolizumab for up to 12 weeks after event onset.[a]<br>• Investigate etiology.<br>• Initiate treatment as per institutional guidelines.<br>• If event resolves to Grade 1 or better, resume atezolizumab.[b]<br>• If event does not resolve to Grade 1 or better while withholding atezolizumab, permanently discontinue atezolizumab and contact Study Chair.
Immune-mediated neuropathy, Grade 3 or 4	• Permanently discontinue atezolizumab and contact Study Chair.[c]<br>• Initiate treatment as per institutional guidelines.
Myasthenia gravis and Guillain-Barré syndrome (any grade)	• Permanently discontinue atezolizumab and contact Study Chair.[c]<br>• Refer patient to neurologist.<br>• Initiate treatment as per institutional guidelines.<br>• Consider initiation of corticosteroids equivalent to 1-2 mg/kg/day oral or IV prednisone.

[a] Atezolizumab may be withheld for longer period of time (i.e., >12 weeks after event onset) to allow for corticosteroids (if initiated) to be reduced to the equivalent of ≤ 10 mg/day oral prednisone. The acceptable length of the extended period of time must be agreed upon by the investigator and the Study Chair.

[b] If corticosteroids have been initiated, they must be tapered over ≥ 1 month to the equivalent of ≤ 10 mg/day oral prednisone before ateolizumab can be resumed.

[c] Resumption of atezolizumab may be considered in patients who are deriving benefit and have fully recovered from the immune-mediated event. Patients can be re-challenged with atezolizumab only after approval has been documented by both the investigator (or an appropriate delegate) and the Study Chair.

9.2.11 Immune-mediated Meningoencephalitis

Immune-related meningoencephalitis is an identified risk associated with the administration of atezolizumab. Immune-mediated meningoencephalitis should be suspected in any patient presenting with signs or symptoms suggestive of meningitis or encephalitis, including, but not limited to, headache, neck pain, confusion, seizure, motor or sensory dysfunction, and altered or depressed level of consciousness.

Encephalopathy from metabolic or electrolyte imbalances needs to be distinguished from potential meningoencephalitis resulting from infection (bacterial, viral, or fungal) or progression of malignancy, or secondary to a paraneoplastic process.

All patients being considered for meningoencephalitis should be urgently evaluated with a CT scan and/or MRI scan of the brain to evaluate for metastasis, inflammation, or edema. If deemed safe by the treating physician, a lumbar puncture should be performed and a neurologist should be consulted.

Patients with signs and symptoms of meningoencephalitis, in the absence of an identified alternate etiology, should be treated according to the guidelines below.

Management Guidelines for Immune-Mediated Meningoencephalitis

Event	Management
Immune-mediated meningoencephalitis, all grades	• Permanently discontinue atezolizumab and contact Study Chair.<br>• Refer patient to neurologist.<br>• Initiate treatment with corticosteroids equivalent to 1-2 mg/kg/day IV methylprednisolone and convert to 1-2 mg/kg/day oral prednisone or equivalent upon improvement.<br>• If event does not improve within 48 hours after initiating corticosteroids, consider adding an immunosuppressive agent.<br>• If event resolves to Grade 1 or better, taper corticosteroids over ≥ 1 month.

[a] Resumption of atezolizumab may be considered in patients who are deriving benefit and have fully recovered from the immune-mediated event. Patients can be re-challenged with atezolizumab only after approval has been documented by both investigator (or an appropriate delegate) and the Study Chair.

9.2.12 Immune-Mediated Myositis

Immune-mediated myositis has been associated with the administration of atezolizumab. Myositis or inflammatory myopathies are a group of disorders sharing the common feature of inflammatory muscle injury; dermatomyositis and polymyositis are amongst the most common disorders. Initial diagnosis is based on clinical (muscle weakness, muscle pain, skin rash in dermatomyositis), biochemical (serum creatine-kinase increase), and imaging (electromyography/MRI) features, and is confirmed with a muscle-biopsy. Patients with signs and symptoms of myositis, in the absence of an identified alternate etiology, should be treated according to the guidelines below.

Management guidelines for Immune-Mediated Myositis

Event	Management
Immune-mediated myositis, Grade 1	• Continue atezolizumab<br>• Refer patient to rheumatologist or neurologist.<br>• Initiate treatment as per institutional guidelines
Immune-mediated myositis, Grade 2	• Withhold atezolizumab for up to 12 weeks after event onset[a] and contact Study Chair.<br>• Refer patient to rheumatologist or neurologist.<br>• Initiate treatment as per institutional guidelines .<br>• Consider treatment with corticosteroid equivalent to 1-2 mg/kg/day IV methylprednisolone and convert to 1-2 mg/kg/day oral prednisone or equivalent upon improvement.<br>• If corticosteroids are initiated and event does not improve within 48 hours after initiating corticosteroids, consider adding an immunosuppressive agent.<br>• If event resolves to Grade 1 or better, resume atezolizumab.[b]<br>• If event does not resolve to Grade 1 or better while withholding atezolizumab, permanently discontinue atezolizumab and contact Study Chair.
Immune-mediated myositis, Grade 3	• Withhold atezolizumab for up to 12 weeks after event onset[a] and contact Study Chair.<br>• Refer patient to rheumatologist or neurologist.<br>• Initiate treatment as per institutional guidelines .<br>• Respiratory support may be required in more severe cases .<br>• Initiate treatment with corticosteroids equivalent to 1-2 mg/kg/day IV methylprednisolone or higher-dose bolus if patient is severely compromised (e.g. cardiac or respiratory symptoms, dysphagia, or weakness that severely limits mobility); convert to 1-2 mg/kg/day oral prednisolone or equivalent upon improvement.<br>• If event does not improve within 48 hours after initiating corticosteroids, consider adding an immunosuppressive agent.<br>• If event resolves to Grade 1 or better, resume atezolizumab.[b]<br>• If event does not resolve to Grade 1 or better while withholding atezolizumab, permanently discontinue atezolizumab and contact Study Chair.[c]<br>• For recurrent events, treat as a Grade 4 event.

Event	Management
Immune-mediated myositis, Grade 4	• Permanently discontinue atezolizumab and contact Study Chair.[c]<br>• Refer patient to rheumatologist or neurologist.<br>• Initiate treatment as per institutional guidelines. Respiratory support may be required in more severe cases.<br>• Initiate treatment with corticosteroids equivalent to 1-2 mg/kg/day IV methylprednisolone or higher-dose bolus if patient is severely compromised (e.g. cardiac or respiratory symptoms, dysphagia, or weakness that severely limiting mobility); convert to 1-2 mg/kg/day oral prednisone or equivalent upon improvement.<br>• If event does not improve within 48 hours after initiating corticosteroids, consider adding an immunosuppressive agent.<br>• If event resolves to Grade 1 or better, taper corticosteroids over ≥ 1 month.

[a] Atezolizumab may be withheld for longer period of time (i.e., >12 weeks after event onset) to allow for corticosteroids (if initiated) to be reduced to the equivalent of ≤ 10 mg/day oral prednisone. The acceptable length of the extended period of time must be agreed upon by the investigator and the Study Chair.
[b] If corticosteroids have been initiated, they must be tapered over ≥ 1 month to the equivalent of ≤ 10 mg/day oral prednisone before ateolizumab can be resumed.
[c] Resumption of atezolizumab may be considered in patients who are deriving benefit and have fully recovered from the immune-mediated event. Patients can be re-challenged with atezolizumab only after approval has been documented by both the investigator (or an appropriate delegate) and the Study Chair.

9.2.13 Severe Cutaneous Adverse Reactions (SCARs)

Severe Cutaneous Adverse Reactions (SCARs) are rare but potentially fatal skin toxicities frequently associated with drug use including immune checkpoint inhibitor as a class. A comprehensive analysis of the data available across the TECENTRIQ® (atezolizumab) program has identified cases of SCARs following atezolizumab use. SCARs were previously a potential risk of atezolizumab, and have been monitored continuously.
Based upon the totality of evidence in a recent analysis, SCARs are now considered to be an identified risk for atezolizumab.
SCARs are a heterogeneous group of immunologically mediated skin eruptions.
Although **rare, these events are potentially fatal**, and mainly constituted by **erythema multiforme, acute generalised exanthematous pustulosis, Stevens-Johnson syndrome (SJS), Toxic Epidermal Necrolysis (TEN) and drug rash with eosinophilia and systemic symptoms (DRESS).**
As per epidemiology data, the incidence of SJS and TEN ranges from 0.8 to 5.3 and 1.2 to 6 per million person-years respectively (19, 20)

A cumulative analysis of the company safety database across the TECENTRIQ (atezolizumab) program identified at total of 99 cases of SCARs, of which 36 were confirmed by histopathology or specialist diagnosis, in patients who have received TECENTRIQ (atezolizumab). Approximately 23,654 clinical trial patients and 106,316 patients in post-marketing settings have been exposed to TECENTRIQ (atezolizumab) as of 17 May 2020. The incidence rates of SCAR, regardless of severity, from pooled atezolizumab monotherapy (N=3178) and combination therapy (N=4371) companysponsored clinical studies were 0.7% and 0.6% respectively. One fatal case of TEN was reported in a 77 year old female patient who received atezolizumab monotherapy.

Management guidelines for SCARs

- For suspected SCARs the patients should be referred to a dermatologist for further diagnosis and management
- Atezolizumab should be withheld for patients with suspected SJS or TEN
- Atezolizumab should be permanently withdrawn for any grade confirmed SJS or TEN
- Caution should be used when considering the use of atezolizumab in a patient who has previously experienced a severe or life-threatening skin adverse reaction on prior treatment with other immune-stimulatory anticancer agents

9.2.14 Overdose

In clinical trials with atezolizumab, doses of up to 20 mg/kg have been administered. The standard fixed dose of 1200 mg is equivalent to 15 mg/kg in a typical patient. No dose-limiting toxicities were observed at the 20 mg/kg dose level and the incidence and intensity of AEs reported have not been shown to be dependent on dose. A maximum tolerated dose has not been established.

Atezolizumab is supplied in vials containing the fixed 1200 mg dose of drug substance, which minimizes the chance of overdose. Because the dose per patient is not based on weight or body surface area, and no dose calculation is required, the risk of overdose due to medication errors is minimal.

The data available to date suggest that the potential for harm from overdose is very low.

9.3 Anticipated Toxicities of Carboplatin and Dose Modifications

Carboplatin is known to cause bone marrow suppression including myelosuppression, anemia, and thrombocytopenia. Carboplatin-based chemotherapy is considered to be moderately emetogenic. Patients will be monitored for carboplatin-related adverse events.

9.3.1 Hematologic Toxicity

At the start of each cycle if the patient is receiving carboplatin, the ANC must be ≥1000/mm^3 and the platelet count must be ≥ 80,000/mm^3. Platelets must be ≥ 100,000 for study entry. Growth factors may be used in accordance with American Society of Clinical Oncology (ASCO) and National Comprehensive Cancer Network (NCCN) guidelines (Smith et al. 2006; NCCN 2012). Upon recovery, dose adjustments at the start of a subsequent cycle will be made on the basis of the lowest platelet and
neutrophil values from the previous cycle (see Table below).

9.3.1.1 Carboplatin Dose Modification for Hematologic Toxicities

**Toxicity**	**Carboplatin Dose**
ANC <500/mm^3 and platelets ≥ 50,000/mm^3	Decrease AUC by 1
Platelets < 50,000/mm^3, regardless of ANC	Decrease AUC by 1
Platelets < 50,000/mm^3 with Grade ≥ 2 bleeding, regardless of ANC	Decrease AUC by 2
ANC < 1000/mm^3 plus fever of ≥ 38.5°C	Decrease AUC by 1

All dose reductions for the first episode of neutropenic fever or thrombocytopenia (platelet count < 25,000 or < 50,000 with bleeding or that requires transfusion) are permanent. If a second episode of neutropenic fever or thrombocytopenia requiring dose reduction occurs, the dose of carboplatin will be reduced according to physician judgment and local standard practice. Colony-stimulating factors, such as granulocyte colony-stimulating factor, may be used instead of dose reduction for neutropenic fever or Grade 4 neutropenia, according to the local standard practice and ASCO guideline. Patients who require a third dose reduction will immediately discontinue chemotherapy.

In the event that dose adjustments are needed for both ANC and platelets, patients are to receive the lower dose.
For Grade 3 ANC toxicity (ANC <1000-500/ml)**,** carboplatin should be withheld until the patient recovers completely or to Grade 1 toxicity. The treatment should then be resumed at 50% dose (permanent dose reduction). If recovery to Grade 1 toxicity does not occur within 3 weeks, the patient's chemotherapy will be discontinued.

Treatment with carboplatin can be delayed for up to 3 weeks until the Day 1 ANC is ≥1000/mm^3 and the platelet count is ≥ 80,000/mm^3. However, if the counts have not recovered in 3 weeks, the patient's chemotherapy will be dose-reduced, held until adequate neutrophil recovery, or

discontinued, according to physician judgment and local standard practice. If chemotherapy is held longer than 42 days, all study treatment should be discontinued.

Investigators should be vigilant and alert to early and overt signs of myelosuppression, infection, or febrile neutropenia so that these complications can be promptly and appropriately managed. Patients should be made aware of these signs and encouraged to seek medical attention at the earliest opportunity. If chemotherapy must be withheld because of hematologic toxicity, full blood counts (including differential WBC) should be obtained weekly until the counts reach the lower limits for treatment as outlined. The treatment schedule will then proceed in the usual sequence.

No dose reductions will be made for anemia. Patients should be supported per the treating physician's institution's guidelines.

9.3.2 Non-Hematologic Toxicity

For Grade 3 or 4 non-hematologic toxicities, treatment should be delayed until resolution to less than or equal to the patient's baseline value. Dose reductions at the start of the subsequent cycle will be made on the basis of non-hematologic toxicities from the dose administered in the preceding cycle. The table below provides the relevant dose adjustments for non-hematologic toxicities.

9.3.2.1 Carboplatin Dose Modification on the Basis of Non-Hematologic

Toxicities in the Preceding Cycle

**Toxicity**	**Adjusted Carboplatin Dose[a]**
Diarrhea	Grade 3 or 4[b]: 100% of dose
Oral mucositis	Grade 3 or 4: Decrease AUC by 1
Nausea/Vomiting	Grade 3 or 4: Decrease AUC by 1
Neurotoxicity (motor or sensory)	Grade 2: 100% of dose<br>Grade 3 or 4: Decrease AUC by 1
Transaminase Elevation	Grade 3: Decrease AUC by 1<br>Grade 4: discontinue
Other	Grade 3 or 4: Decrease AUC by 1

AUC area under the concentration curve.

a If deemed appropriate by the treating physician, adjust carboplatin dose to the specified percentage of the previous AUC.
b Or any grade of diarrhea requiring hospitalization.

Nausea and/or vomiting should be controlled with adequate anti-emetics. If Grade 3 or 4 nausea/vomiting occurs in spite of anti-emetics, the dose should be reduced by 25% for the next course. If tolerated, the dose should be increased back to 100% as soon as possible.

If, on Day 1 of any treatment cycle, the patient has grade 2 oral mucositis, the treatment can be withheld until the oral mucositis is cleared. If the oral mucositis has not cleared in 3 weeks, the patient's chemotherapy will be discontinued. If acute Grade 3 oral mucositis occurs at any time, the dose should be given at a 75% dose when the oral mucositis is completely cleared. This is a permanent dose reduction.

9.3.3

9.4 Special Considerations

- For toxicities which are considered by the treating investigator unlikely to develop into serious or life–threatening events (e.g. alopecia, altered taste etc.), treatment may be continued at the same dose without reduction or interruption.
- The treating investigator may reduce a subject's dose for a toxicity of any grade/duration where s/he believes it to be in the best interests of the subject.
- Any consideration to modification of the above dose modification guidelines should be discussed with the Principal Investigator for approval or disapproval in advance.

10. DRUG FORMULATION/STORAGE/SUPPLY

10.1 Atezolizumab (MPDL3280A)

Atezolizumab is an investigational agent and will be supplied free-of-charge from Genentech.

10.1.1 Formulation

The atezolizumab drug product is typically provided in a single-use 15 mL or 20 mL USP/Ph. Eur. Type 1 glass vial as a colorless-to-slightly-yellow, sterile, preservative-free clear liquid solution intended for IV administration. Other excipients of the atezolizumab drug product include histidine acetate, sucrose and polysorbate 20.

Atezolizumab must be refrigerated at 2°C – 8°C (36°F – 46°F) upon receipt until use. Atezolizumab vials should not be used beyond the expiration date provided by the manufacturer. No preservative is used in the atezolizumab drug product or the diluent; therefore, each vial is intended for single use only. Discard any unused portion of drug left in a vial. Vial contents should not be frozen or shaken and should be protected from direct sunlight. Once prepared, atezolizumab can be stored for up to 8 hours at room temperature.

For further details, see the atezolizumab Investigator's Brochure.

10.1.2 Dosage, Administration, and Storage

The dose level of atezolizumab to be tested in this study is 1200 mg (equivalent to an average body weight–based dose of 15 mg/kg) administered by IV infusion every 3 weeks (21 [± 2] days). Atezolizumab will be delivered in infusion bags with IV infusion lines that have product contacting surfaces of polyvinyl chloride (PVC) or polyolefin and 0.2 μm in-line filters (filter membrane of polyethersulfone [PES]). No incompatibilities have been observed between atezolizumab and PVC or polyolefin infusion materials (bags or infusion lines).

Administration of atezolizumab will be performed in a setting with emergency medical facilities and staff who are trained to monitor for and respond to medical emergencies.

The initial dose of atezolizumab will be delivered over 60 (± 15) minutes. If the first infusion is tolerated without infusion-associated AEs, the second infusion may be delivered over 30 (± 10) minutes. If the 30-minute infusion is well tolerated, all subsequent infusions may be delivered over 30 (± 10) minutes. For the first infusion, the patient's vital signs (heart rate, respiratory rate, blood pressure, and temperature) should be determined within 60 minutes before, during (every 15 [± 5] minutes), and 30 (± 10) minutes after the infusion. For subsequent infusions, vital signs will be collected within 60 minutes before and within 30 minutes after the infusion. Vital signs should be collected during the infusion only if clinically indicated. Patients will be informed about the possibility of delayed post-infusion symptoms and instructed to contact their study physician if they develop such symptoms. **Atezolizumab should be administered prior to carboplatin.**

No premedication will be allowed for the first dose of atezolizumab. Premedication may be administered for Cycles ≥ 2 at the discretion of the treating physician. The management of infusion-related reactions (IRRs) will be according to severity as follows:

- In the event that a patient experiences a mild (NCI CTCAE Grade 1) IRR, the infusion rate should be reduced to half the rate being given at the time of event onset. Once the event has resolved, the investigator should wait for 30 minutes while delivering the infusion at the reduced rate. If tolerated, the infusion rate may then be increased to the original rate.
- In the event that a patient experiences a moderate IRR (NCI CTCAE Grade 2) or flushing, fever, or throat pain, the infusion should be immediately interrupted and the patient should receive aggressive symptomatic treatment. The infusion should be restarted only after the symptoms have adequately resolved to baseline grade. The infusion rate at restart should be half of the infusion rate that was in progress at the time of the onset of the IRR.
- For severe or life-threatening IRRs (NCI CTCAE Grade 3 or 4), the infusion should be stopped immediately, and aggressive resuscitation and supportive measures should be initiated. Patients experiencing severe or life-threatening IRRs will not receive further infusion and will be further managed as clinically indicated until the event resolves.

For anaphylaxis precautions, see the appendices.

Guidelines for dosage modification, treatment interruption, or discontinuation and the management of specific adverse events are provided in the protocol.

10.2 Carboplatin

Carboplatin is commercially available. Carboplatin should be administered intravenously according to local practice. **Carboplatin should be administered after atezolizumab if the patient is receving atezolizumab.**

The carboplatin dose will be calculated using the Cockcroft and Gault formula.

For the purposes of this protocol, the GFR is considered to be equivalent to the creatinine clearance (CrCl). The CrCl is calculated by institutional guidelines or by the method of Cockcroft and Gault (1976) using the following formula:

$$CrCl = \frac{(140-age)\,(wt)}{72 \times Scr} \quad (\times\ 0.85 \text{ if female})$$

Where: CrCl = creatinine clearance in mL/min
age = patient's age in years
wt = patient’s weight in kg
Scr = serum creatinine in mg/dL

If a patient’s GFR is estimated on the basis of serum creatinine measurements by the isotope dilution mass spectroscopy method, the U.S. Food and Drug Administration (FDA) recommends that physicians consider capping the dose of carboplatin for desired

exposure (AUC) to avoid potential toxicity caused by overdosing.

The maximum dose is based on a GFR estimate that is capped at 125 mL/min for patients with normal renal function. No higher estimated GFR values should be used.

For a target AUC = 6, the maximum dose is 6 × 150 = 900 mg.
For a target AUC = 5, the maximum dose is 5 × 150 = 750 mg.
For a target AUC = 4, the maximum dose is 4 × 150 = 600 mg.

See the FDA's communication regarding carboplatin dosing for more details at
http://www.fda.gov/AboutFDA/CentersOffices/OfficeofMedicalProductsandTobacco/CDER/ucm228974.htm

10.3 Drug Accountability

Accountability for the drug at all study sites is the responsibility of the site Principal Investigator and designated Pharmacy representative. The investigator will ensure that the investigational drug is used only in accordance with this protocol. Drug accountability records indicating the drug's delivery date to the site, inventory at the site, use by each patient, and destruction locally will be maintained by the clinical site. Atezolizumab should be destroyed locally as per local policy. Accountability records will include dates, quantities, lot numbers, expiration dates, and patient numbers. These records should be sent to Genentech for filing.

11. CORRELATIVE/SPECIAL STUDIES

11.1 Archived Tissue Samples

Metastatic tissue is the preferred sample; however, if the remaining tissue is insufficient to fit the requirements below, tissue from the patient's primary tumor is acceptable.

Formalin-fixed paraffin embedded tissue blocks from the patient's metastatic lesion collected for diagnostic and therapeutic purposes is requested for all patients enrolled in the study at the time of registration. They will be returned within 8 weeks (or earlier if requested); tissue taken from these blocks will not be returned.

In case the participating institutions are not allowed to release clinical paraffin embedded blocks from prior surgeries/biopsies, ideally we will need the following cut in this order:

A. If sending prior metastatic biopsy (performed pre-enrollment for diagnostic purposes) then send:

1. Six (6) 4 µm unstained sections on charged slides (levels 1,6 for immune infiltrate assessment, levels 2-5 to Genentech for PDL-1 IHC)
2. Six (6) 10 µm unstained sections on non-charged slides (Vanderbilt DNA/RNA isolation)
3. Twelve (12) 4 µm unstained slides on charged slides (level 1 and 12 to be H&E stained upon receipt)

If remaining tissue is insufficient for the complete request, please send:

1. Six (6) 4 µm unstained sections on charged slides (levels 1,6 for immune infiltrate assessment, levels 2-5 to Genentech for PDL-1 IHC)
2. Two 5 x 10 µm peels in a DNAse-free/RNAse-free tube

If remaining tissue is very limited, please send:

Six (6) 4 µm unstained sections on charged slides (levels 1,6 for immune infiltrate assessment, levels 2,3,4,5 to Genentech for PDL-1 IHC)

B. If sending primary tumor send:

1. Six (6) 4 µm unstained sections on charged slides (levels 1,6 for immune infiltrate assessment, levels 2-5 to Genentech for PDL-1 IHC)
2. Six (6) 10 µm unstained sections on non-charged slides (Vanderbilt DNA/RNA isolation)
3. Two (2) 4 µm unstained slides on charged slides (last will be H&E stained upon receipt)

If remaining tissue is insufficient for the complete request, please send:

1. Six (6) 4 µm unstained sections on charged slides (levels 1,6 for immune infiltrate assessment, levels 2-5 to Genentech for PDL-1 IHC)

If remaining tissue is very limited, please send:

Six (6) 4 µm unstained sections on charged slides (levels 1,6 for immune infiltrate assessment, levels 2,3,4,5 to Genentech for PDL-1 IHC)

After removing all stray tissue from the area, the microtome blade and surrounding area should be wiped with 70% ETOH before starting to section the block and between patients to prevent contamination with extraneous tissue.

All formalin-fixed paraffin embedded tissue and/or slides should be sent with a cold pack to avoid melting that could damage tissue analysis.

Once a patient meets all other eligibility, and registers for the study, **collection of tissue from a metastatic site, if reasonably safe, is also a requirement for entry to this study. Pre-trial and post-progression biopsies should be collected.** Research tissue blocks prepared from these metastatic biopsies will not be returned. **All patients must have a biopsy of a metastatic site (if reasonably safe) at the end of treatment if the patient is removed for disease progression, or if on the carboplatin only arm and crossing over to atezolizumab.**

11.1.1 Guidelines for Tissue Acquisition on Biopsies of Metastatic Lesions

Tissue specimens, when feasible, will be collected from recurrent or metastatic lesions using standard institutional procedures. The amount of tissue collected will follow the guidelines listed below. If a patient has more than one site of disease, only one site needs to be biopsied, and the site is left to the discretion of the patient and the treating physician. If a patient is undergoing resection of a lesion for clinical reasons (i.e. wedge resection of a new lung lesion for confirmation of diagnosis or re-testing of hormone receptor or HER2 status; or, resection of a chest wall lesion; or, resection of a lymph node), then the patient may opt to have a portion of that tissue (roughly equivalent to the goal amount of tissue listed in the guidelines above, i.e. the equivalent of two 5-mm punch biopsies of the skin, or 3-6 18-gauge core biopsies) stored for research at the time of the procedure.

Listed below are the goal amounts of tissue for patients who undergo core biopsy or punch biopsy, or who have either ascites fluid or pleural fluid accessible for collection. Please note that the below are guidelines for the amount of tissue to be obtained at the baseline biopsy, and are not meant to replace clinical judgment at the time the procedure is performed. Less than the goal quantity of tissue is accepted for each type of biopsy, and will be left to the clinical judgment of the physician performing the procedure.

- Breast: A goal of 3-6 core biopsy specimens will be obtained using standard institutional guidelines for a diagnostic core biopsy of a breast mass
- Skin/chest wall: A goal of one to two 5-mm punch biops(ies)
- Lymph node: A goal of 3-6 core biopsy specimens will be obtained using an 18-gauge needle
- Liver: A goal of 3-6 core biopsy specimens will be obtained using an 18-gauge needle
- Lung: A goal of 3 core biopsy specimens will be obtained using an 18-gauge needle. Due to the risk of pneumothorax associated with core needle biopsies of lung nodules, peripheral lesions should be carefully selected.

- Bone: Because the yield of malignant tissue from bone biopsies tends to be relatively low, if a patient has another accessible site of disease (i.e. skin, lymph node, liver), that site should be biopsied preferentially. If bone is the only biopsy-accessible site, then a goal of 3-6 core biopsy specimens will be obtained using an 18-gauge needle.
- Pleural Fluid (known or suspected to be due to malignancy): This should only be used if there is no other site noted above that can be biopsied and this should be discussed with the VICC coordinating center. A goal of 500 cc of pleural fluid will be obtained with a standard thoracentesis procedure, with or without image guidance, according to the clinical judgment of the treating physician and clinician performing the procedure. Less than the goal amount is acceptable, and should be based upon the clinical judgment of the Investigator and the clinician performing the procedure. If more than the goal amount of fluid is obtained, then the entire specimen (with the exception of what is needed for clinical purposes, if applicable) will be stored at Vanderbilt.
- Ascites fluid: This should only be used if there is no other site noted above that can be biopsied and this should be verified by the VICC coordinating center. A goal of 500 cc of ascites fluid will be obtained with a standard paracentesis procedure, with or without image guidance, according to the clinical judgment of the treating physician and clinician performing the procedure. Less than the goal amount is acceptable, and should be based upon the clinical judgment of the Investigator and the clinician performing the procedure.

11.1.2 Instructions on fresh tissue specimen handling

Tissue obtained at Vanderbilt will be handled differently as it does not need to be shipped. Please see the Vanderbilt lab manual for handing if obtaining a biopsy at Vanderbilt

- First core biopsy should be suspended in 10% buffered formalin.
- Second core biopsy should be suspended in 5-10 volumes “RNAlater” solution*
- Third core biopsy should be suspended in 10% buffered formalin.
- Fourth core biopsy should be suspended in 5-10 volumes “RNAlater” solution*
- Fifth core biopsy should be suspended in 10% buffered formalin

*RNAlater kits will be provided. 1 core should be placed in one 1.5ml specimen tube. All samples should be shipped immediately, according to the lab manual.

For patients in whom a core biopsy is not possible and who thus undergo fine needle aspiration (FNA), 3 passes should be collected.

- First pass should be evacuated and rinsed directly into 10% buffered formalin
- Second pass should be evacuated and rinsed directly into 5-10 volumes “RNAlater” solution*
- Last pass should be evacuated and rinsed directly into 10% buffered formalin.

11.2 Blood/plasma collection

Streck Cell-Free DNA BCT kits will be provided for circulating tumor DNA. One vial of whole blood should be collected in a Cell-Free DNA BCT tube (Streck tube). Fill the tube completely. Remove the tube from the adapter and immediately mix by gentle inversion 8 to 10 times. Collection times are: cycle 1/day 1, cycle 2/day 1, and end-of-treatment, see study calendar.

Tube should be packed and shipped according to the lab manual and sent immediately (within 24 hours) to VICC at the address provided. **Do not freeze specimens collected in Streck tube.**

11.3 Tissue specimen and blood/plasma labeling and documentation

Label each collection containers with Patient ID sequence number/code letter, site and location of biopsy, date and time.

If sample comes in contact with contaminate, make note in information section of paperwork.

Enter time core biopsy was collected on paperwork.

Outside sites should ship specimens along with Tissue Registration Form as directed in the lab manual.

The specimens will be logged in as a consented specimen and available for molecular pathology studies.

11.4 Peripheral Blood Mononuclear Cell Collection

Blood will be collected on Cycle 1/ Day 1, Cycle 2/ Day 1, and at the end-of-treatment (also Cycle 1/Day 1, Cycle 2/Day 1, and end-of-treatment for those crossing over to carboplatin + atezolizumab).

BD Vacutainer® CPT™ Mononuclear Cell Preparation Tubes are included to collect blood. One vial of whole blood should be collected in a BD Vacutainer® CPT™ (CPT tube).
Whole blood is drawn directly into the CPT™ using standard phlebotomy techniques and in accordance with the lab manual. Samples should be shipped according to the lab manaul.

11.5 Genetic Testing

Participants will be given information as part of the informed consent process that samples will be used for research tests that will include genetic studies and testing. The intent is not to give participants (or his/her medical providers) the results of any testing done for research purposes; however, incidental germline (heritable) mutations may be identified of which a participant may or may not already be aware. In the case that an incidental genetic finding is identified, the Protocol Chair of this project will be notified. The possible decisions for handling incidental findings may include notification of the participant (and provider); recommendation for genetic counseling, which may or may not include genetic testing (e.g., if the finding was not done in a CLIA certified laboratory); or, neither. In general, a member of the participant's treating team will be given the information to help with notification. In all cases, the current policy of the Vanderbilt and

local/participating site IRB, as applicable, will be followed and any additional approvals that may be required prior to participant notification will be secured in advance.

11.6 Specimen Banking

Any leftover study tissue or blood samples may be stored for future research studies. The subjects will consent to the future use of samples in the consent form for the study, which includes the transfer of specimens to Genentech for product development. The study PI and collaborators have approval by the TBCRC, which has custodial oversight of all biospecimens collected as part of a TBCRC trial, to address the research questions described in the protocol document. All future use as part of residual or repository specimens collected in this trial for purposes not prospectively defined will require review and approval by the TBCRC according to its established policies, whether the specimens are stored in a central site or at a local institution or in a virtual repository. Secondary use of bio-specimens for new endpoints must be submitted to the TBCRC Central Office for possible review by the TBCRC Correlative Science Review Committee.

11.7 Biopsy Questionnaire

A questionnaire will be given to each participant to complete at cycle 2, day 1. This questionnaire is designed to assess the impact of the biopsy on the patient's quality of life and overall experience with the study.

11.8 Imaging Correlative Study

Evaluation of muscle mass, muscle attenuation, and adipose mass will be determined using baseline abdominal CT scans collected for central review. A transverse cut at the L3 level will be extracted, as muscle and adipose area at this level is linearly related to whole body mass[16]. SliceOmatic software (version 5, Tomovision) will be used to process images, providing a highly accurate estimation of cross-sectional skeletal muscle area and muscle attenuation with high inter-observer reliability[17]. The software is semi-automated and will be run by two independent investigators, with a subset of images read by both investigators to determine a coefficient of variation. Muscle mass, muscle attenuation, and adiposity will be evaluated both as continuous and categorical variables (LMM: yes/no, LMA: yes/no, adipose: tertiles). LMM is defined as skeletal muscle index (SMI, lean muscle area/height, cm^2/m^2) less than 41 and LMA will be defined as average muscle density less than 25 HU, or less than 33 HU if the patient is overweight/obese by BMI. These cut-points are based on previously determined thresholds associated with reduced survival in patients with metastatic solid tumors[18]. We will also explore whether there are alternate cut-points predictive of survival in this particular population based on optimal stratification. Total adiposity will be defined as the sum of visceral adipose tissue, subcutaneous adipose tissue, and intramuscular adipose tissue and evaluated by tertiles.

12. MEASUREMENT OF EFFECT

For the purposes of this study, patients should be re-evaluated for response after every 3 cycles. Response and progression will be evaluated in this study using the international criteria proposed by the revised Response Evaluation Criteria in Solid Tumors (RECIST) Committee (version 1.1). Any evaluable or measurable disease must be documented at screening and reassessed at each subsequent tumor evaluation. For PFS analysis, patients will be considered to have disease progression if their tumor assseessment shows PD by RECIST. However, as conventional response

criteria may not adequately assess the activity of immunotherapeutic agents because radiographic progressive disease does not necessarily reflect therapeutic failure, for patients on the carboplatin + atezolizumab arm, if a patient is found to have disease progression by RECIST, at the discretion of the treating physician, the patient may remain on study and managed by irRECIST (see section 12.5) provided he/she meets the following criteria:

- Evidence of clinical benefit as assessed by the investigator.
- Absence of symptoms and signs (including worsening laboratory values [e.g., new or worsening hypercalcemia]) indicating clinically significant progression of disase
- No decline in ECOG performance status that can be attributed to disease progression
- Absence of tumor progression at critical anatomical sites (e.g., leptomeningeal disease) and no other signs/symptoms of unequivocal disease progression. This includes tumor progression that cannot be managed by protocol-allowed medical interventions (ie., pain secondary to disease or unmanageable ascites, etc.), as determined by the investigator after an integrated review of radiographic data and clinical status.

Scans should be repeated after 6 weeks, and if progression is confirmed by irRECIST, the patient should be removed from study. If scans by irRECIST show the patient to not have disease progression, the patient may remain on study after discussion with the Protocol Chair. Patients on the carboplatin alone arm who show progression by RECIST must either be removed from study or cross over to receive atezolizumab.

Conventional response criteria may not adequately assess the activity of immunotherapeutic agents because radiographic progressive disease does not necessarily reflect therapeutic failure. In Phase Ia Study PCD4989g, several patients who progressed per RECIST v1.1 continued on atezolizumab treatment and subsequently demonstrated durable anti-tumor activity. Of the 21 patients with TNBC in the study who were evaluable for response 3 patients with IC2/3 TNBC showed evidence of target lesion shrinkage after an initial increase in tumor burden or following the appearance of new lesions. These patients likely experienced pseudoprogression or delayed response (Emens et al. 2014). Additionally, in some responding patients with NSCLC in Study PCD4989g, the growth of known lesions or the appearance of new radiographic lesions were shown to contain immune cells and no viable cancer cells on biopsy (Gettinger et al. 2013). The expected rate of pseudoprogression or delayed anti-tumor immunity when atezolizumab is administered in combination with carboplatin is not known. On the other hand, radiographic progression when carboplatin is given alone is likely to represent true disease progression requiring a change in therapy. Although the primary endpoint measures of efficacy (PFS) will be performed using RECIST v1.1, noncomparative analyses of these measures will be performed with use of immune-modified RECIST for patients who continue treatment beyond radiographic progression. The immune-modified RECIST allows the incorporation of new lesions into the calculation of total tumor burden after baseline.

12.1 Definitions

Evaluable for toxicity. All patients will be evaluable for toxicity from the time of their first treatment with atezolizumab and/or carboplatin. Evaluable for objective response. Only those patients who have measurable disease present at baseline, have received at least one cycle of therapy, and have had their disease re-evaluated will be considered evaluable for response. These patients will have their response classified according to the definitions stated below. (Note: Patients who exhibit objective disease progression prior to the end of cycle 1 will also be considered evaluable.)

12.2 Disease Parameters

12.2.1 Measurable

12.2.1.1 Tumor lesions: Must be accurately measured in at least one dimension (longest diameter in the plane of measurement is to be recorded) with a minimum size of:

- 10mm by CT scan (CT scan slice thickness no greater than 5 mm).
- 10mm caliper measurement by clinical exam (lesions which cannot be accurately measured with calipers should be recorded as non-measurable).
- 20mm by chest X-ray.

12.2.1.2 Malignant lymph nodes: To be considered pathologically enlarged and measurable, a lymph node must be ≥15mm in short axis when assessed by CT scan (CT scan slice thickness recommended to be no greater than 5 mm). At baseline and in follow-up, only the short axis will be measured and followed.

12.2.2 Non-measurable

All other lesions, including small lesions (longest diameter <10mm or pathological lymph nodes with ≥10 to <15mm short axis) as well as truly non-measurable lesions. Lesions considered truly non-measurable include: leptomeningeal disease, ascites, pleural or pericardial effusion, inflammatory breast disease, lymphangitic involvement of skin or lung, abdominal masses/abdominal organomegaly identified by physical exam that is not measurable by reproducible imaging techniques.

12.2.3 Special considerations regarding lesion measurability

Bone lesions, cystic lesions, and lesions previously treated with local therapy require particular comment:

12.2.3.1 Bone lesions:

- Bone scan, PET scan or plain films are not considered adequate imaging techniques to measure bone lesions. However, these techniques can be used to confirm the presence or disappearance of bone lesions.
- Lytic bone lesions or mixed lytic-blastic lesions, with identifiable soft tissue components, that can be evaluated by cross-sectional imaging techniques such as

CTor MRI can be considered as measurable lesions if the soft tissue component meets the definition of measurability described above.

- Blastic bone lesions are non-measurable.

12.2.3.2 Cystic lesions:

- Lesions that meet the criteria for radiographically defined simple cysts should not be considered as malignant lesions (neither measurable nor non-measurable) since they are, by definition, simple cysts.
- 'Cystic lesions' thought to represent cystic metastases can be considered as measurable lesions, if they meet the definition of measurability described above. However, if non cystic lesions are present in the same patient, these are preferred for selection as target lesions.

Lesions with prior local treatment:

- Tumor lesions situated in a previously irradiated area, or in an area subjected to other loco-regional therapy, are usually not considered measurable unless there has been demonstrated active tumor.
- Progression in the lesion. Study protocols should detail the conditions under which such lesions would be considered measurable.

12.2.4 Specifications by methods of measurements

12.2.4.1 Measurement of lesions

All measurements should be recorded in metric notation, using calipers if clinically assessed. All baseline evaluations should be performed as close as possible to the treatment start and never more than 21 days before the beginning of the treatment.

12.2.4.2 Method of assessment

The same method of assessment and the same technique should be used to characterise each identified and reported lesion at baseline and during follow-up. Imaging based evaluation should always be done rather than clinical examination unless the lesion(s) being followed cannot be imaged but are assessable by clinical exam.

Clinical lesions: Clinical lesions will only be considered measurable when they are superficial and ≥10mm diameter as assessed using calipers (e.g., skin nodules). For the case of skin lesions, documentation by colour photography including a ruler to estimate the size of the lesion is suggested. When lesions can be evaluated by both clinical exam and imaging, imaging evaluation should be undertaken since it is more objective and may also be reviewed at the end of the study.

Chest X-ray: Chest CT is preferred over chest X-ray, particularly when progression is an important endpoint, since CT is more sensitive than X-ray, particularly in identifying new lesions. However, lesions on chest X-ray may be considered measurable if they are clearly defined and surrounded by aerated lung.

CT, MRI: CT is the best currently available and reproducible method to measure lesions selected for response assessment. Guidelines have defined measurability of lesions on CT scan based on the assumption that CT slice thickness is 5mm or less. When CT scans have slice thickness greater than 5 mm, the minimum size for a measurable lesion should be twice the slice thickness.
MRI is also acceptable in certain situations (e.g. for bodyscans).

Ultrasound: Ultrasound is not useful in assessment of lesion size and should not be used as a method of measurement. Ultrasound examinations cannot be reproduced in their entirety for independent review at a later date and, because they are operator dependent, it cannot be guaranteed that the same technique and measurements will be taken from one assessment to the next. If new lesions are identified by ultrasound in the course of the study, confirmation by CT or MRI is advised. If there is concern about radiation exposure at CT, MRI may be used instead of CT in selected instances.

PET/CT may not be used for RECIST or irRECIST measurements.

Endoscopy, laparoscopy: The utilisation of these techniques for objective tumor evaluation is not advised. However, they can be useful to confirm complete pathological response when biopsies are obtained or to determine relapse in trials where recurrence following complete response or surgical resection is an endpoint.

Tumor markers: tumor markers alone cannot be used to assess objective tumor response. If markers are initially above the upper normal limit, however, they must normalise for a patient to be considered in complete response.

Cytology, histology: These techniques can be used to differentiate between PR and CR in rare cases if required by protocol (for example, residual lesions in tumor types such as germ cell tumors, where known residual benign tumors can remain). When effusions are known to be a potential adverse effect of treatment (e.g. with certain taxane compounds or angiogenesis inhibitors), the cytological confirmation of the neoplastic origin of any effusion that appears or worsens during treatment can be considered if the measurable tumor has met criteria for response or stable disease in order to differentiate between response (or stable disease) and progressive disease.

12.3 Response Criteria

12.3.1 Target lesions

When more than one measurable lesion is present at baseline all lesions up to a maximum of five lesions total (and a maximum of two lesions per organ) representative of all involved organs should be identified as **target lesions** and will be recorded and measured at baseline (this means in instances where patients have only one or two organ sites involved a maximum of two and four lesions respectively will be recorded).

12.3.2 Evaluation of Target Lesions

12.3.2.1 Complete Response (CR): Disappearance of all target lesions. Any pathological lymph nodes (whether target or non-target) must have reduction in short axis to <10 mm.

12.3.2.2 Partial Response (PR): At least a 30% decrease in the sum of diameters of target lesions, taking as reference the baseline sum diameters.

12.3.2.3 Progressive Disease (PD): At least a 20% increase in the sum of diameters of target lesions, taking as reference the smallest sum on study (this includes the baseline sum if that is the smallest on study). In addition to the relative increase of 20%, the sum must also demonstrate an absolute increase of at least 5 mm. (Note: the appearance of one or more new lesions is also considered progression).

12.3.2.4 Stable Disease (SD): Neither sufficient shrinkage to qualify for PR nor sufficient increase to qualify for PD, taking as reference the smallest sum of diameters while on study.

12.3.3 Non-target lesions:

All other lesions (or sites of disease) including pathological lymph nodes should be identified as **non-target lesions** and should also be recorded at baseline. Measurements are not required and these lesions should be followed as 'present', 'absent', or in rare cases 'unequivocal progression'. In addition, it is possible to record multiple nontarget lesions involving the same organ as a single item on the case record form (e.g. 'multiple enlarged pelvic lymph nodes' or 'multiple liver metastases').

12.3.4 Evaluation of Non-Target Lesions

12.3.4.1 Complete Response (CR): Disappearance of all non-target lesions and normalization of tumor marker level. All lymph nodes must be non-pathological in size (<10mm short axis).

12.3.4.2 Non-CR/Non-PD:

Persistence of one or more non-target lesion(s) and/or maintenance of tumor marker level above the normal limits.

12.3.4.3 Progressive Disease (PD): Unequivocal progression of existing non-target lesions. (Note: the appearance of one or more new lesions is also considered progression).

12.3.5 Evaluation of Best Overall Response

The best overall response is the best response recorded from the start of the study treatment until the end of treatment.

Table 3: Time point response

**Target Lesions**	**Non-Target Lesions**	**New Lesions**	**Overall Response**
CR	CR	No	CR
CR	Non CR/Non PD	No	PR

CR	Not evaluated	No	PR
PR	Non PD or not all evaluated	No	PR
SD	Non PD or not all evaluated	No	SD
Not all evaluated	Non PD	No	NE
PD	Any	Yes or no	PD
Any	PD	Yes or no	PD
Any	Any	Yes	PD

CR= completeresponse, PR= partial response, PD= progressive disease, SD= stable disease, NE= not evaluable.
* In exceptional circumstances, unequivocal progression in non-target lesions may be accepted as disease progression.
Note: Patients with a global deterioration of health status requiring discontinuation of treatment without objective evidence of disease progression at that time should be reported as "*symptomatic deterioration*". Every effort should be made to document the objective progression even after discontinuation of treatment.

12.4 Duration of Response

12.4.1 Duration of overall response: The duration of overall response is measured from the time measurement criteria are met for CR or PR (whichever is first recorded) until the first date that recurrent or progressive disease is objectively documented (taking as reference for progressive disease the smallest measurements recorded since the treatment started). The duration of overall CR is measured from the time measurement criteria are first met for CR until the first date that recurrent disease is objectively documented.

12.4.2 Duration of stable disease: Stable disease is measured from the start of the treatment until the criteria for progression are met, taking as reference the smallest measurements recorded since the treatment started.

12.5 Immune-Related Response Criteria

12.5.1 Introduction

Increasing clinical experience indicates that traditional response criteria (e.g., Response Evaluation Criteria in Solid Tumors, Version 1.1 [RECIST v1.1] and World Health Organization [WHO]) may not be sufficient to characterize fully activity in the new era of target therapies and/or biologics. In studies with cytokines, cancer vaccines, and monoclonal antibodies, complete response, partial response, or stable disease has been shown to occur after an increase in tumor burden as characterized by progressive disease by traditional response criteria. Therefore, conventional response criteria may not adequately assess the activity of immunotherapeutic agents because progressive disease (by initial radiographic evaluation) does not necessarily reflect therapeutic

failure. Long-term effect on the target disease must also be captured. The immune-related response criteria (irRC) are criteria that attempt to do that by enhancing characterization of new response patterns that have been observed with immunotherapeutic agents (i.e., ipilimumab). (Note: The irRC only index and measurable new lesions are taken into account.)

12.5.2 Glossary

Term	Definition
SPD	sum of the products of the two largest perpendicular diameters
Tumor burden	$SPD_{index\ lesions} + SPD_{new,\ measurable\ lesions}$
Nadir	minimally recorded tumor burden
irCR	immune-related complete response
irPD	immune-related progressive disease
irPR	immune-related partial response
irSD	immune-related stable disease
irBOR	immune-related best overall response

12.5.3 Baseline Assessment Using irRC

Step 1. Identify the index lesions (five lesions per organ, up to ten visceral lesions and five cutaneous lesions).

Step 2. Calculate the SPD of all of these index lesions:

$$SPD = \sum_{i} \text{(Largest diameter of lesion i)} \times \text{(Second largest diameter of lesion i)}.$$

12.5.4 Post-baseline Assessments Using irRC

Step 1. Calculate the SPD of the index lesions.

Step 2. Identify new, measurable lesions (≥ 5 × 5 mm; up to five new lesions per organ: five new cutaneous lesions and ten visceral lesions).

Step 3. Calculate the SPD of the new, measurable lesions.

Step 4. Calculate the tumor burden:

$$\text{Tumor burden} = SPD_{index\ lesions} + SPD_{new,\ measurable\ lesions}$$

Step 5. Calculate the change in tumor burden relative to baseline and the change in tumor burden relative to nadir.

Step 6. Derive the overall response using the table below.

Overall Response	Criterion
irCR	Complete disappearance of all lesions (whether measurable or not, and no new lesions) confirmed by a repeat, consecutive assessment ≥ 4 weeks from the date first documented

Overall Response	Criterion
irPR	Decrease in tumor burden ≥ 50% relative to baseline confirmed by a consecutive assessment ≥ 4 weeks from the date first documented
irSD	Criteria for irCR, irPR, and irPD are not met; does not require confirmation
irPD	Increase in tumor burden ≥ 25% relative to nadir confirmed by a consecutive assessment ≥ 4 weeks from the date first documented

irCR = immune-related complete response; irPD = immune-related progressive disease; irPR = immune-related partial response; irSD = immune-related stable disease.

12.5.5 Determination of Immune-Related Best Overall Response (irBOR)

Once a patient has completed all tumor assessments, his/her irBOR may be determined:

Condition	irBOR
At least one irCR	irCR
At least one irPR and no irCR	irPR
At least one irSD and no irCR and no irPR	irSD
At least one irPD and no irCR, no irPR, and no irSD	irPD

irBOR = immune-related best overall response; irCR = immune-related complete response; irPD = immune-related progressive disease; irPR = immune-related partial response; irSD = immune-related stable disease.

12.6 Response Review and Central Tumor Measurements

At the end of treatment, all scans (baseline and through disease progression, along with crossover scans) should be sent on a CD to the address below for imaging correlatives. These do not need to be overnighted. Please include patient's study ID number the CD/disc.

Address to send scans at the end of treatment

Tarah Ballinger
Indiana University School of Medicine
535 Barnhill Dr. RT 473
Indianapolis, IN 46228
E-mail: tarahb@iu.edu

13. ASSESSMENT OF SAFETY ADVERSE EVENT REPORTING

Safety assessments will consist of monitoring and reporting AEs and SAEs, all events of death, and any study-specific issue of concern.

13.1 General

Adverse event collection and reporting is a routine part of every clinical trial. This study will use the descriptions and grading scales found in the NCI Common Terminology Criteria for Adverse Events version 5.0 (CTCAE v.5.0) that is available at http://ctep.cancer.gov/reporting//ctc.html.

Information on all adverse events, whether reported by the participant, directly observed, or detected by physical examination, laboratory test or other means, will be collected, recorded, followed and reported as described in the following sections.

Adverse events experienced by participants will be collected and reported from initiation of study medication, throughout the study, and within 30 days of the last dose of study medication. Participants who experience an ongoing adverse event related to a study procedure and/or study medication beyond 30 days will continue to be contacted by a member of the study team until the event is resolved, stabilized, or determined to be irreversible by the participating investigator.

Participants should be instructed to report any serious post-study event(s) that might reasonably be related to participation in this study. The investigator should notify the IRB and any other applicable regulatory agency of any unanticipated death or adverse event occurring after a participant has discontinued or terminated study participation that may reasonably be related to the study.

13.2 Risks Associated with atezolizumab

The PD-L1/PD-1 pathway is involved in peripheral tolerance; therefore, such therapy may increase the risk of immune-related AEs, specifically the induction or enhancement of autoimmune conditions. AEs with potentially immune-related causes, including rash, hypothyroidism, hepatitis/transaminitis, colitis, myositis, and myasthenia gravis, have been observed in Study PCD4989g.

Although most immune-related AEs observed with immunomodulatory agents have been mild and self-limiting, such events should be recognized early and treated promptly to avoid potential major complications (Di Giacomo et al. 2010).

A more detailed safety profile of atezolizumab is provided in the atezolizumab Investigator's Brochure.

13.3 Safety Parameters and Definitions

13.3.1 Adverse Event (AE)

An AE is any unfavorable and unintended sign, symptom, or disease temporally associated with the use of an investigational medicinal product (IMP) or other protocol-imposed intervention, regardless of attribution.

This includes the following:

- AEs not previously observed in the patient that emerge during the protocol-specified AE reporting period, including signs or symptoms that were not present prior to the AE reporting period
- Complications that occur as a result of protocol-mandated interventions (e.g., invasive procedures such as cardiac catheterizations)
- If applicable, AEs that occur prior to assignment of study treatment associated with medication washout, no treatment run-in, or other protocol-mandated intervention
- Pre-existing medical conditions (other than the condition being studied) judged by the investigator to have worsened in severity or frequency or changed in character during the protocol-specified AE reporting period
- Abnormal laboratory values or diagnostic test results constitute adverse events only if they induce clinical signs or symptoms or require treatment or further diagnostic tests.

13.3.2 Serious adverse event (SAE)

An AE should be classified as an SAE if any of the following criteria are met:

- It results in death (i.e., the AE actually causes or leads to death)
- It is life threatening (i.e., the AE, in the view of the investigator, places the patient at immediate risk of death. It does not include an AE that, had it occurred in a more severe form, might have caused death.)
- It requires or prolongs inpatient hospitalization
- It results in persistent or significant disability/incapacity (i.e., the AE results in substantial disruption of the patient's ability to conduct normal life functions)
- It results in a congenital anomaly/birth defect in a neonate/infant born to a mother exposed to the IMP
- It is considered a significant medical event by the investigator based on medical judgment (e.g., may jeopardize the patient or may require medical/surgical intervention to prevent one of the outcomes listed above)
- If a patient is hospitalized to undergo a medical or surgical procedure as a result of an AE, the event responsible for the procedure, not the procedure itself, should be reported as the SAE. For example, if a patient is hospitalized to undergo coronary bypass surgery, record the heart condition that necessitated the bypass as the SAE

Events **not** considered to be serious adverse events (and therefore do not require reporting) are hospitalizations or prolonged hospitalizations for:

- routine treatment or monitoring of the studied indication, not associated with any deterioration in condition, or for elective procedures
- diagnostic, elective or pre-planned surgical procedures/treatment for a pre-existing condition that did not worsen
- efficacy measurement for the study
- emergency outpatient treatment for an event not fulfilling the serious criteria outlined above and not resulting in inpatient admission
- respite care

13.4 Assessment of Adverse Events

All AEs and SAEs, whether volunteered by the patient, discovered by study personnel during questioning, or detected through physical examination, laboratory test, or other means, will be reported appropriately. Each reported AE or SAE will be described by its duration (i.e., start and end dates), regulatory seriousness criteria if applicable, suspected relationship to the study drug atezolizumab (, and actions taken.

To ensure consistency of AE and SAE causality assessments, investigators should apply the following general guideline:

Yes

There is a plausible temporal relationship between the onset of the AE and administration of atezolizumab or carboplatin, and the AE cannot be readily explained by the patient's clinical state, intercurrent illness, or concomitant therapies; and/or the AE follows a known pattern of response to atezolizumab or carboplatin; and/or the AE abates or resolves upon discontinuation of atezolizumab or carboplatin or dose reduction and, if applicable, reappears upon re-challenge.

No

Evidence exists that the AE has an etiology other than atezolizumab or carboplatin (e.g., pre-existing medical condition, underlying disease, intercurrent illness, or concomitant medication); and/or the AE has no plausible temporal relationship to atezolizumab administration (e.g., cancer diagnosed 2 days after first dose of study drug).

13.4.1 Expectedness

- Expected: Expected adverse events are those that have been previously identified as resulting from administration of the agent. For the purposes of this study, an adverse event is considered expected when it appears in the current adverse event list, the Investigator's Brochure, the package insert or is included in the informed consent document as a potential risk.
- Unexpected: An adverse event is considered unexpected when it varies in nature, intensity or frequency from information provided in the current adverse event list, the Investigator's Brochure, the package insert or when it is not included in the informed consent document as a potential risk

- For patients receiving combination therapy, causality will be assessed individually for each protocol-mandated therapy.

13.4.2 Attribution

Attribution is the relationship between an adverse event or serious adverse event and the study treatment. Attribution will be assigned as follows:

- Definite – The AE is clearly related to the study treatment.
- Probable – The AE is likely related to the study treatment.
- Possible – The AE may be related to the study treatment.
- Unlikely - The AE is doubtfully related to the study treatment.
- Unrelated - The AE is clearly NOT related to the study treatment.

Procedures for Eliciting, Recording, and Reporting Adverse Events

13.5 Eliciting Adverse Events

A consistent methodology for eliciting AEs at all subject evaluation time points should be adopted. Examples of non-directive questions include:

· "How have you felt since your last clinical visit?"
· "Have you had any new or changed health problems since you were last here?"

13.5.1 Specific Instructions for Recording Adverse Events

Investigators should use correct medical terminology/concepts when reporting AEs or SAEs. Avoid colloquialisms and abbreviations. All adverse events will be captured on the appropriate study-specific case report forms (CRFs).

13.5.2 Diagnosis versus Signs and Symptoms

If known at the time of reporting, a diagnosis should be reported rather than individual signs and symptoms (e.g., record only liver failure or hepatitis rather than jaundice, asterixis, and elevated transaminases). However, if a constellation of signs and/or symptoms cannot be medically characterized as a single diagnosis or syndrome at the time of reporting, it is acceptable to report the information that is currently available. If a diagnosis is subsequently established, it should be reported as follow-up information.

13.5.3 Deaths

All deaths that occur during the protocol-specified AE reporting period, regardless of attribution, will be reported to the appropriate parties. When recording a death, the event or condition that caused or contributed to the fatal outcome should be reported as the single medical concept. If the cause of death is unknown and cannot be ascertained at the time of reporting, report "Unexplained Death." Deaths that occur during the protocol-specified adverse event reporting period that are attributed by the investigator solely to progression of disease should be recorded only in the study CRF.

13.5.4 Pre-existing Medical Conditions

A pre-existing medical condition is one that is present at the start of the study. Such conditions should be reported as medical and surgical history. A pre-existing medical condition should be re-assessed throughout the trial and reported as an AE or SAE only if the frequency, severity, or character of the condition worsens during the study. When reporting such events, it is important to convey the concept that the pre-existing condition has changed by including applicable descriptors (e.g., “more frequent headaches”).

13.5.5 Hospitalizations for Medical or Surgical Procedures

Any AE that results in hospitalization or prolonged hospitalization should be documented and reported as an SAE. If a subject is hospitalized to undergo a medical or surgical procedure as a result of an AE, the event responsible for the procedure, not the procedure itself, should be reported as the SAE. For example, if a subject is hospitalized to undergo coronary bypass surgery, record the heart condition that necessitated the bypass as the SAE.

Hospitalizations for the following reasons do not require reporting:

- Hospitalization or prolonged hospitalization for diagnostic or elective surgical procedures for preexisting conditions
- Hospitalization or prolonged hospitalization required to allow efficacy measurement for the study or
- Hospitalization or prolonged hospitalization for scheduled therapy of the target disease of the study.

13.5.6 Assessment of Severity of Adverse Events

The adverse event severity grading scale for the NCI CTCAE (v5.0 Update current versions) will be used for assessing adverse event severity. Below Table should be used for assessing severity for adverse events that are not specifically listed in the NCI CTCAE.

Adverse Event Severity Grading Scale for Events Not Specifically Listed in NCI CTCAE

Grade	Severity
1	Mild; asymptomatic or mild symptoms; clinical or diagnostic observations only; or intervention not indicated
2	Moderate; minimal, local, or non-invasive intervention indicated; or limiting age-appropriate instrumental activities of daily living a

3	Severe or medically significant, but not immediately life-threatening; hospitalization or prolongation of hospitalization indicated; disabling; or limiting self-care activities of daily living b,c
4	Life-threatening consequences or urgent intervention indicated d
5	Death related to adverse event d

NCI CTCAE = National Cancer Institute Common Terminology Criteria for Adverse Events.

Note: Based on the most recent version of NCI CTCAE v.5.0 which can be found at: http://ctep.cancer.gov/protocolDevelopment/electronic_applications/ctc.htm

a. Instrumental activities of daily living refer to preparing meals, shopping for groceries or clothes, using the telephone, managing money, etc.

b. Examples of self-care activities of daily living include bathing, dressing and undressing, feeding oneself, using the toilet, and taking medications, as performed by patients who are not bedridden.

c. If an event is assessed as a "significant medical event," it must be reported as a serious adverse event

d. Grade 4 and 5 events must be reported as serious adverse events

Case Transmission Verification of Single Case Reports

The Sponsor agrees to conduct the Case Transmission verification to ensure that all single case reports have been adequately received by Genentech via the Investigator emailing Genentech a Quarterly line-listing documenting single case reports sent by the Investigator to Genentech in the preceding time period.
The periodic line-listing will be exchanged within seven (7) calendar days of the end of the agreed time period. Confirmation of receipt should be received within the time period mutually agreed upon.

If discrepancies are identified, the Sponsor and Genentech will cooperate in resolving the discrepancies. The responsible individuals for each party shall handle the matter on a case-by-case

basis until satisfactory resolution. The sponsor shall receive reconciliation guidance documents within the ‘Activation Package’.

Following Case Transmission Verification, single case reports which have not been received by Genentech shall be forwarded by the Investigator to Genentech within five (5) calendar days from request by Genentech.

At the end of the study, a final cumulative Case Transmission Verification report will be sent to Genentech

13.5.7 Pregnancies in Female Patients

Female patients of childbearing potential will be instructed to immediately inform the investigator if they become pregnant during the study or within 90 days after the last dose of study drug. A Pregnancy Report should be submitted to the VICC Coordinating Center (i.e., no more than 24 hours after learning of the pregnancy). Pregnancy should not be recorded as an adverse event. The investigator should discontinue study drug and counsel the patient, discussing the risks of the pregnancy and the possible effects on the fetus. Monitoring of the patient should continue until conclusion of the pregnancy. Any SAEs associated with the pregnancy (e.g., an event in the fetus, an event in the mother during or after the pregnancy, or a congenital anomaly/birth defect in the child) should be reported appropriately (see SAE reporting section 13.5.11.2).

13.5.8 Pregnancies in Female Partners of Male Patients

Male patients will be instructed through the ICF to immediately inform the investigator if their partner becomes pregnant during the study or within 90 days after completing treatment with atezolizumab. Male patients who received study treatment should not attempt to father a child until after this 90 day period. A Pregnancy Report should be completed by the investigator immediately (i.e., no more than 24 hours after learning of the pregnancy) and submitted to the VICC Coordinating Center for processing. Attempts should be made to collect and report details of the course and outcome of any pregnancy in the partner of a male patient exposed to study drug. The pregnant partner will need to sign an Authorization for Use and Disclosure of Pregnancy Health Information to allow for follow-up on her pregnancy. Once the authorization has been signed, the investigator will update the Pregnancy Report with additional information on the course and outcome of the pregnancy. An investigator who is contacted by the male patient or his pregnant partner may provide information on the risks of the pregnancy and the possible effects on the fetus, to support an informed decision in cooperation with the treating physician and/or obstetrician.

13.5.9 Abortions

Any spontaneous abortion should be classified as an SAE (as the Sponsors consider spontaneous abortions to be medically significant events), recorded as such , and reported to the Vanderbilt Coordinating Center (i.e., no more than 24 hours after learning of the event).

13.5.10Congenital Anomalies/Birth Defects

Any congenital anomaly/birth defect in a child born to a female patient or female partner of a male patient exposed to study drug should be classified as an SAE, recorded as such, and reported to the Vanderbilt Coordinating Center (i.e., no more than 24 hours after learning of the event).

13.5.11 Adverse Events of Special Interest

AESIs are a subset of Events to Monitor (EtMs) of scientific and medical concern specific to the product, for which ongoing monitoring and rapid communication by the Investigator to the Sponsor is required. Such an event might require further investigation in order to characterize and understand it. Depending on the nature of the event, rapid communication by the trial Sponsor to other parties (e.g., Regulatory Authorities) may also be warranted.

Adverse events of special interest (AESIs) are defined as a potential safety problem, identified as a result of safety monitoring of the IMP.

The following AEs are considered of special interest for Atezolizumab and must be reported to the Coordinating Center expeditiously (following the SAE reporting procedure), irrespective of regulatory seriousness criteria:

- Pneumonitis
- Colitis
- Endocrinopathies: diabetes mellitus, pancreatitis, hyperthyroidism or hypophysitis
- Hepatitis, including AST or ALT >10x ULN
- Systemic lupus erythematosus
- Neurological disorders: Guillain-Barré syndrome, myasthenic syndrome or myasthenia gravis, and meningoencephalitis
- Events suggestive of hypersensitivity, cytokine release syndrome, influenza-like illness, systemic inflammatory response syndrome, or infusion-reaction syndromes
- Nephritis
- Ocular toxicities (e.g. uveitis, retinitis, optic neuritis)
- Myositis
- Myopathies, including rhabdomyolysis
- Grade ≥ 2 cardiac disorders (e.g., atrial fibrillation, myocarditis, pericarditis)
- Vasculitis
- Autoimmune hemolytic anemia

Severe cutaneous reactions (e.g., Stevens-Johnson syndrome, dermatitis bullous, toxic epidermal necrolysis)

Non Drug Specific AESIs which require immediately reporting:

- Cases of potential drug-induced liver injury that include an elevated ALT or AST in combination with either an elevated bilirubin or clinical jaundice, as defined by Hy's law:
- Treatment-emergent ALT or AST > 3 x ULN in combination with total bilirubin > 2 x ULN
- Treatment-emergent ALT or AST > 3 x ULN in combination with clinical jaundice
- Data related to a suspected transmission of an infectious agent by the study drug (STIAMP), as defined below:
    - Any organism, virus, or infectious particle (e.g., prion protein transmitting transmissible spongiform encephalopathy), pathogenic or non-pathogenic, is considered an infectious agent. A transmission of an infectious agent may be suspected from clinical symptoms or laboratory findings that indicate an infection in a patient exposed to a medicinal product. This term applies only when a contamination of the study drug is suspected.

13.5.12 Exchange of Single Case Reports

The Sponsor will be responsible for collecting all protocol-defined Adverse Events (AEs)/Serious Adverse Events (SAEs), AEs of Special Interest (AESIs), Special Situation Reports (including pregnancy reports) and Product Complaints (with or without an AE) originating from the Study for the Product.

Investigators must report all the above mentioned single case reports adequately to Genentech within the timelines described below. The completed MedWatch or CIOMS I form or Genentech approved reporting forms should be faxed/emailed immediately upon completion to Genentech at the following contacts:

All protocol-defined AEs, SAEs, AESIs, Special Situation Reports (including pregnancy reports) and Product Complaints **with** an AE should be sent to:

Fax: 650-238-6067
Email: usds_aereporting-d@gene.com

All Product Complaints **without** an AE should be sent to:

Email: kaiseraugst.global_impcomplaint_management@roche.com

It is understood and agreed that the Sponsor will be responsible for the evaluation of AEs/SAEs, AESIs, Special Situation Reports (including pregnancy reports) and Product Complaints (with or without an AE) originating from the study.

These single case reports will be exchanged between the parties as outlined below so that regulatory obligations are met.

13.5.13 Serious Adverse Events

All serious adverse events, regardless of causality to study drug, occurring after the patient's first dose of study treatment will be reported to the Principal Investigator and/or the Study Coordinator at each institution, and also to the Coordinating Center. Serious adverse events that occur during screening do not require reporting unless they are due to a screening procedure (eg biopsy).

Serious adverse events (SAEs), AEs of Special Interest (AESIs), pregnancy reports (including pregnancy occurring in the partner of a male study subject), other Special Situation Reports and Product Complaints (with or without an AE), where the patient has been exposed to the Genentech Product, will be sent on a MedWatch form or CIOMS I form or on Genentech approved reporting forms to Genentech Drug Safety. Transmission of these reports (initial and follow-up) will be either electronically or by fax and within the timelines specified below:

· SADRs

Serious AE reports that are related to the Product shall be transmitted to Genentech within fifteen (15) calendar days of the awareness date.

· Other SAEs

Serious AE reports that are unrelated to the Product shall be transmitted to Genentech within thirty (30) calendar days of the awareness date.

· AESIs

AESIs shall be forwarded to Genentech within fifteen (15) calendar days of the awareness date.

· Special Situation Reports

Pregnancy reports

While such reports are not serious AEs or Adverse Drug Reactions (ADRs) per se, as defined herein, any reports of pregnancy (including pregnancy occurring in the partner of a male study subject), where the fetus may have been exposed to the Product, shall be transmitted to Genentech within thirty (30) calendar days of the awareness date. Pregnancies will be followed up until the outcome of the pregnancy is known, whenever possible, based upon due diligence taken to obtain the follow-up information.

· Other Special Situation Reports

In addition to all SAEs, pregnancy reports and AESIs, the following other Special Situations Reports should be collected even in the absence of an Adverse Event and transmitted to Genentech within thirty (30) calendar days:

· Data related to the Product usage during breastfeeding

· Data related to overdose, abuse, misuse or medication error (including potentially exposed or intercepted medication errors)

In addition, reasonable attempts should be made to obtain and submit the age or age group of the patient, in order to be able to identify potential safety signals specific to a particular population

13.5.13.1 Post-Study Adverse Events

The investigator should expeditiously report any SAE occurring after a patient has completed or discontinued study participation if attributed to prior atezolizumab exposure. If the investigator should become aware of the development of cancer or a congenital anomaly in a subsequently conceived offspring of a female patient who participated in the study, this should be reported as an SAE.

13.5.11.2 SAE Reporting

All serious adverse events must be reported to the Coordinating Center within 1 business day after the treating institution becomes aware of the event. Events should be reported using the Vanderbilt Serious Adverse Event Form, which is part of the packet of supplemental forms.

The form must be fully completed and emailed to:

ATTN: VICC CTSR Personnel
EMAIL: Coordinating.Center@vumc.org
PHONE: (615) 936-5770

The email subject line should include the study number, patient number, and clearly state SAE initial or follow up report. Follow-up information must also be reported within 1 business day of receipt of the information by the investigator.

The Coordinating Center will disseminate information regarding unexpected serious adverse events to the participating sites quarterly after review of the information by the Protocol Chair (or her designee in the event of extended absence) only in the case that the event(s) is believed to be related (i.e., possibly, probably, or definitely) to the study medication. The Coordinating Center will be responsible for reporting of events to the FDA and supporters, as appropriate (outlined below).

13.5.14 Product Complaints

A product complaint is any written or oral information received from a complainant that alleges deficiencies related to identity, quality, safety, strength, purity, reliability, durability, effectiveness or performance of a product after it has been released and distributed to the commercial market or clinical trial. All product complaints (with or without an AE) must be reported as an SAE to the

Coordinating Center within 1 business day after the treating institution becomes aware of the event, according to SAE reporting instructions in protocol Section 13.5.11.2.

13.5.15 Institutional Review Board

All adverse events and serious adverse events will be reported to the IRB per current institutional standards. If an adverse event requires modification of the informed consent or the protocol, these modifications will be provided to the IRB, as will the report of the adverse event.

13.6 Coordinating Center Reporting Procedures

Reporting to Regulatory Authorities, Ethics Committees and Investigators

Genentech as the Marketing Authorization Holder will be responsible for the reporting of individual case safety reports from the study to the regulatory authority in compliance with applicable regulations.

The Investigator as the Sponsor of the Study, will be responsible for the expedited reporting of safety reports originating from the study to the EMA through Eudravigilance Clinical Trial Module (EVCTM), where applicable.

The Investigator will be responsible for the expedited reporting of safety reports originating from the Study to the Ethics Committees and Institutional Review Boards (IRB), where applicable. The Investigator will be responsible for the distribution of safety information to its own investigators, where relevant, in accordance with local regulations.

13.6.1 Food and Drug Administration (FDA)

Unexpected serious adverse events believed to be definitely, probably, or possibly related to the medications will be reported to the Food and Drug Administration via MedWatch Form 3500A (http://www.fda.gov/safety/medwatch/howtoreport/downloadforms/default.htm) by telephone 1-800-FDA-1088; or by fax 1-800-FDA-0178.The Coordinating Center will be responsible for correspondence regarding adverse events with the FDA for all participating sites.

13.6.2 Additional Reporting Requirements for IND

IND Safety Reports

Events meeting the following criteria need to be submitted to the FDA as expedited IND Safety Reports according to the following guidance and timelines:

7 Calendar Day Telephone or Fax Report

The investigator is required to notify the FDA of any fatal or life-threatening AE that is unexpected and assessed by the investigator to be possibly related to the use of atezolizumab. An unexpected AE is one that is not already described in the atezolizumab Investigator's Brochure. Such reports are to be telephoned or faxed to the FDA and Genentech within 7 calendar days of first learning of the event.

15 Calendar Day Written Report
The Investigator is also required to notify the FDA and all participating investigators, in a written IND Safety Report, of any serious, unexpected AE that is considered reasonably or possibly related to the use of atezolizumab. An unexpected AE is one that is not already described in the atezolizumab Investigator's Brochure.

Written IND Safety reports should include an Analysis of Similar Events in accordance with regulation 21 CFR § 312.32. All safety reports previously filed by the investigator with the IND concerning similar events should be analyzed and the significance of the new report in light of the previous, similar reports commented on.

Written IND safety reports with analysis of similar events are to be submitted to the FDA, Genentech, and all participating investigators within 15 calendar days of first learning of the event. The FDA prefers these reports on a MedWatch 3500 form, but alternative formats are acceptable (e.g., summary letter).

Contact Information for IND Safety Reports
FDA fax number for IND safety reports:

Fax: (800) FDA-0178

All written IND safety reports submitted to the FDA by the investigator must also be faxed to the local site IRB and Genentech at:

Fax: (650) 225-4682 or (650) 225-4630

Genentech Drug Safety CTV mailbox: **ctvist_drugsafety@gene.com**

And the Investigator will be responsible for the distribution of safety information to Site IRB.

For questions related to safety reporting, please contact Genentech Drug Safety:

Tel: (888) 835-2555

Fax: (650) 225-4682 or (650) 225-4630

IND Annual Reports

All IND annual reports submitted to the FDA by the Sponsor-Investigator should be copied to Genentech

Copies of such reports should be emailed to Genentech at: Genentech Drug Safety CTV mail box:

ctvist_drugsafety@gene.com

Other Reports
The Sponsor will forward a copy of the Final Study Report to Genentech upon completion of the Study.

All FDA Reports
Any study report submitted to the FDA by the Sponsor-investigator should be copied to Genentech. This includes all IND annual reports and the Clinical Study Report (final study report). Additionally, any literature articles that are a result of the study should be sent to Genentech. Copies of such reports should be mailed to the assigned Clinical Operations contact for the study.

13.6.3 Genentech

The Sponsor-investigator must report all SAEs to Genentech within the timelines described below. The completed MedWatch or comparable SAE form should be faxed or emailed immediately upon completion to Genentech Drug Safety at:

(650) 225-4682 or (650) 225-4360

usds_aereporting-d@gene.com

Relevant follow-up information should be submitted to Genentech Drug Safety as soon as it becomes available

Additional reporting requirements to Genentech include the following:

- Any reports of pregnancy following the start of administration with the atezolizumab and within the follow-up period (for female patients within one year after the last dose of atezolizumab or the partner of a male patient within three months of completing therapy) This includes reports of abortions and congenital anomalies /birth defects. Pregnancies will be followed up until the outcome of the pregnancy is known, whenever possible, based upon due diligence taken to obtain the follow-up information.
- All non-serious atezolizumab AEs originating from the study will be forwarded Genentech quarterly.

Note: Investigators should also report events to their IRB as required.

In addition to SAEs, pregnancy reports and AESIs, the following safety reports should be collected and transmitted to Genentech/Roche even in the absence of an Adverse Event within thirty (30) calendar days:

- Data related to product usage during pregnancy or breastfeeding

- Data related to overdose, abuse, misuse, inadvertent/erroneous administration, medication error or occupational exposure, with or without association with an AE/SAE unless otherwise specified in the protocol
- Data related to a suspected transmission of an infectious agent via a medicinal product (STIAMP)
- Lack of therapeutic efficacy

In addition, reasonable attempts should made to obtain and submit the age or age group of the patient, in order to be able to identify potential safety signals specific to a particular population

.

Safety Reconciliation

The Sponsor-investigator agrees to conduct reconciliation for the product. Genentech and the Sponsor-investigator will agree to the reconciliation periodicity and format, but agree at minimum to exchange quarterly line listings of cases received by the other party. If discrepancies are identified, the Sponsor-investigator and Genentech will cooperate in resolving the discrepancies. The responsible individuals for each party shall handle the matter on a case-by-case basis until satisfactory resolution. The sponsor shall receive reconciliation guidance documents within the 'Activation Package.'

MedWatch 3500A Reporting Guidelines

In addition to completing appropriate patient demographic and suspect medication information, the report should include the following information within the Event Description (item 5) of the MedWatch 3500A form:

- Protocol description (and number, if assigned)
- Description of event, severity, treatment, and outcome if known
- Supportive laboratory results and diagnostics
- Investigator's assessment of the relationship of the AE to each investigational product and suspect medication

Follow-Up Information

Additional information may be added to a previously submitted report by any of the following methods:

- Adding to the original MedWatch 3500A report and submitting it as follow-up
- Adding supplemental summary information and submitting it as follow-up with the original MedWatch 3500A form
- Summarizing new information and faxing it with a cover letter including patient identifiers (i.e., date of birth, initial, patient number), protocol description and number, if assigned, brief AE description, and notation that additional or follow-up information is being submitted. (The patient identifiers are important so that the new information is added to the correct initial report.)

Occasionally Genentech may contact the reporter for additional information, clarification, or current status of the patient for whom and AE was reported. For questions regarding SAE reporting, you may contact a Genentech Drug Safety representative. Relevant follow-up information should be submitted to Genentech Drug Safety as soon as it becomes available and/or upon request.

MedWatch 3500A (Mandatory Reporting) form is available at https://www.fda.gov/media/69876/download

STUDY CLOSE-OUT

Any study report submitted to the FDA by the Sponsor-Investigator should be copied to Genentech/Roche. This includes all IND annual reports and the Clinical Study Report (final study report). Additionally, any literature articles that are a result of the study should be sent to Genentech/Roche. Copies of such reports should be mailed to the assigned Clinical Operations contact for the study:

anti-pdl-1-mpd3280a-gsur@gene.com

And to Genentech Drug Safety CTV oversight mail box at: **ctvist_drugsafety@gene.com**

QUERIES

Queries related to the Study will be answered by the Investigator. However, responses to all safety queries from regulatory authorities or for publications will be discussed and coordinated between the Parties. The Parties agree that Genentech/Roche shall have the final say and control over safety queries relating to the Product. The Investigator agrees that it shall not answer such queries from regulatory authorities and other sources relating to the Product independently but shall redirect such queries to Genentech/Roche.

Both Parties will use all reasonable effort to ensure that deadlines for responses to urgent requests for information or review of data are met. The Parties will clearly indicate on the request the reason for urgency and the date by which a response is required.

SAFETY CRISIS MANAGEMENT

In case of a safety crisis, e.g., where safety issues have a potential impact on the indication(s), on the conduct of the Study, may lead to labeling changes or regulatory actions that limit or restrict the way in which the Product is used, or where there is media involvement, the Party where the crisis originates will contact the other Party as soon as possible.

The Parties agree that Genentech/Roche shall have the final say and control over safety crisis management issues relating to the Product. The Investigator agrees that it shall not answer such queries from media and other sources relating to the Product but shall redirect such queries to Genentech/Roche.

14. DATA SAFETY AND MONITORING

14.1 Data Management and Reporting

Participating institutions will be collaborating with Vanderbilt in patient accrual. Data will be collected using a centralized electronic case report form called ON-line Clinical Oncology Research Environment = Oncore (http://www.vicc.org/ct/research/oncore.php). Oncore is a highly secure, web based, cancer specific, and customizable system that provides fully integrative clinical data management and study administration capabilities developed in an ongoing collaborative effort with NCI designated Comprehensive Cancer Centers. It fully integrates study administration functionality including protocol tracking, patient registration, NCI reporting, review committee tracking, and SAE tracking, with clinical data management functionality including electronic case report forms (eCRF) design, clinical data capture, protocol and regulatory compliance monitoring. Also the system is capable in storing basic protocol information (e.g., IRB approval dates, dates for annual renewals,) and clinical trials research data. Oncore allows the investigator to define specific protocol requirements and generate data collection forms. Creation of the data collection form is done with a single button click after the parameters of an individual protocol have been specified. Oncore permits specification of study protocols, management of patient enrollment, clinical data entry and viewing, and the generation of patient or study-specific reports based on time stamping. OnCore is embedded with a comprehensive domain repository of standard reference codes and forms to promote standardization. The sources for the repository include CDUS, CTC, CDEs from NCI, ICD, MedDRA and various best practices from contributing NCI-designated Comprehensive Cancer Centers. OnCore provides several reporting features specifically addressing NCI Summary 3 and Summary 4 and other reporting requirements. Data may also be exported in a format suitable for import into other database, spreadsheets or analysis systems (such as SPSS). This system will be used to manage all VICCC clinical trials data. OnCore is maintained and supported in the VICC Clinical and Research Informatics Resource.

Specified members at each participating site will submit all pertinent regulatory documents to the Coordinating Center, who will store it in a secure location.

The Principal Investigator or designee will inform Genentech as defined in the Safety and Data Exchange Agreement (SDEA) of any serious adverse event, and will inform the IRB in accordance with each institution's IRB policy. The investigator is responsible for the detection and documentation of events meeting the criteria and definition of an AE or SAE, as provided in this protocol. During the study when there is a safety evaluation, the treating investigator or site staff will be responsible for detecting, documenting, and report AEs and SAEs, as detailed in the protocol. If any problem is identified related to the conduct of this research, the VICC Data Safety and Monitoring Committee (DSMC) will be formally asked to review the study and the situation that required DSMC intervention.

14.2 Meetings

This trial will be monitored by the VICC Breast Cancer Research Team. The Breast Cancer Research Team is composed of the Clinical Core Director of the Breast Cancer Program and Team Leader, Surgical Oncologists, Radiation Oncologists, Medical Oncologists, Research Nurses, the Data Manager, and our Regulatory Specialist. The Breast Cancer Research Team meets on a

monthly basis to discuss all AEs/SAEs, accrual, compliance, safety issues, adherence to protocol, reviews, etc. pertaining to all breast cancer studies. This particular study will be thoroughly reviewed during these meetings. These monthly meetings have minutes recorded each time and those are also reviewed on a monthly basis by the Breast Cancer Research Team Physician Leader.

14.3 Monitoring

This trial will be monitored continuously by the study's Protocol Chair and by the Breast Cancer Research Team at VUMC. The Vanderbilt-Ingram Cancer Center (VICC) oversees patient safety and data monitoring for its investigator-initiated and NIH-NCI funded clinical trials through its Data and Safety Monitoring Committee (DSMC). The purpose of the DSMC is to ensure the efficient implementation and management of the VICC Data and Safety Monitoring Plan (DSMP). The Committee maintains authority to intervene in the conduct of studies as necessary to ensure clinical research performed at VICC achieves the highest quality standards.

The VICC DSMC meets on a quarterly basis and ad hoc to discuss data and safety monitoring of clinical trials and to oversee the VICC DSMP. Internal audits for compliance with adverse event reporting, regulatory and study requirements, and data accuracy and completion are conducted according to the VICC DSMP according to study phase and risk. The committee reviews all serious adverse events (SAE) on Vanderbilt sponsored investigator-initiated studies on a quarterly basis and provides DSMC SAE review reports to the Vanderbilt IRB.

Additionally, regular interim monitoring will be conducted by the Coordinating Center remotely.

14.4 Data Handling and Record Keeping

14.4.1 Case Report Forms

An electronic case report form (eCRF) is required and must be completed for each included participant. The completed dataset should not be made available in any form to third parties, except for authorized representatives of appropriate Health/Regulatory Authorities, without written permission from Vanderbilt.

14.4.2 Record Retention

To enable evaluations and/or audits from Health Authorities and Vanderbilt, each site investigator agrees to keep records including: The identity of all participants (sufficient information to link records; e.g., hospital records), all original signed informed consent forms, copies of all source documents, and detailed records of drug disposition. To comply with international regulations, the records should be retained by the investigator in compliance with regulations.

During data entry, range and missing data checks will be performed on-line. The checks to be performed will be documented in the Data Monitoring Plan for the study. A summary report (QC Report) of these checks together with any queries resulting from manual review of the eCRF's will be generated for each site and transmitted to the site and the site monitor. Corrections will be made by the study site personnel. This will be done on an ongoing basis.

15. REGULATORY CONSIDERATIONS

15.1 Protocol Review and Amendments

Information regarding study conduct and progress will be reported to the Institutional Review Board (IRB) per the current institutional standards of each participating center.

Any changes to the protocol will be made in the form of an amendment and must be approved by the IRB of each institution prior to local implementation.

The Protocol Chair (or her designee) is responsible for the coordination and development of all protocol amendments, and will disseminate this information to the participating centers.

15.2 Informed Consent

The investigator (or his/her designee) will explain to each subject the nature of the study, its purpose, the procedures involved, the expected duration, the potential risks and benefits involved and any discomfort it may entail. Each subject will be informed that participation in the study is voluntary, that s/he may withdraw from the study at any time, and that withdrawal of consent will not affect her subsequent medical treatment or relationship with the treating physician(s) or institution. The informed consent will be given by means of a standard written statement, written in non-technical language, which will be IRB approved. The subject should read and consider the statement before signing and dating it, and will be given a copy of the signed document. No subject will enter the study or have study-specific procedures done before his/her informed consent has been obtained.

Confidentiality and security will be maintained for the tissue collection within this study. All tissue samples obtained for this study will be assigned a code and this code used to identify the sample. The samples will not be labeled with the patient's name, address or other information that would identify them. All information will be coded to maintain privacy. Research data, including the data collected from the medical charts will be entered into a password-protected database. The database (Breast Cancer Program Database) in which this study data is going to be stored has a firewall (in addition to the institutional firewall) with the highest level of protection, i.e. the same level of protection as the on-line hospital information system at Vanderbilt. This means that users must log on to a web server that sits between the institutional firewall and the firewall to the database, and only this application server is allowed to query the database. Information, including the identifier and password for the authorized users, is transmitted via a secure shell protocol using 128k encryption. Only Dr. Vandana Abramson, the PI, and the Breast Team Data Manager, approved through our institutional review board will be allowed access to patient identifiers. Other levels of authorization may exist for approved users, e.g. access to de-identified data. This database will store a de-identified link to the patient data and will not otherwise store patient data, even de-identified. The safety monitoring will be performed by the groups deemed appropriate by the Vanderbilt University Medical Center Institutional Review Board for reviewing the clinical trials procedure. Safety monitoring for the database is also performed by the Networking and Security Services of the Vanderbilt University Medical Center. Audit trails for access to the web server and the databases behind the dual firewall system are maintained in accordance with the practices of the Networking and Security Services of the Vanderbilt University Medical Center.

In accordance with the Health Information Portability and Accountability Act (HIPAA), the written informed consent document (or a separate document to be given in conjunction with the consent document) will include a subject authorization to release medical information to the study sponsor and supporting agencies and/or allow these bodies, a regulatory authority, or Institutional Review Board access to subjects' medical information that includes all hospital records relevant to the study, including subjects' medical history.

15.3 Ethics and GCP

This study will be carried out in compliance with the protocol and Good Clinical Practice, as described in:

1. ICH Harmonized Tripartite Guidelines for Good Clinical Practice 1996.
2. US 21 Code of Federal Regulations dealing with clinical studies (including parts 50 and 56 concerning informed consent and IRB regulations).
3. Declaration of Helsinki, concerning medical research in humans (Recommendations Guiding Physicians in Biomedical Research Involving Human Subjects, Helsinki 1964, amended Tokyo 1975, Venice 1983, Hong Kong 1989, Somerset West 1996).

The investigator agrees to adhere to the instructions and procedures described in it and thereby to adhere to the principles of Good Clinical Practice that it conforms to.

16. MULTI-CENTER GUIDELINES

16.1 Protocol Review and Amendments

Information regarding study conduct and progress will be reported to the Institutional Review Board (IRB) per the current institutional standards of each participating center.

Any changes to the protocol will be made in the form of an amendment and must be approved by the IRB of each institution prior to local implementation.

The Protocol Chair (or her designee) is responsible for the coordination and development of all protocol amendments. Once approved by the Protocol Chair, Vanderbilt will disseminate this information to the participating centers.

16.2 Study Documentation

Each participating site is responsible for submitting copies of all relevant regulatory documentation to the Coordinating Center. The required documents include, but are not limited to the following: local IRB approvals (i.e., protocol, consent form, amendments, patient brochures and recruitment material, etc.), IRB membership rosters, summary of unanticipated problems or protocol deviations, and documentation of expertise of the investigators. The Coordinating Center will provide each participating site with a comprehensive list of the necessary documents. It is the responsibility of the participating sites to maintain copies of all documentation submitted to the Coordinating Center.

The requirements for data management, submissions, and monitoring are outlined below. The participating sites will submit all the research related information (source documents and research records – IRB approval documents, patient registration list, CRF info, toxicity assessments, tumor measurements/ responses, etc.) to the Coordinating Center.Personnel from the VICC Clinical Trial Shared Resource will monitor the trial remotely and may periodically visit the investigative site to assure proper conduct of the trial and proper collection of the data. The investigators at other sites will allow the monitor to review all source documents used in the preparation of the case reports, whether it be in person or remotely.

16.3 Records Retention

U.S. FDA regulations (21 CFR §312.62[c]) require that records and documents pertaining to the conduct of this study and the distribution of investigational drug, including CRFs, consent forms, laboratory test results, and medication inventory records, must be retained by each Principal Investigator for 2 years after marketing application approval. If no application is filed, these records must be kept 2 years after the study is discontinued and the U.S. FDA and the applicable national and local health authorities are notified.

Following closure of the study, each participating center will maintain a copy of all site study records in a safe and secure location. The Coordinating Center will inform the investigator at each site at such time that the records may be destroyed.

16.4 Publication

It is understood that any manuscript or releases resulting from the collaborative research must be approved by the Protocol Chair and will be circulated to both Genentech and applicable participating sites/investigators prior to submission for publication or presentation.

Additionally, any publication of study data and results must conform to the publications policy as stated the Translational Breast Cancer Research Consortium's (TBCRC) "Policies and Procedures".

17. STATISTICAL CONSIDERATIONS

17.1 Study Design/ Endpoints

The primary endpoint of this trial is the median PFS of carboplatin + atezolizumab versus carboplatin alone in patients with metastatic triple negative (ER/PR/HER2 negative) breast cancer. Prior studies have shown a median progression free survival of 2.9 months in patients with triple negative breast cancer receiving cisplatin or carboplatin who have received up to one prior chemotherapy regimen and a stable disease rate of approximately 5% at 6 months. We therefore propose that an improvement in median PFS of 2.9 months for single agent carboplatin to 4.4 months for the combination of carboplatin with atezolizumab would render this therapy worthy of further study. The overall PFS data will be estimated using the Kaplan-Meier method with 95% confidence intervals.

17.2 Sample Size/ Accrual Rate

The sample size estimation was completed using the log-rank test. A total sample size of 106 (n=53 in each arm) provides 80% power to detect a 1.5 month improvement in the median PFS, i.e. 2.9 months vs. 4.4 months, with a one-sided significance level equal to .10 (type I error). This estimation is based on the assumptions that accrual time is 24 months, additional follow-up time is 12 months, , and the ratio randomization is 1:1.

17.3 Analysis of Secondary and Exploratory Endpoints

We will calculate the ORR, CBR, DOR, and OS and corresponding 95% confidence intervals. PFS, ORR, and DOR will also be analyzed using irRECIST.

RNA-seq will be performed on all biopsies to assign a triple negative subtype, to determine baseline and treatment-induced changes in GE after therapy and define mutations present in the tumors both at baseline and upon progression of the disease. We expect an enrichment of drug resistance-associated mutations/gene amplifications particularly in tumors that regress and later recur while on therapy. Somatic SNPs and structural variants will be validated by whole exome sequencing (WES) of genomic DNA from the tumor and matched blood. We will validate mutations by targeted capture approaches that allow high-level coverage (greater than 200X) and analysis of clonality. The status of p53, BRCA1/2, PIK3CA, PTEN, INPP4B and other mutations associated with TNBC, along with PDL1 expression and TILs in the tumor tissue is critical for testing our hypothesis. We anticipate significant opportunity for discovery of novel mutations as well as those arising from selection during resistance to the therapies under investigation. Statistics on the correlative endpoints (tissue assays) will be primarily descriptive. Therefore, these studies are considered to be exploratory and potentially hypothesis-generating.

Regarding the impact of body composition on immune phenotype and prognosis in patients receiving PDL1 blockade for metastatic triple negative breast cancer in this study, data analysis will be primarily exploratory. Pearson's rho will be used to estimate the correlation between low muscle mass (sarcopenia) and T cell exhaustion (PD1 and CTLA4 expression). Additional exploratory analysis will include the linear regression model to adjust for the potential confounders such as age and gender. It is anticipated this sudy would provide data from 106 patients. Assuming about 10% may not be able to provide evaluable data, we will have the complete data from 95 patients. A sample size of 95 would have 84.7% power to detect a true correlation rho=0.3 versus the null hypothesis rho=0, with a type-I error of 0.05. To evaluate the impact of body composition on prognosis by progression free survival in patients receiving immunotherapy, we will evaluate only the study cohort receiving atezolizumab (n = 53). A Cox regression will be used to investigate the association between low muscle mass and PFS, while including T cell exhaustion (PD1 and CTLA4 expression) as the additional covariates as well as other potential confounders.

17.4 Statistical Analysis Plan

Demographic information, such as age and race, will be tabulated. Descriptive statistics, including means, standard deviations, and ranges for continuous parameters, as well as percents and frequencies for categorical parameters, will be presented. Investigation for outliers and assumptions for statistical analysis, e.g., normality and homoscedasticity, will be made. If necessary, data will be

transformed by utilizing appropriate transformations such as log or square root. Adverse medical events will be tabulated. NCI toxicity Grade 3 and Grade 4, and laboratory abnormalities will be listed.

17.5 Reporting and Exclusions

All patients included in the study must be assessed for safety, tolerability, and response to treatment, even if there are major protocol treatment deviations or if they are ineligible. Each patient will be assigned one of the following categories: 1) complete response, 2) partial response, 3) stable disease, 4) progressive disease, 5) early death from malignant disease, 6) early death from toxicity, 7) early death because of other cause, or 9) unknown (not assessable, insufficient data).

All of the patients who met the eligibility criteria and received study drugs for 4 weeks should be included in the main analysis of the clinical benefit rate. Patients in response categories 4-9 should be considered to have a treatment failure (disease progression). Thus, an incorrect treatment schedule or drug administration does not result in exclusion from the analysis of the response and clinical benefit rate.

REFERENCES

1. Lehmann, B.D., et al., *Identification of human triple-negative breast cancer subtypes and preclinical models for selection of targeted therapies.* J Clin Invest, 2011. **121**(7): p. 2750-67.
2. Masuda, H., et al., *Differential response to neoadjuvant chemotherapy among 7 triple-negative breast cancer molecular subtypes.* Clin Cancer Res, 2013. **19**(19): p. 5533-40.
3. Jezequel, P., et al., *Gene-expression molecular subtyping of triple-negative breast cancer tumours: importance of immune response.* Breast Cancer Res, 2015. **17**(1): p. 43.
4. Burstein, M.D., et al., *Comprehensive genomic analysis identifies novel subtypes and targets of triple-negative breast cancer.* Clin Cancer Res, 2015. **21**(7): p. 1688-98.
5. Adams, S., et al., *Prognostic value of tumor-infiltrating lymphocytes in triple-negative breast cancers from two phase III randomized adjuvant breast cancer trials: ECOG 2197 and ECOG 1199.* J Clin Oncol, 2014. **32**(27): p. 2959-66.
6. Denkert, C., et al., *Tumor-infiltrating lymphocytes and response to neoadjuvant chemotherapy with or without carboplatin in human epidermal growth factor receptor 2-positive and triple-negative primary breast cancers.* J Clin Oncol, 2015. **33**(9): p. 983-91.
7. *Pembrolizumab shows potential in breast cancer.* Cancer Discov, 2015. **5**(2): p. 100-1.
8. Chen, X., et al., *TNBCtype: A Subtyping Tool for Triple-Negative Breast Cancer.* Cancer Inform, 2012. **11**: p. 147-56.
9. Rizvi, N.A., et al., *Cancer immunology. Mutational landscape determines sensitivity to PD-1 blockade in non-small cell lung cancer.* Science, 2015. **348**(6230): p. 124-8.
10. Wolchok JD, Hoos A, O'Day S, et al. *Guidelines for the evaluation of immune therapy activity in solid tumors: immune-related response criteria.* Clin Can Res 2009;15:7412–20.
11. Wang Z, Aguilar EG, Luna JI, et al: Paradoxical effects of obesity on T cell function during tumor progression and PD-1 checkpoint blockade. Nat Med 25:141-151, 2019
12. Mariathasan S, Turley SJ, Nickles D, et al: TGFbeta attenuates tumour response to PD-L1 blockade by contributing to exclusion of T cells. Nature 554:544-548, 2018

13. Tsukamoto H, Fujieda K, Miyashita A, et al: Combined Blockade of IL6 and PD-1/PD-L1 Signaling Abrogates Mutual Regulation of Their Immunosuppressive Effects in the Tumor Microenvironment. Cancer Res 78:5011-5022, 2018
14. Shachar SS, Williams GR, Muss HB, et al: Prognostic value of sarcopenia in adults with solid tumours: A meta-analysis and systematic review. Eur J Cancer 57:58-67, 2016
15. Williams GR, Deal AM, Muss HB, et al: Skeletal muscle measures and physical function in older adults with cancer: sarcopenia or myopenia? Oncotarget 8:33658-33665, 2017
16. Mourtzakis M, Prado CM, Lieffers JR, et al: A practical and precise approach to quantification of body composition in cancer patients using computed tomography images acquired during routine care. Appl Physiol Nutr Metab 33:997-1006, 2008
17. van Vugt JL, Levolger S, Gharbharan A, et al: A comparative study of software programmes for cross-sectional skeletal muscle and adipose tissue measurements on abdominal computed tomography scans of rectal cancer patients. J Cachexia Sarcopenia Muscle 8:285-297, 2017
18. Martin L, Birdsell L, Macdonald N, et al: Cancer cachexia in the age of obesity: skeletal muscle depletion is a powerful prognostic factor, independent of body mass index. J Clin Oncol 31:1539-47, 2013
19. Li LF, Ma C. Epidemiological study of severe cutaneous adverse drug reactions in a city district of China. Clin Exp Dermatol. 2006;31(5):642-647
20. Yang MS, Lee JY, Kim J, et al. Incidence of Stevens-Johnson Syndrome and Toxic Epidermal Necrolysis: A Nationwide Population-Based Study Using National Health Insurance Database in Korea. PLoS One. 2016;11(11):e0165933
