# Supplementary material for: Atezolizumab in Combination With Carboplatin and Survival Outcomes in Patients With Metastatic Triple-Negative Breast Cancer: The TBCRC 043 Phase 2 Randomized Clinical Trial
Source: JAMA Oncol. 2023 Dec 14;10(2):193–201. doi: 10.1001/jamaoncol.2023.5424 (PMC10722391; doi:10.1001/jamaoncol.2023.5424)
Supplement: Supplement 2. — eAppendix eMethods eFigure 1. Trial Schema eFigure 2. Tumor response by treatment arm eFigure 3. PD-L1 IHC association with response eFigure 4. Intratumor and stromal tumor-infiltrating lymphocyte quantification and association with response eFigure 5. Body mass index and average blood glucose eFigure 6. RNA seq and TNBC subtyping eFigure 7. Mutational analysis of DNA-seq eFigure 8. Association of TP53, BRCA1/2 and PIK3CA mutations with survival eFigure 9. Multiplex-immunofluorescence staining of pretreatment tumor biopsies eTable 1. Demographics and tumor characteristics for patients enrolled in TBCRC 043 eTable 2. Treatment-related adverse events occurred in >5% of patients eTable 3. Clinical response and endpoints [file jamaoncol-e235424-s002.pdf]

Supplemental Online Content

Lehmann BD, Abramson VG, Dees EC, et al. Atezolizumab in combination with carboplatin and survival outcomes in patients with metastatic triple-negative breast cancer: the TBCRC 043 phase 2 randomized clinical trial. *JAMA Oncology*. Published online December 14, 2023. doi:10.1001/jamaoncol.2023.5424

eAppendix
eMethods
eFigure 1. Trial Schema
eFigure 2. Tumor response by treatment arm
eFigure 3. PD-L1 IHC association with response
eFigure 4. Intratumor and stromal tumor-infiltrating lymphocyte quantification and association with response
eFigure 5. Body mass index and average blood glucose
eFigure 6. RNA seq and TNBC subtyping
eFigure 7. Mutational analysis of DNA-seq
eFigure 8. Association of TP53, BRCA1/2 and PIK3CA mutations with survival
eFigure 9. Multiplex-immunofluorescence staining of pretreatment tumor biopsies
eTable 1. Demographics and tumor characteristics for patients enrolled in TBCRC 043
eTable 2. Treatment-related adverse events occurred in >5% of patients
eTable 3. Clinical response and endpoints

This supplemental material has been provided by the authors to give readers additional information about their work.

© 2023 Lehmann BD, et al. *JAMA Oncology.*

eMethods

Outcomes and statistical analysis

The primary endpoint of this randomized phase 2 trial was PFS, defined as the time from randomization to the earliest documented disease progression or death due to any cause.

The study was powered for the primary endpoint of PFS. A planned sample size estimation of 106 (n=53/arm) provided 80% power to detect a 1.5-month improvement in the median PFS with a one-sided significance (type I error= 0.10). Distributions of time-to-event variables were estimated with the use of the Kaplan–Meier product-limit method. The stratified log-rank test was used as the primary analysis for comparison of treatment groups.

Secondary endpoints were overall response rate (ORR), clinical benefit rate (CBR) and overall survival (OS). Patients receiving carboplatin + atezolizumab were discontinued from study medications upon disease progression by RECIST. Computed tomography (CT) scans were performed at baseline, approximately every 3 cycles while on treatment and at end-of-treatment. All scans were sent for central review at the Vanderbilt Cancer Imaging Support Laboratory, and changes to measurable target lesions were assessed by a radiologist (R.G.A.). All patients underwent a baseline core biopsy of a metastatic lesion (if reasonably safe), or prior biopsy or surgical tissue was retrieved before enrollment. Efficacy analyses were based on patients receiving ≥ 1 cycle. The overall response rate (ORR; CR + PR) and clinical benefit rate (CBR; CR + PR + stable disease ≥ 6 months) were determined along with corresponding 2-sided, exact binomial 95% confidence intervals (CIs). Hazard ratios and 95% confidence intervals were estimated with the Cox proportional hazards model.

© 2023 Lehmann BD, et al. *JAMA Oncololgy.*

Exclusion criteria

Patients were excluded if they were pregnant or lactating, had known CNS/leptomeningeal disease, significant cardiovascular disease, severe infection, had a history of autoimmune disease, history of idiopathic pulmonary fibrosis, uncontrolled hypercalcemia, active HIV or hepatitis B/C infection, systemic treatment with immunosuppressive medications, or prior treatment with systemic immunostimulatory agents or PD-1/PD-L1 targeting agents.

Demographic reporting

Demographic variables collected were collected from electronic health records (age and sex), and race was self-reported by study participants.

Tissue collection and processing

Metastatic pretreatment biopsies were either formalin-fixed and paraffin-embedded (FFPE) or placed into RNAlater (Thermo Fisher, AM7020) and stored in liquid nitrogen.

TIL assessment

Intratumoral and stromal tumor-infiltrating lymphocytes (TILs) were quantified according to the International TILs Working Group Guidelines on hematoxylin and eosin slides from pretreatment biopsies[14, 15].

PD-L1 Immunohistochemistry

PD-L1 status was determined in pretreatment FFPE samples using the PD-L1 (Ventana, SP142) immunohistochemical assay at a reference lab (CellCarta, ANTWERP, Belgium).

© 2023 Lehmann BD, et al. *JAMA Oncology.*

DNA sequencing

DNA was extracted from FFPE samples with sufficient tissue (n=93), underwent PCR-free library construction or low-input library construction, and was sequenced (PE150) at an average sequencing depth of 20X (BGI Americas). Raw sequences were aligned (bwa) to the human genome (hg38), duplicates removed, and SNP called according to GATK best practices recommendations[16]. The SNPs/indels were discarded with minor allele frequencies greater than 0.05 in ExAC, 1000G or gnomAD databases.

For patients with sufficient tissue (n=93), DNA was isolated (DNAeasy, Qiagen) from FFPE tissue, DNA was quantified by Qubit fluorometer (Thermo Fisher Scientific) and either underwent PCR-free library construction (n=50) or low-input library construction (n=43). DNA libraries underwent PE150 sequencing (BGI Americas) performed on a DNBseq platform (MGI) at an average sequencing depth of 20X. Sequence quality of the paired-end reads with FastQC (v.0.10.0) for each of 93 whole-genome samples. Raw sequences were aligned (bwa mem v.0.7.17) to the human genome (hg38) with '-K 100000000 -v 3 -M' option, and the remaining parameters were set to default. Duplicates were removed using Sambamba, and the Genome Analysis Toolkit (GATK) was applied to the remaining reads. Base quality score recalibration, SNP, indel discovery, and genotyping were performed simultaneously using standard hard-filtering parameters according to GATK best practices recommendations. Two additional parameters, "--soft-clip-low-quality-ends true --dont-use-soft-clipped-bases true," were used in HaplotypeCaller. SNPs and indels were annotated using ANNOVAR. The SNPs and indels with minor allele frequencies greater than 0.05 in any one of the ExAC, 1000 G and gnomAD databases were discarded. A summary report of somatic mutations, insertions-deletions, structural variants, copy number, and mutational signatures was generated by the R package "mafreport."

© 2023 Lehmann BD, et al. *JAMA Oncology.*

Somatic copy number

GATK gCNV pipeline was used for copy number variation prediction. Input HDF5 files for GATK gCNV were generated using GATK's CollectReadCounts utility with the interval merging rule set to "OVERLAPPING_ONLY." ENCODE hg38 blacklist v2 was used in FilterIntervals. DetermineGermlineContig Ploidy and GermlineCNVCaller were run in COHORT mode. All analysis steps, including generating the output VCF files via PostprocessGermline CNVCalls, were run with GATK v4.2.2.0. A custom script was used to combine output VCFs to Gistic2 segment file and then applied to Gistic2. Gene-level CNAs were extracted from "all_thresholded.by_genes.txt' and plotted with oncoplot.

RNA sequencing

Total RNA was extracted (Quick-RNA, Zymo Research, R1054) from frozen tumor biopsies. NGS libraries were prepared from Ribo-depleted RNA and sequenced (150PE) on an Illumina NovaSeq6000. Isolated RNA was quantified using the Qubit RNA HS Assay Kit (Life Technologies, catalog no. Q32852) and assessed with the Agilent 2100 bioanalyzer.

Raw reads were aligned to the hg38 genome using the STAR aligner 2-pass method, and gene-level read counts were quantified using subREAD. Count level data was corrected for batch effects from fixation and metastatic tissue with the ComBat_seq function (sva v3.35.2). Count level data were corrected for batch effects from fixation, metastatic tissue adjusted count data were normalized, and differentially expressed genes were identified using DESeq2 (v1.30.1), correcting for extraction method and metastatic site.

TNBCtype subtyping

© 2023 Lehmann BD, et al. *JAMA Oncology.*

Normalized, batch-corrected, log2-transformed RNA expression were used to determine TNBCtype (http://cbc.mc.vanderbilt.edu/tnbc/) as previously described[17]. The highest correlation coefficients were used to assign subtypes to either basal-like 1 (BL1), basal-like 2 (BL2), mesenchymal (M) or luminal androgen receptor (LAR) subtype.

Time-dependent blood glucose Cox regression analysis

A time-dependent Cox regression model was applied to compare patients' survivals between two treatment arms: Carboplatin alone and Atezolizumab + Carboplatin, after adjusting for their time-dependent blood glucose trends (per 40 units increase). The blood glucose trend was a time-dependent continuous variable that varied with time during patients' follow-up, and the patients' survivals of interest were progression-free survival (PFS) and overall survival (OS). The hazard ratio (HR) was used to evaluate the association. HR < 1 (HR > 1) denotes that the higher the glucose trend, the better (the worse) the PFS/OS.

Multiplexed immunofluorescence (MxIF)

FFPE tissue sections (4μm) underwent deparaffinization, antigen retrieval and protein block. Sections were incubated with the primary antibodies: anti-panCK, anti-PD-L1, anti-CD4, ant-CD8, anti-FOXP3, anti-GZMB, or anti-HLA-A and counterstained with DAPI for nuclei identification (see supplemental methods for details). Whole slide images were digitally acquired (AxioScan Z1, Carl Zeiss), and automated quantification was performed through a pathologist-supervised (M.E.S. and P.I.G-E) machine learning.

Assays were developed and optimized using tyramine signal amplification (TSA) for increased antigen sensitivity. Antibody testing was performed on control tissues with chromogenic and fluorescence immunohistochemistry (IHC) to ensure expression patterns corresponded to the biologically expected distribution. Antibody-fluorophore pairing and order within the multiplex assay were selected based on the highest signal-to-noise ratio and expression patterns compared

© 2023 Lehmann BD, et al. *JAMA Oncology.*

to single chromogenic IHC. Sections were incubated with the secondary antibody, and TSA reagent (Tyramide Super boost Kit, Invitrogen) applied according to manufacturer's recommendations and counterstained with DAPI for nuclei identification.

Ab Target	Clone	Catalog	Company	Dilution
B7H4	D1M8I	14572	Cell Signaling	1:200
CD4	EP204	RM27-10	StatLab	prediluted
CD8	144B	MM39-10	StatLab	prediluted
FOXP3	D2W8E	98377	Cell Signaling	1:100
GZMB	11F1	API3202AA	BioCare	1:100
HLA-A	C-6	sc-365485	Santa Cruz	1:1300
HLA-DR	TAL1B5	sc-53319	Santa Cruz	1:1000
panCk	AE1/AE3	CM011A	Biocare	1:600
PDL1	73-10	ab228415	Abcam	1:400

Image analysis and quantification

Whole slide images were digitally acquired using an AxioScan Z1 slide scanner (Carl Zeiss) at 20x. Automated quantification was performed through a pathologist-supervised machine learning algorithm using QuPath software. Cell segmentation was determined on DAPI. Object classifiers were trained on annotated training regions from control and tumor tissues to define cellular phenotypes. Tumor cells were defined by pan-cytokeratin (CK) expression and subcellular characteristics. Batch analysis was performed using the optimized algorithm. Tumor areas were manually annotated for cases with low, heterogenous, or null CK expression in which the classifier performance was not optimal. Out-of-focus areas, tissue folds, necrosis and normal tissue were excluded from the analysis. The study pathologists (M.E.S. and P.I.G-E) visually assessed each sample for the correct performance of the quantification algorithm. CK expression on tumor cells was quantified as % of positive tumor cells and as cell density (/mm^2) for immune cells. The combined positive score (CPS) is calculated as the number of PD-L1 staining cells (tumor cells, lymphocytes, macrophages) divided by the total number of viable tumor cells multiplied by 100.

© 2023 Lehmann BD, et al. *JAMA Oncology.*

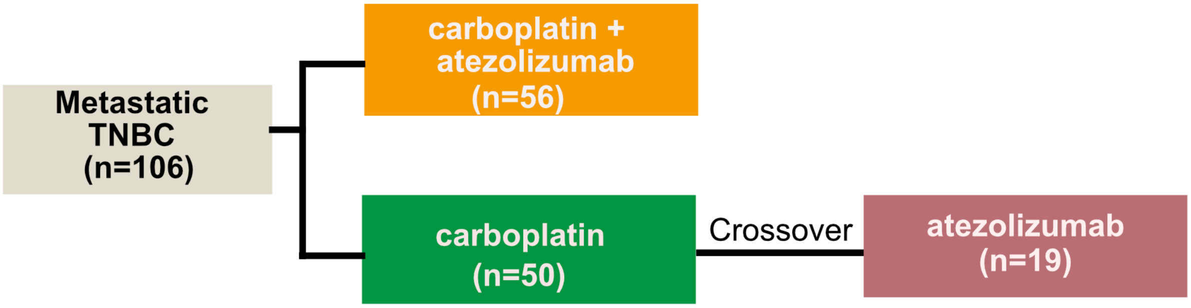

eFigure 1. Trial Schema.

© 2023 Lehmann BD, et al. *JAMA Oncology.*

eFigure 2. **Tumor response by treatment arm.** (A) Waterfall graph of percentage change in total sum of target lesion diameters by RECIST from baseline to first radiologic evaluation. (B) Duration of response for responding patients stratified by treatment arm. (C) Overall survival of patients who progressed on carboplatin and crossed over to atezolizumab monotherapy compared to patients receiving carboplatin + atezolizumab simultaneously. The data cut-off was October 2021.

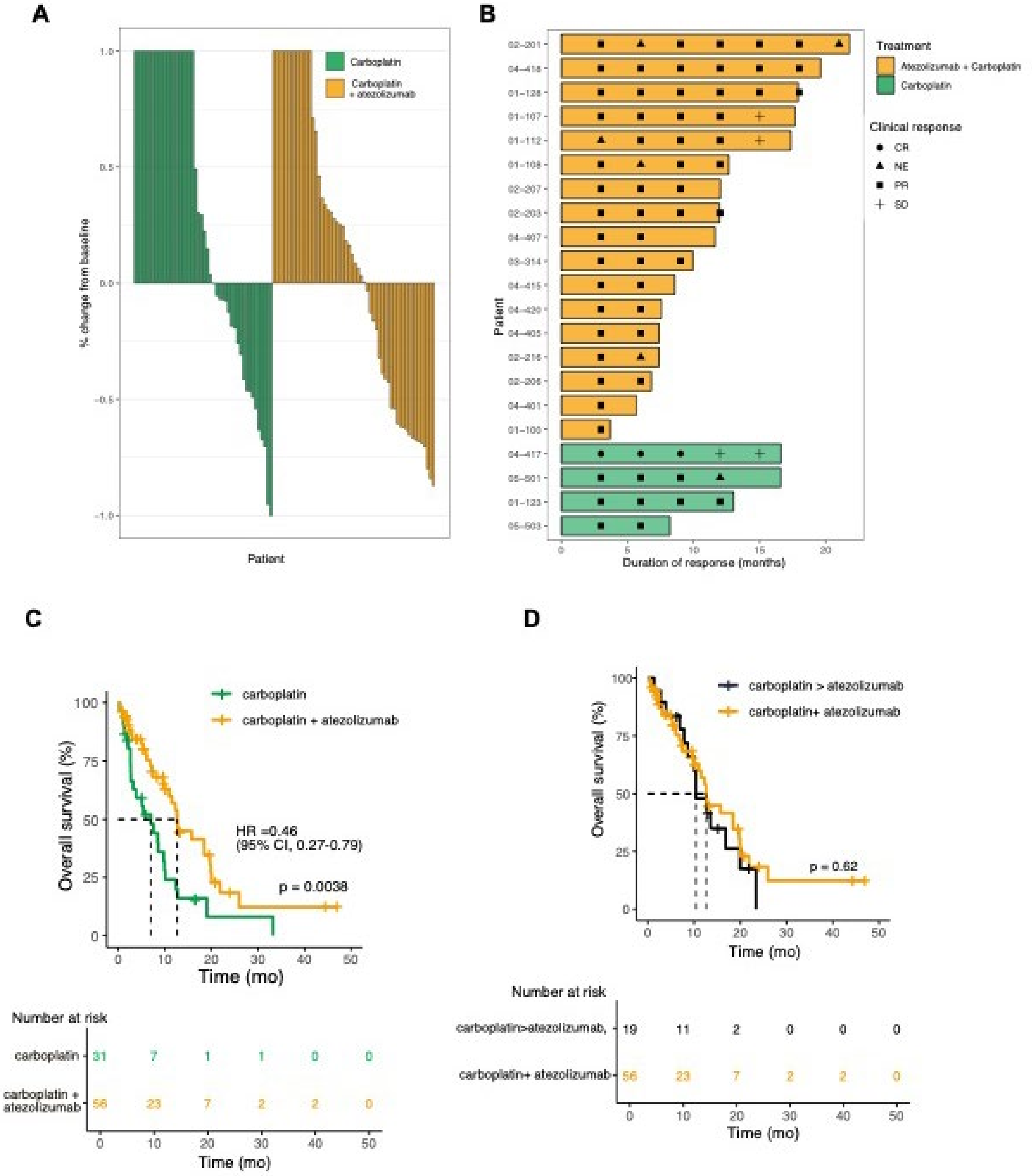

© 2023 Lehmann BD, et al. *JAMA Oncology.*

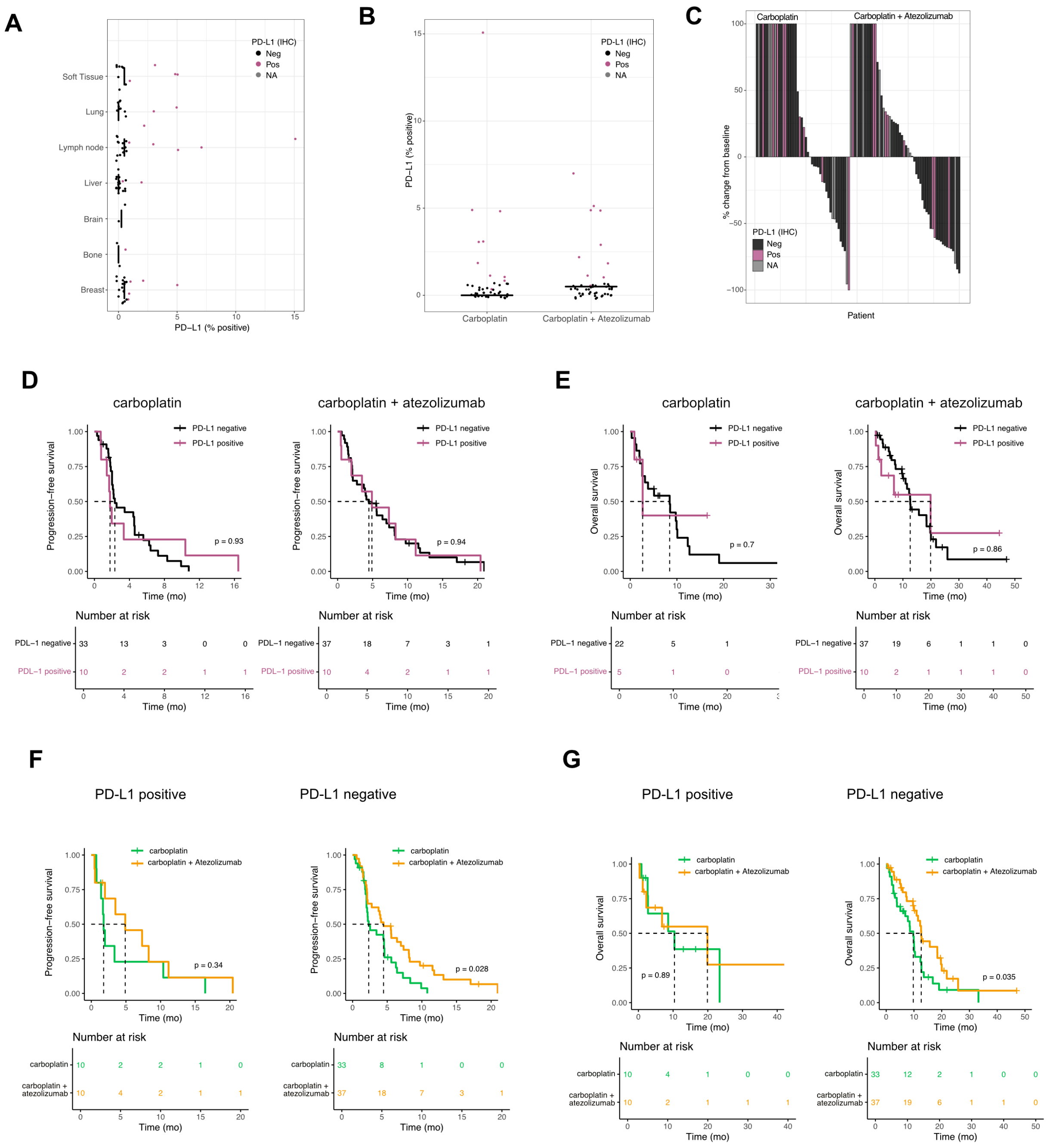

eFigure 3. PD-L1 IHC association with response. (A) Dot plot shows stromal PD-L1 IHC (% positive) by metastatic biopsy site. (B) Plot shows PD-L1 (% positive tumor/stroma) IHC by treatment arm. (C) Waterfall plot shows the percentage change in the total sum of target lesion diameters by RECIST colored by PD-L1 IHC status. Kaplan-Meier plots show (D) progression-free survival and (E) overall survival for patients on each treatment arm stratified by PD-L1 status or (F) PFS and (G) OS in PD-L1 positive and negative patients stratified by treatment.

© 2023 Lehmann BD, et al. *JAMA Oncology.*

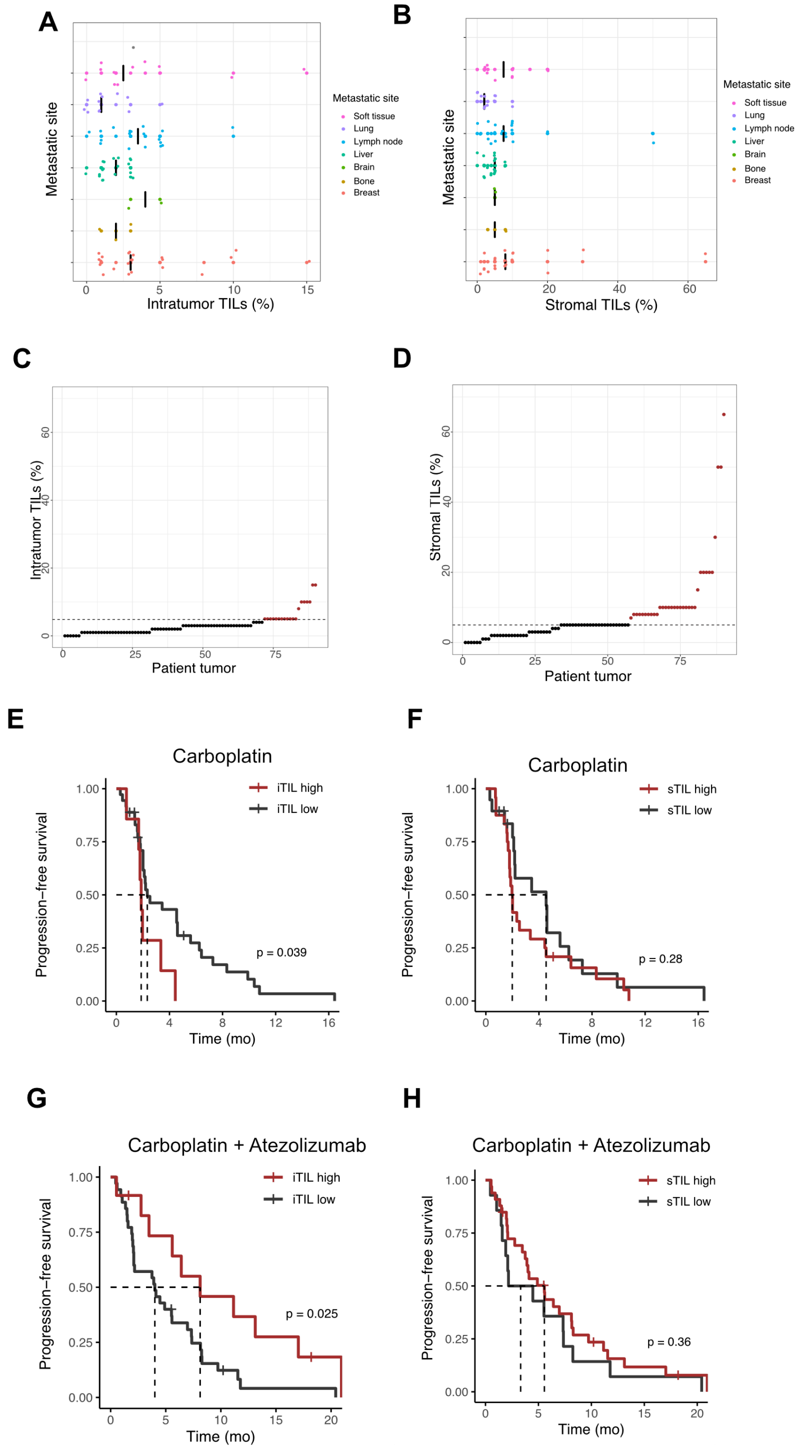

eFigure 4. Intratumor and stromal tumor-infiltrating lymphocyte quantification and association with response. Scatterplots show (A) intratumoral (iTIL) and (B)stromal (sTIL) tumor-infiltrating lymphocytes (% total cells) by metastatic biopsy site. Scatterplots show (C) iTIL and (D) sTIL distribution and cut-offs. Kaplan-Meier plots show progression-free survival of patients treated with carboplatin (E and F) or carboplatin + atezolizumab (G and H) stratified by intratumor and stromal TIL levels.

© 2023 Lehmann BD, et al. *JAMA Oncology.*

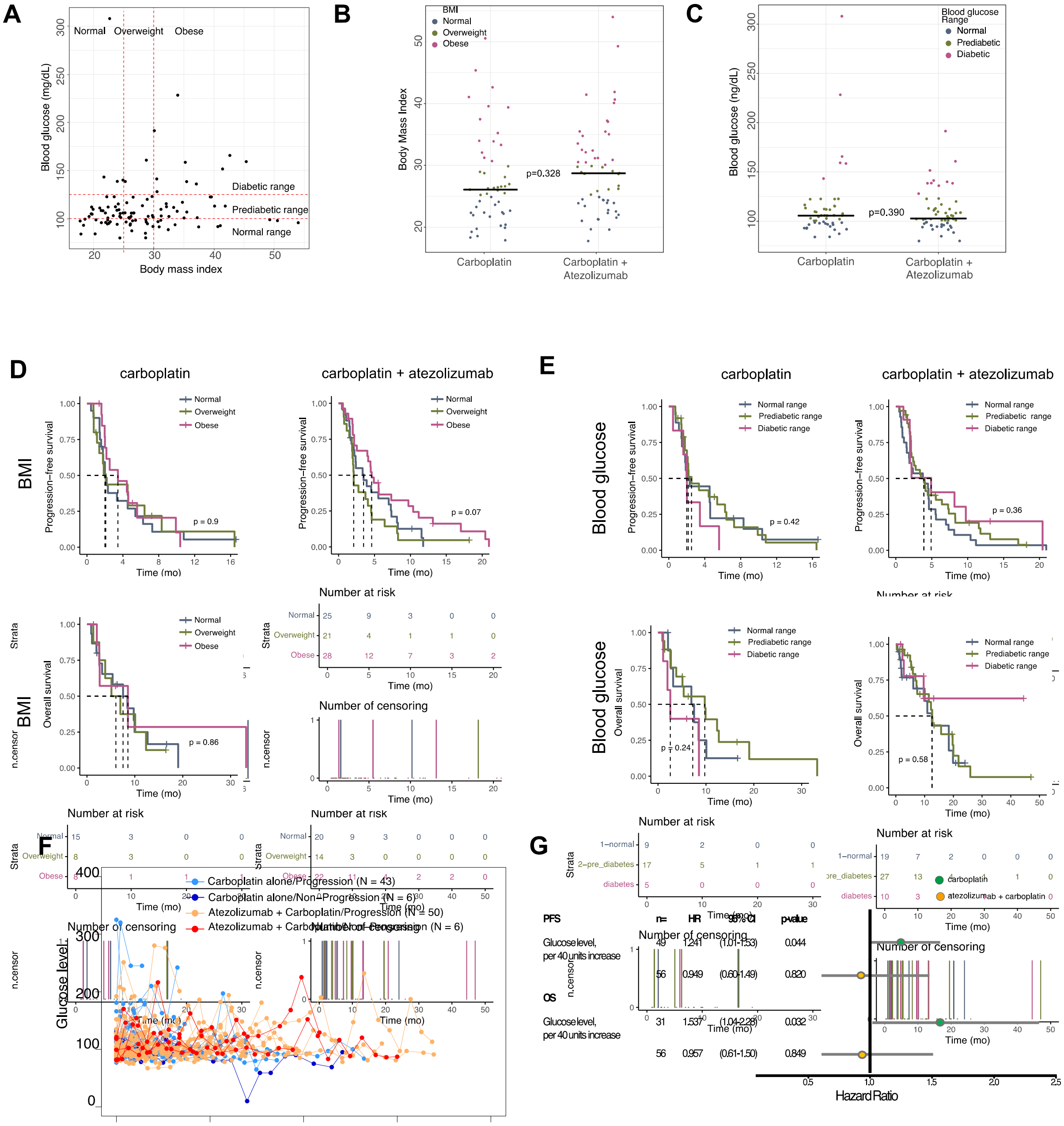

eFigure 5. Body mass index and average blood glucose. (A) Scatterplot shows patients' body mass indices (BMI) and average blood glucose levels during treatment. Dashed red lines indicate stratification point for BMI (<25, normal; >25 and <30 overweight; >30 obese) and blood glucose (<100mg/dL, normal; >100 and <125 mg/dL, prediabetic; >125 mg/dL, diabetic) ranges. Scatterplot show (B) BMI and (C) blood glucose range stratified by arm. Significance determined by t-test. Kaplan-Meier plots show progression-free survival and overall survival for patients stratified by (D) BMI range and (E) average blood glucose range during treatment.

© 2023 Lehmann BD, et al. *JAMA Oncology.*

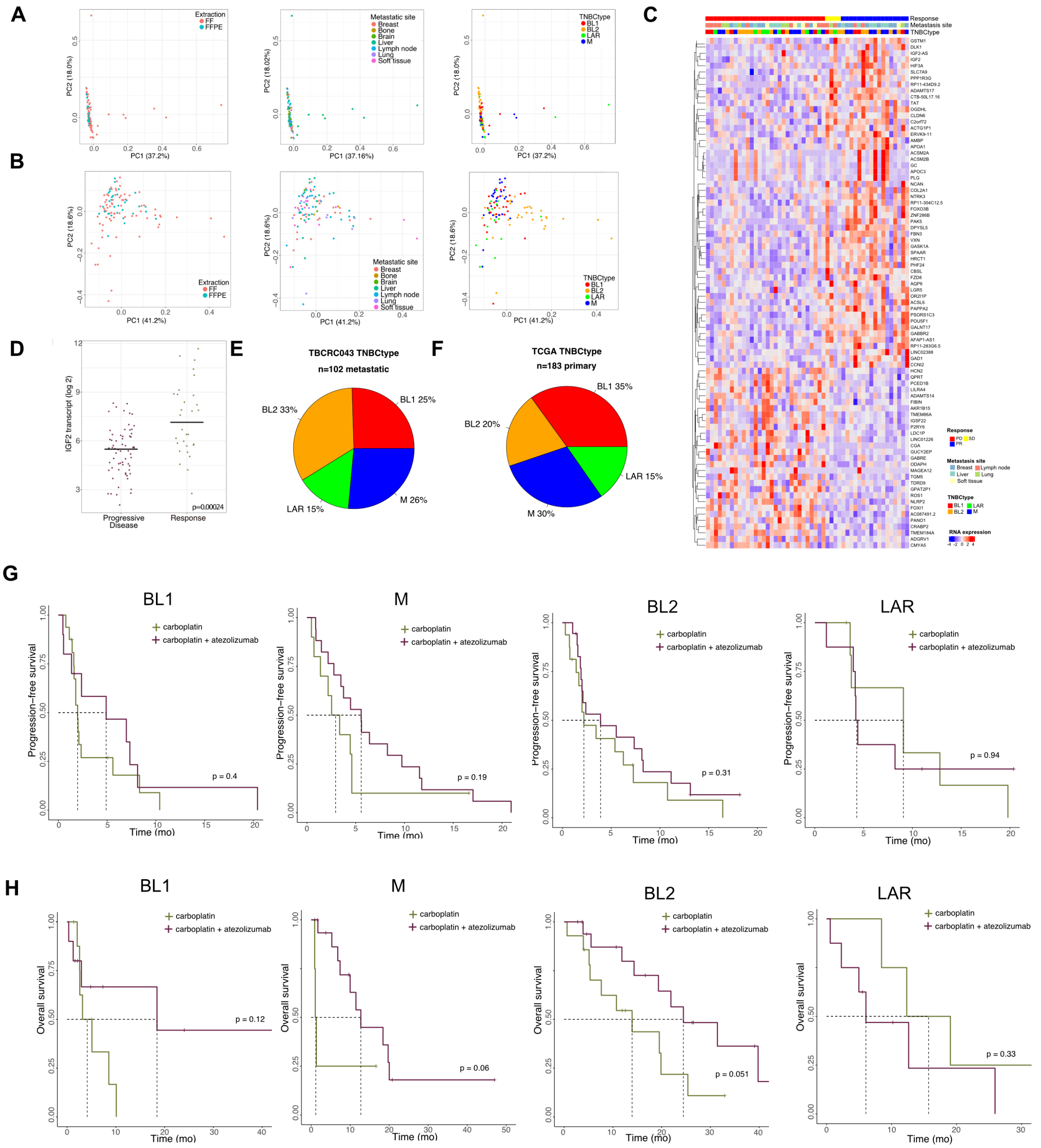

eFigure 6. RNA seq and TNBC subtyping. (A) Uncorrected and (B) batch-corrected principal component plots for colored by extraction method, metastatic biopsy site and TNBC subtype. (C) Heatmap of differentially expressed genes between patients responding to carboplatin + atezolizumab. Color bars indicate response (PR, partial response; SD, stable disease; RD, residual disease), metastatic biopsy site and TNBC subtype (BL1, basal-like 1, BL2, basal-like 2, M, mesenchymal; LAR, luminal androgen receptor). (D) Scatterplots show individual levels of IGF2 transcript by response. Pie charts show the distribution of TNBC subtypes in (E) metastatic TBCRC043 TNBC cohort and (F) primary TNBC in TCGA. Kaplan-Meier plots show (G) progression-free survival and (H) overall survival for each TNBC subtype stratified by treatment arm.

© 2023 Lehmann BD, et al. *JAMA Oncology.*

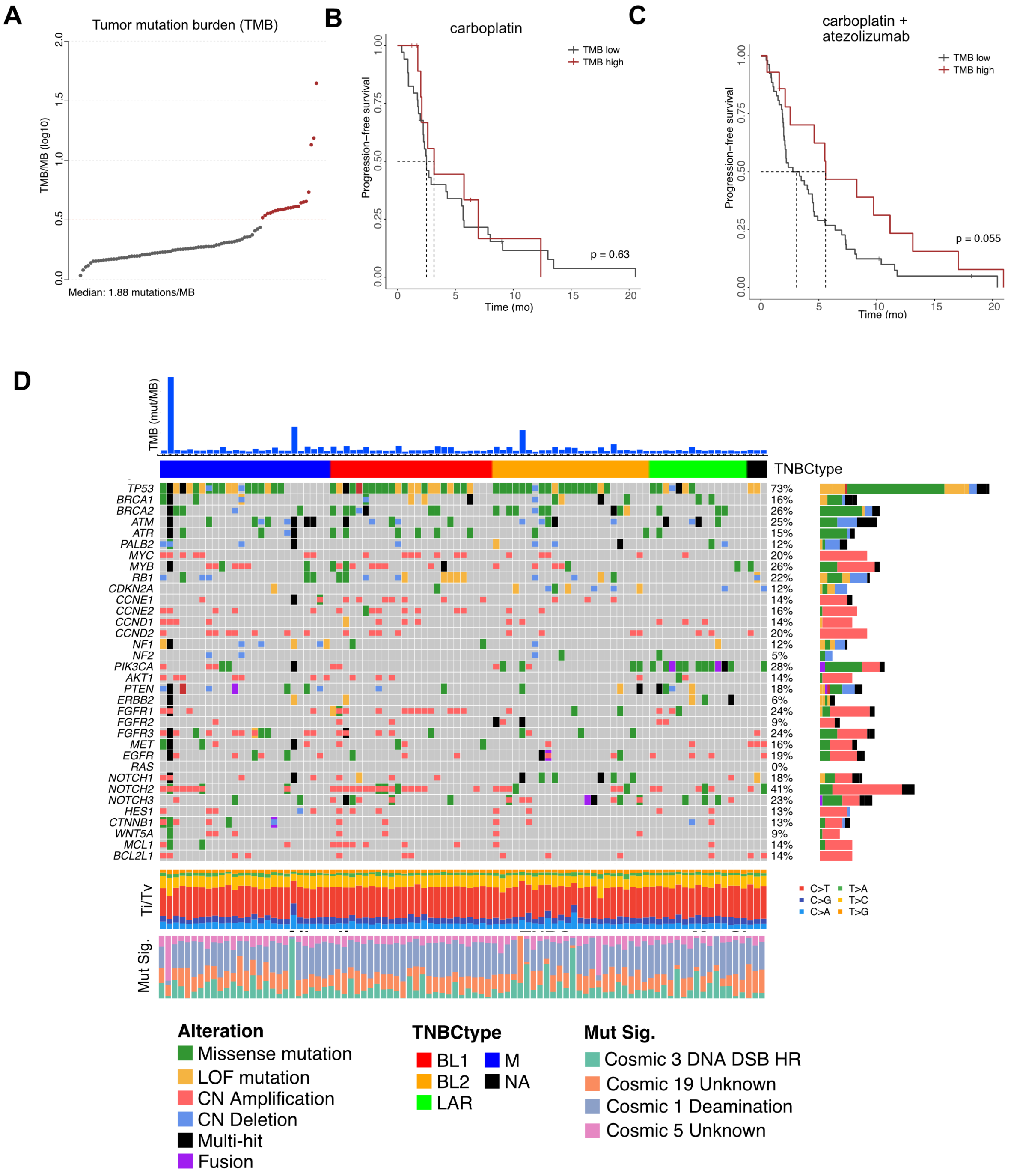

eFigure 7. Mutational analysis of DNA-seq. (A) Plot shows ranked pretreatment tumor mutation burden (mutations/MB). The dashed red line indicates the cut-point for TMB high (red) and low (black). Kaplan-Meier survival plots for patients treated with (B) carboplatin or (C) carboplatin + atezolizumab stratified by TMB status. (D) Oncoplot shows TMB, trinucleotide mutational signatures, mutations, copy number (CN) alterations, and gene fusions in individual patient tumors grouped into TNBC subtypes. LOF, loss-of-function; CN, copy number; DSB, double-strand break; HR, homologous recombination.

© 2023 Lehmann BD, et al. *JAMA Oncology.*

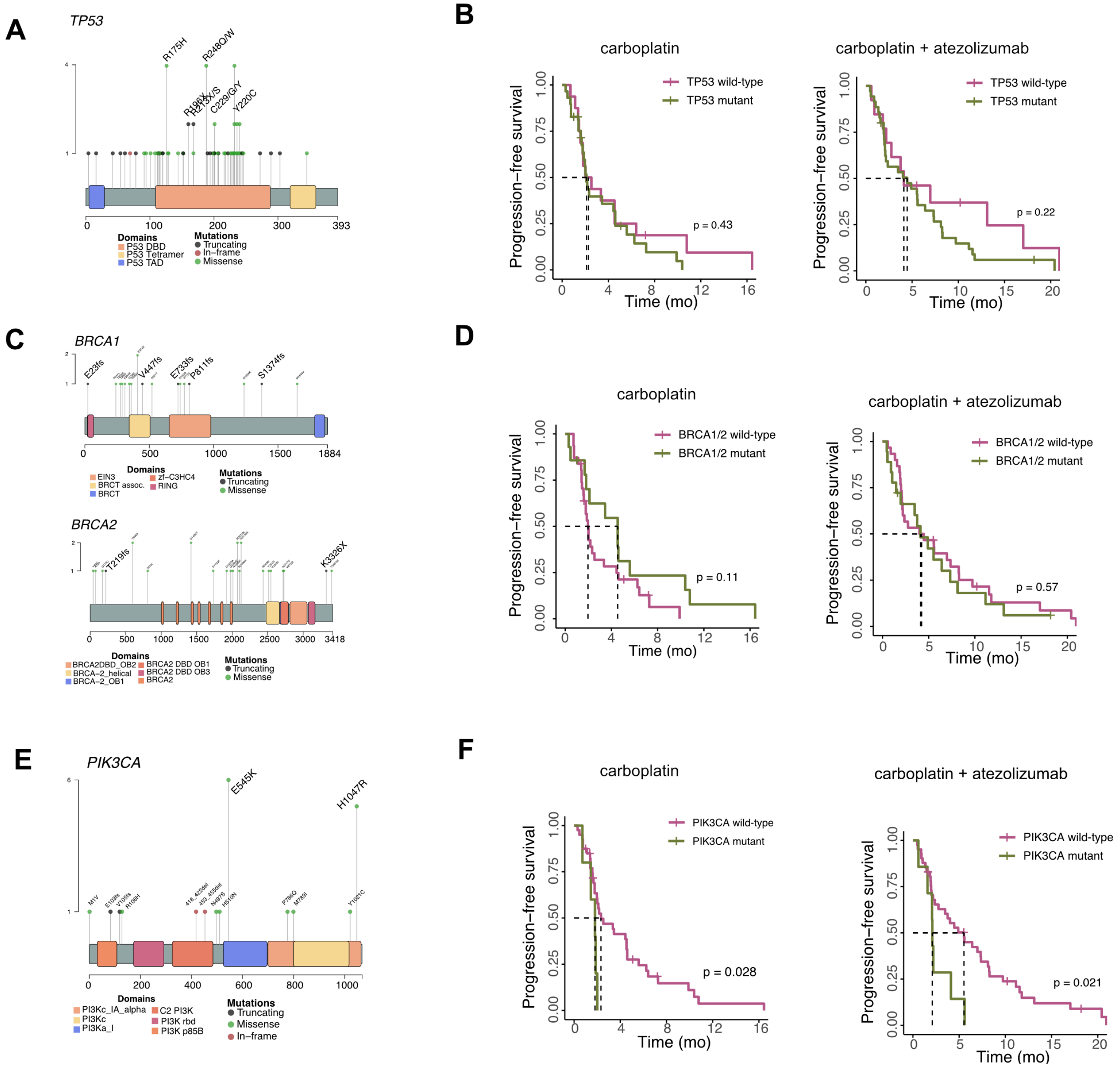

eFigure 8. Association of TP53, BRCA1/2 and PIK3CA mutations with survival. Lollipop plots show protein domain mutations for (A) TP53, (C) BRCA1 and BRCA2, and (E) PIK3CA. Kaplan-Meier survival plots show PFS for carboplatin and carboplatin + atezolizumab arms stratified by (B) TP53, (D) BRCA1/2, or (F) PIK3CA mutation status.

© 2023 Lehmann BD, et al. *JAMA Oncology.*

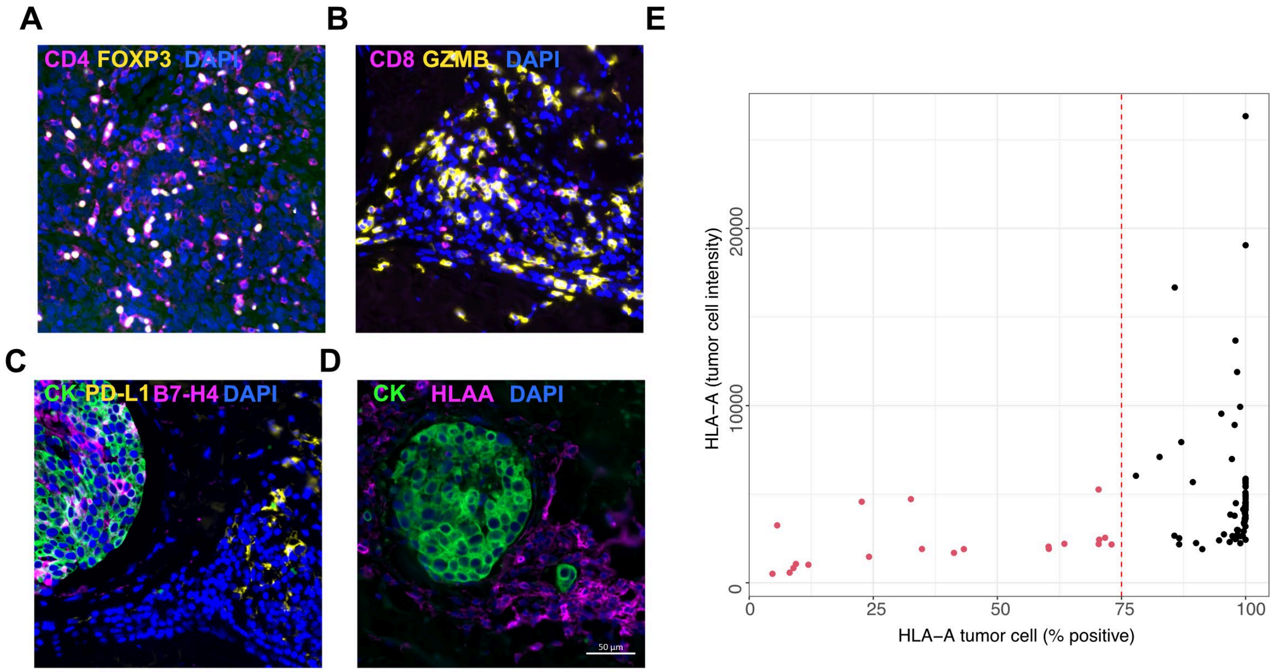

eFigure 9. Multiplex-immunofluorescence staining of pretreatment tumor biopsies. Representative images of tumor stained for (A) CD4 (red) and FOXP3 (yellow), (B) CD8 (red) and GZMB (yellow), (C) CK5 (green), PD-L1 (red) and B7-H4 (cyan) or (D) CK5 (green) and HLA-A (purple). All nuclei were counterstained with DAPI. (E) The scatterplot shows HLA-A quantification by % positive tumor cells and integrated intensity in tumors from patients enrolled in the study. HLA-A low (red) tumors were defined as those with less than 75% tumor cells positive for HLA-A.

© 2023 Lehmann BD, et al. *JAMA Oncology.*

eTable 1. Demographics and tumor characteristics for patients enrolled in TBCRC 043

Arm		Carboplatin (N=50)	Carboplatin + Atezolizumab (N=56)	Combined (N=106)
Age on study		55	56	55
Race*				
	African American/Black	24% (12)	14% (8)	19% (12)
	Asian	0% (0)	2% (1)	1% (1)
	White	62% (31)	75% (42)	69% (73)
	Unknown	14% (7)	9% (5)	10% (11)
Metastatic site				
	Bone	33% (15)	39% (21)	36% (36)
	Brain	8% (4)	4% (2)	6% (6)
	Chest wall	0% (0)	4% (7)	4% (4)
	Liver	33% (15)	37% 20)	4% (4)
	Lymph node	63% (29)	46% 25)	54% 54)
	Lung	50% (23)	50% (27)	50% (50)
	Soft tissue	13% (6)	5% (3)	9% (9)
	Skin	17% (8)	4% (2)	10% (10)
PD-L1 IHC				
	Positive	20% (10)	18% (10)	19% (20)
	Negative	66% (33)	66% (37)	66% (70)
	NE	14% (7)	16% (9)	15% (16)
BRCA1/2 Mutation				
	Wild-type	62% (31)	53% (30)	66% (61)
	Mutant	25% (14)	32% (18)	34% (32)
	NE	10% (5)	14% (8)	12% (13)
TNBCtype				
	Basal-like 1 (BL1)	26% (13)	21% (12)	24% (25)
	Basal-like 2 (BL2)	38% (19)	30% (17)	34% (36)
	Luminal AR (LAR)	14% (7)	16% (9)	15% (16)
	Mesenchymal (M)	18% (9)	25% (14)	22% (23)
	NE	4% (2)	7% (4)	6% (6)
TILs				
	Intratumor	2%	3%	3%
	Stromal	5%	5%	5%

© 2023 Lehmann BD, et al. *JAMA Oncology.*

Any prior chemotherapy		76% (38)	88% (49)	82% (87)
Any prior chemotherapy neo/adjuvant		70% (35)	71% (40)	66% (75)
Any prior metastatic chemotherapy		24% (12)	39% (22)	32% (34)
Prior platinum (neo-/adjuvant)		20% (10)	9% (5)	14% (15)

IHC, immunohistochemistry; TILs, tumor-infiltrating lymphocytes; NE, not evaluable. *Race was self-reported by the participants.

© 2023 Lehmann BD, et al. *JAMA Oncology.*

eTable 2. Treatment-related adverse events occurring in >5% of patients.

		Carboplatin (N=50)		Carboplatin + atezolizumab (N=56)		Carboplatin crossover > atezolizumab (N=19)	
		Grade		Grade		Grade	
Category	Toxicity	2	3/4	2	3/4	2	3/4
Blood and lymphatic							
	Anemia			15 (27%)	2 (4%)		1 (5%)
	Lymphocytopenia			3 95%)	4 (7%)		
	Neutropenia	1 (2%)	1 (2%)	9 (16%)	7 (13%)		
	Thrombocytopenia		1 (2%)	10 (18%)	9 (16%)	1 (5%)	
Gastrointestinal disorders							
	Nausea	1 (2%)		5 (9%)		1 (5%)	
General disorders							
	Fatigue	1 (2%)		10 (18%)	1 (2%)		
Labs							
	AST increased			3 (5%)	1 (2%)		
Possible immune-related AE				9 (16%)	3 (5%)	2 (10%)	1 (5%)

Data are No. (%)
AST, aspartate aminotransferase

eTable 3. Clinical response and endpoints.

**Clinical Response**					
	**Complete response N (%)**	**Partial response N (%)**	**Stable disease N (%)**	**Progressive disease N (%)**	
Carboplatin	1 (2.2)	3 (6.7)	5 (11.1)	36 (80.0)	
Carboplatin + Atezolizumab	0 (0.0)	17 (31.4)	4 (7.4)	33 (61.1)	
**Endpoints**					
	**Primary**	**Secondary**			
	**Progression-free survival mo (95%CI)**	**Overall response rate % (95% CI)**	**Clinical benefit rate % (95% CI)**	**Duration of response mo (95%CI)**	**Overall survival mo (95%CI)**
Carboplatin	2.2 (2.0- 4.4)	8.0 (3.2-.18.8)	18.0 (9.8-30.1)	14.8 (8.2- Inf)	8.6 (6.9-12.3)
Carboplatin + Atezolizumab	4.1 (2.4-7.0)	30.4 (19.9-43.3)	37.5 (26.0-50.6)	11.6 (7.6-17.7)	12.6 (11.0-19.9)

Overall response rate = CR + PR
Clinical benefit rate = CR + PR + SD (≥ 6 months)

© 2023 Lehmann BD, et al. *JAMA Oncology.*
