# Supplementary material for: Atezolizumab in Combination With Carboplatin and Survival Outcomes in Patients With Metastatic Triple-Negative Breast Cancer: The TBCRC 043 Phase 2 Randomized Clinical Trial
Source: JAMA Oncol. 2023 Dec 14;10(2):193–201. doi: 10.1001/jamaoncol.2023.5424 (PMC10722391; doi:10.1001/jamaoncol.2023.5424)
Supplement: Supplement 4. — Data Sharing Statement [file jamaoncol-e235424-s004.pdf]

Data Sharing Statement

Lehmann. Atezolizumab in Combination With Carboplatin and Survival Outcomes in Patients With Metastatic Triple-Negative Breast Cancer. *JAMA Oncol*. Published December 14, 2023.
doi:10.1001/jamaoncol.2023.5424

Data
Data available: Yes
Data types: Deidentified participant data
How to access data: Supplemental material and on SRA
When available: With publication

Supporting Documents
Document types: None

Additional Information
Who can access the data: anyone requesting the data
Types of analyses: or any purpose
Mechanisms of data availability: without investigator support
